# Integrated Strategy of UHPLC-Q-TOF-MS and Molecular Networking for Identification of Diterpenoids from *Euphorbia fischeriana* Steud. and Prediction of the Anti-Breast-Cancer Mechanism by the Network Pharmacological Method

**DOI:** 10.1155/2021/3829434

**Published:** 2021-11-11

**Authors:** Tian-Cheng Ma, Yu-Kun Ma, Jin-Ling Zhang, Lei Liu, Jia Sun, Li-Na Guo, Qi Liu, Yu Sun

**Affiliations:** ^1^Research Institute of Medicine and Pharmacy, Qiqihar Medical University, Bukui Road 333, Qiqihar 161006, Heilongjiang, China; ^2^School of Traditional Chinese Materia Medica, Shenyang Pharmaceutical University, Wenhua Road 103, Shenyang 110016, Liaoning, China

## Abstract

Breast cancer is one of the most common malignancies in women worldwide. Traditional Chinese medicine has been used as adjunctive or complementary therapy for breast cancer. Diterpenoids from *Euphorbia fischeriana* Steud. have been demonstrated to possess anti-breast-cancer activity. This research was aimed to systematically explore the diterpenoids from *E. fischeriana* and study the multiple mechanisms on breast cancer. The structures of diterpenoids were identified by the integrated strategy of UHPLC-Q-TOF-MS and molecular networking. A total of 177 diterpenoids belonging to 13 types were collected. In silico ADME analysis was performed on these compounds. It indicated that 130 of 177 diterpenoids completely adjusted to Lipinski's rule. The targets of compounds were obtained from PharmMapper. The targets of breast cancer were collected from GeneCards. Then, 197 compounds-related targets and 544 breast cancer-related targets were identified. After the intersection process, 58 overlapping targets between compounds-related targets and breast cancer-related targets were acquired. The STRING database was applied to predict the protein-protein interactions. The GO and KEGG pathway enrichment analysis were performed by using the KOBAS database. It indicated that these predicted pathways were closely related to breast cancer. The treatment effect of *E. fischeriana* on breast cancer might be performed through signaling pathways, such as IL-17 signaling pathway, MAPK signaling pathway, and PI3K-Akt signaling pathway. The predicted top genes such as EGFR, ESR, MAPK, SRC, CASP3, CDK2, and KDR were involved in cell proliferation, gene transcription, apoptosis, signal transduction, DNA damage and repair, tumor differentiation, metastasis, and cell cycle, which indicated that *E. fischeriana* might treat breast cancer comprehensively. A compounds-KEGG pathways-related targets network was built by using cytoHubba to analyze the hub compounds and targets. It concluded that *E. fischeriana* treated breast cancer not only by the main components but also by the microconstituents, which reflected the overall regulatory role of multicomponents treating breast cancer. To estimate the binding affinities, binding sites, and binding postures, molecular docking simulations between 177 diterpenoids and top 19 targets were carried out. The results are basically in line with expectations. In conclusion, these results can serve as references for researchers studying potential targets of diterpenoids from *E. fischeriana* on breast cancer in the future.

## 1. Introduction

Breast cancer represents the most common malignancy in women worldwide. Globally in 2020, 2,260,000 new cases were diagnosed and there were 680,000 breast cancer-associated deaths. On the molecular level, breast cancer belongs to a heterogeneous disease. It is often classified into diverse molecular subtypes on the basis of protein expression of estrogen receptor (ER), progesterone receptor (PR), and human epidermal growth factor receptor 2 (HER2). Triple-negative breast cancer, which is lack of ER, PR, and HER2 expressions, has the highest probability of metastasis and the lowest overall survival among all breast cancer subtypes [1]. In the recent decade, various therapeutic techniques including surgery therapy, radio therapy, chemo therapy, hormonal therapy, and targeted therapy have been used. The innovative anticancer drugs act effectively; though, patients still suffer from consequences of relapse and metastasis [2].

Nowadays, it is well known that some natural compounds obtained from traditional Chinese medicine are useful to treat cancer. *E. fischeriana*, belonging to the family of *Euphorbiaceae*, is a perennial herbaceous plant, which has been used as traditional Chinese medicine for thousands of years [3]. Chemical investigations of *E. fischeriana* have revealed the presence of diterpenoids, triterpenes, steroids, and aromatic tannins [4]. Diterpenoids possess isoprene or isopentane type skeleton, which are considered the main active ingredients of *E. fischeriana* [5]. Diterpenoids have been reported to have diversity in biological activity, such as antibacterial effect and tuberculosis effect. Research has revealed that many of the isolated diterpenoids from *E. fischeriana* possess excellent antitumor activities, especially on breast cancer [6].

**Figure 1:**
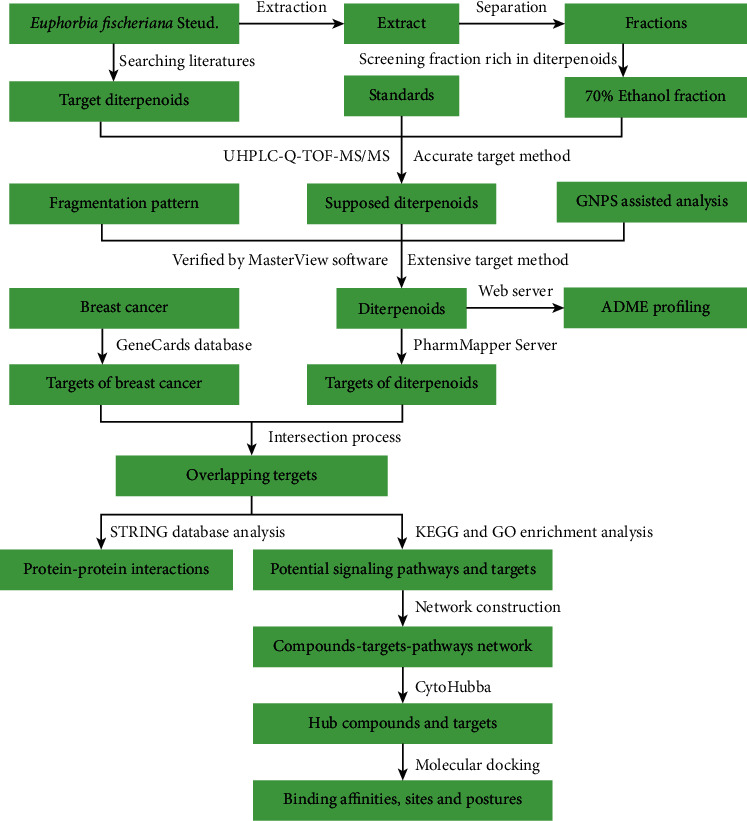
The workflow of this article.

Comprehensive identification of diterpenoids in *E. fischeriana* is extremely difficult for the complexity of structures. But it is critical for understanding its biological mechanism and establishing quality control protocols. Ultra-high-performance liquid chromatography coupled with Q-TOF mass spectrometry (UHPLC-Q-TOF-MS) is a powerful technique for compound identification in which the exact masses of compounds and elemental formulas based on the constituent atoms can be acquired [7]. Recently, Gerwick [8] and co-workers developed Global Natural Products Social Molecular Networking (GNPS), which could accelerate the progress of discovery of novel natural products. GNPS is an emerging visual molecular network, which allows rapid comparison of complex extracts by MS profiles. Network pharmacology is a research field in which it integrates a series of disciplines including pharmacology, bioinformatics, chemoinformatics, and systems biology. Meanwhile, it is an efficient and time-saving approach to identifying the potential targets and pathways for the drugs against the diseases by computational methods [9]. Nowadays, there was no research on the network pharmacology of *E. fischeriana*, and the mechanism of *E. fischeriana* in treating breast cancer was not very clear. To search for the potential targets of diterpenoids on breast cancer, network pharmacology prediction was used in this study. The workflow of this article is shown in Figure 1.

## 2. Materials and Methods

### 2.1. Chemicals and Reagents

The rhizomes of *E. fischeriana* were purchased from Xianhe Pharmaceutical Company and verified as genuine ones by Professor Guo Lina of Qiqihar Medical University. Acetonitrile and methanol were bought from Merck Company. Ethanol and formic acid were purchased from Tianjin Commie Chemical Reagent Co. Ltd.

### 2.2. Extraction and Separation Procedure

First, dried rhizomes of *E. fischeriana* were powdered using a pulverizer and then sieved into a homogeneous size (60 mesh). Then, 5.0 g powders were soaked with methanol and extracted for 1.0 h by using the ultrasound method. Next, the extracted solution was filtered and concentrated by rotary evaporation at 50°C. Last, in order to enrich diterpenoids, the extract was applied to a D-101 macroporous resin column in which 0%, 40%, 70%, and 95% ethanol fractions were obtained.

### 2.3. UHPLC-MS Detection

UHPLC-MS analyses were performed using the UHPLC system (Shimadzu, Japan) with a model of LC-30AD pump and a model of SIL-30AC autosampler that were connected to MS analysis instrument with a TripleTOF 4600 system (AB SCIEX, USA). Separation was carried out on a Waters column (ACQUITY UPLC® HSS T3 1.8 *μ*m, 2.1 × 100 mm). Chromatographic separation was achieved using gradient elution, starting from 20% acetonitrile (acetonitrile/formic acid, 1000 : 1 v/v) with water (water/formic acid, 1000 : 1 v/v) continuing linearly to 30% acetonitrile in 1 min, which was followed by a 6-min increase to 50% and further 3-min linear increase to 70%. The gradient was then shifted by a rise to 100% acetonitrile with a total acquisition runtime of 15 min. A sample volume of 4 *μ*L was injected and introduced to the column with a solvent flow rate of 0.3 mL·min^−1^. The column temperature was set at 35°C. The Q-TOF-MS system with an ESI source was performed in positive ion mode. Ion spray voltage floating was set at 5500 V. The mass range was set at *m/z* 100–1000 Da. The ESI heater temperature was set at 500°C. Nebulizer gas, auxiliary gas, and curtain gas were set at 50, 50, and 35 psi. Declustering potential and collision energy were 100 and 10 V. MS/MS ion data were acquired using an information-dependent acquisition mode. The accumulation time was set at 0.10 s, and the maximum number of candidate ions to monitor per cycle was kept at 15. MS conditions were corrected by APCI positive solution for the AB SCIEX TripleTOF^TM^ system. During the analysis period, calibration was carried out every five injections.

### 2.4. Data Analysis Strategy

In this study, PeakView software (version 2.2; AB SCIEX) was used for structural identification of diterpenoids. The peaks of candidate compounds were obtained by extract ions using dialog (XIC). The error ranges of these compounds were calculated using mass calculators. Two means including accurate-target and extensive-target were combined for comprehensive screening of the diterpenoids from *E. fischeriana*. For the accurate-target method, a database of target diterpenoids of *E. fischeriana*, including names, molecular formulas, and chemical structures was established by searching the relevant reported literature. Then, the compounds were identified by comparing the detail information with those of the reference substances and target diterpenoids. For the extensive-target method, the structures of diterpenoids were identified by comparing their accurate molecular weights and characteristic fragmentation behaviors with identified compounds. What's more, GNPS was used to screen more diterpenoids.

### 2.5. Molecular Networking

Molecular networking of the components from 70% ethanol fraction was created using the online workflow at GNPS (https://gnps.ucsd.edu). The MS/MS spectra window was filtered by choosing only the top six peaks in the ±50 *m/z* units. The data were then clustered using MS-cluster with a precursor ion mass tolerance of 2.0 Da and an MS/MS product ion tolerance of 0.5 Da to create consensus spectra. Consensus spectra that contained fewer than two spectra were discarded. A network was then created, where edges were filtered to have a cosine score of above 0.7 and more than 4 matched peaks. Further edges between two nodes were retained in the network only if each of the nodes appeared in each other's respective top 10 most similar nodes. The molecular network was visualized using Cytoscape software.

### 2.6. In Silico ADME Profiling of Diterpenoids

In silico absorption, distribution, metabolism, and excretion (ADME) processes are routinely chemoinformatics computer programs, which provide important data on whether a chemical compound can be applied as a medicine without conducting experimental studies. In this study, a free online web server (https://www.swissadme.ch) was used to predict the pharmacological properties of diterpenoids from *E. fischeriana*.

### 2.7. Targets Prediction for Diterpenoids

The structures of diterpenoids from *E. fischeriana* were drawn using ChemBioDraw Ultra 14.0. The structures of the compounds were saved as “mol2.” format and processed by the function of MM2 to optimize the energy of 3D molecular structures by ChemBio3D Ultra 14.0. The 3D molecular structure files of the diterpenoids were imported into PharmMapper [10] (https://lilab.ecust.edu.cn/pharmmapper/), which is an online server that utilizes the pharmacophore mapping approach for identification of potential drug targets. In this study, 50 targets of each compound obtained from PharmMapper were selected as potential targets.

### 2.8. Targets Prediction for Breast Cancer

The genes associated with targets of breast cancer were collected from GeneCards [11] (https://www.genecards.org/). GeneCards is an integrative, searchable database that provides user-friendly, comprehensive information on all annotated and predicted human genes. The platform with the keyword “breast cancer” was searched. The optimal cutoff values of scores were selected as 20 in this study.

### 2.9. Protein-Protein Interactions (PPIs)

STRING is a database (https://string-db.org/, ver. 11.0), which can be used to predict protein-protein interactions [12]. The interactions include physical and functional associations. They stem from computational prediction, knowledge transfer between organisms, and interactions aggregated from other databases. In this study, the data of PPIs were obtained from the STRING database. The species were limited to “Homo sapiens.” PPIs with comprehensive scores >0.7 were reserved.

### 2.10. Gene Ontology (GO) Term and Kyoto Encyclopedia of Genes and Genomes (KEGG) Pathway Enrichment Analysis

GO is a database for unification of biology. It can be classified into three categories: biological processes (BP), molecular functions (MF), and cellular components (CC) [13]. KEGG is a knowledge database, which helps researchers to classify the selected gene sets into their respective signaling pathways [14]. In this study, KOBAS was applied to do GO and KEGG pathway enrichment analysis. The *p*-value was corrected by the method introduced by Benjamini and Hochberg [15]. It controlled the false discovery rate, which was the expected percentage of rejected assumptions. In this study, enriched GO terms and pathways with *p*-value < 0.01 were selected. The horizontal bar of GO enrichment and bubble chart of KEGG pathway enrichment were plotted by bioinformatic tools from a free online data analysis platform (https://www.bioinformatics.com.cn/).

### 2.11. Network Construction

To further explore the multilevel mechanisms of diterpenoids from *E. fischeriana* in breast cancer therapy, five types of networks were constructed. First, a compounds-compound targets network was built by linking active compounds and corresponding targets. Then, a PPI network was established by connecting overlapping targets between compound targets and breast cancer targets. Next, a compounds-breast cancer targets-KEGG pathway network was built by connecting active compounds, overlapping targets and top 20 KEGG pathways. Last, a compounds-KEGG pathways-related targets network was structured by connecting active compounds and top 20 KEGG pathways-related targets. In network interactions, compounds and targets were described by node, and the interactions were encoded by edges. Network visualization software Cytoscape [16], which was well suited for visualizing molecular interactions in networks, is used to show all the above networks. Besides, the tool of NetworkAnalyzer [17] provides a powerful set of data integration, analysis, and visualization capabilities for analyzing complex networks. Meanwhile, cytoHubba [18], a free plug-in in Cytoscape, was utilized for retrieving information about hub genes and compounds.

### 2.12. Molecular Docking

Molecular docking is often used to estimate the binding affinities between candidate drugs and targets, as well as to predict the binding sites and binding postures of molecules. In this study, Surflex-Dock plug-in of Sybyl-X (version 2.0; TRIPOS Inc.) was used to perform molecular docking. The protein molecular structures were obtained from the PDB database (https://www.rcsb.org/). The visualization of intermolecular forces between the candidate compounds and their potential targets were performed on Discovery Studio 2020 program.

## 3. Results and Discussion

### 3.1. Diterpenoids from *E. fischeriana*

**Figure 2:**
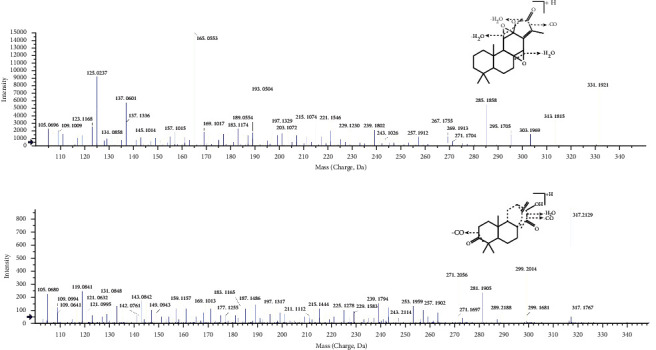
MS/MS spectrograms of jolkinolide B and ent-13*α*-hydroxyatis-16-ene-3,14-dione.

Dried *E. fischeriana* powders were soaked and extracted with methanol by using the ultrasound method. The extract was further applied to a D-101 macroporous resin column. The accurate-target method was used to test the numbers of diterpenoids in different fractions. It indicated that 70% ethanol fraction had the largest number of diterpenoids. The XIC spectrograms of identified compounds are shown in Figure S1. To get the information about precursor ions and characteristic fragment ions of the compounds, 5 available standards including jolkinolide A, jolkinolide B, 17-hydroxyjolkinolide A and 17-hydroxyjolkinolide B, and ent-13*α*-hydroxyatis-16-ene-3,14-dione were injected into the LC-MS system. The main fragmentation patterns of jolkinolide B and ent-13*α*-hydroxyatis-16-ene-3,14-dione were discussed in detail. The MS/MS spectrograms are displayed in Figure 2. Jolkinolide B produced a precursor ion [M + H]^+^ at *m/z* 331.1903 (C_20_H_26_O_4_) with the retention time of 12.86 min. The ion at *m/z* 313.2 was attributed to eliminate one molecule of water. The ion at *m/z* 303.2 was produced by further loss of one molecule of carbonyl. The fragment ions at *m/z* 295.2, 285.2, and 267.2 were attributed to losses of molecules of water. A series of characteristic product ions were observed in the MS/MS spectrogram, which were due to the cleavages of four rings. Among them, fragment ions at *m/z* 193.1, 165.1, 125.1, and 137.1 showed higher intensity than others, which were formed by the cleavages of ring B and ring C. Ent-13*α*-hydroxyatis-16-ene-3,14-dione produced [M + H]^+^ ion at *m/z* 317.2096 (C_20_H_28_O_3_) with the retention time of 8.11 min. The fragment ions at *m/z* 299.2, 281.2, and 263.2 were attributed to successive losses of three molecules of water. The fragment ions at *m/z* 289.2, 271.2, and 253.2 were attributed to successively eliminate one molecule of carbonyl and two molecules of water. The fragment ion at *m/z* 243.2 was produced by further loss of one molecule of carbonyl from 271.2. A set of characteristic product ions were attributed to skeleton residues, which were obtained by the cleavage of rings. Then, diterpenoids from 70% ethanol fraction were identified by comparing formulas and fragmentation patterns with accurate target diterpenoids searching from the literature [4, 19–44].

**Figure 3:**
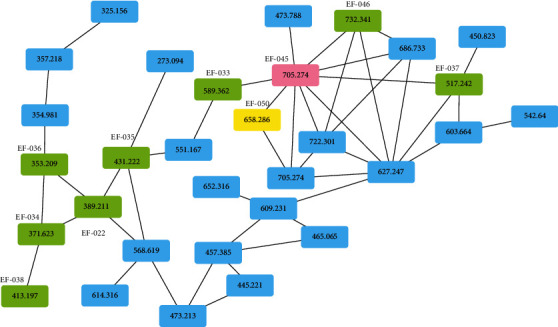
Partial nodes of cluster I.

GNPS allows rapid comparison using MS profiles of complex extracts. Molecular networking of *E. fischeriana* extract based on the MS/MS spectral similarity was generated by GNPS, which led to the presence of precursor ions visualized as nodes in the molecular map. First, diterpenoids were screened out from the clusters by their mass-to-charge ratios and molecular formulas. Then, the structures of unknown compounds were analyzed by comparing with the identified components, which were presented in the same cluster. With the aid of GNPS, diterpenoids with low content may be screened out. In this study, 10 diterpenoids were identified in cluster I (Figure 3). There was one pink node, which was detected in the retention time of 5.2 minutes, gave a protonated molecule [M + H]^+^ at *m/z* 705.3 (C_35_H_44_O_15_). It was identified as prostratin 20-O-(2′-galloyl)-*β*-D-glucopyranoside (EF-045). In the MS/MS spectrogram, the [M + H]^+^ ion could generate ions at *m/z* 687.3, 669.3, and 651.3 by neutral losses of molecules of water. What's more, it readily eliminated one glucosyl and one galloyl to produce the degradation ion at *m/z* 373.2. The product ions at *m/z* 355.2, 295.2, and 277.2 were attributed to residues by losses of H_2_O, CH_3_COOH, and H_2_O from ion at *m/z* 373.2. The ion at *m/z* 267.2 was obtained by further loss of CO from ion at *m/z* 295.2. A series of product ions were also observed by cleavage of the rings. Then, the nodes that were colored green were identified as EF-022, EF-033, EF-034, EF-035, EF-036, EF-037, EF-038, and EF-046 according to their fragmentation patterns by comparing with EF-045. The yellow node was detected at the retention time of 6.0 min and gave a protonated molecule [M + H]^+^ ion at *m/z* 658.3 (C_34_H_43_NO_12_), which was identified as a diterpenoid with nitrogen. The accurate-target method was used to screen the potential type of this compound. It indicated that it was a premyrsinane diterpene, which had similar skeleton as EF-045. In the MS/MS spectrogram, the fragment ions at *m/z* 640.3 and 622.3 were produced by neutral loss of two molecules of water. The high-abundance ions at *m/z* 313.2, 295.2, 277.2, and 267.2 were attributed to neutral losses of CH_3_COOH, nicotinoyl, H_2_O, and CO from the precursor ion at *m/z* 658.3. Finally, it was tentatively identified as (1aS,3S,3aR,4R,4aR,5R,6S,7aR,9R,9aR,9bS)-7a-hydroxy-1,1,6,9-tetramethyl-3a-((nicotinoyloxy)methyl)-8-oxotetradecahydro-1H-cyclopropa[3,4]benzo[1,2-f]azulene-3,4,5,9-tetrayl tetraacetate (EF-050). The analytical method has some limitations in identifying the isomers for the complex structures of diterpenoids. To confirm the structures of these diterpenoids, NMR experiments are necessary.

**Figure 4:**
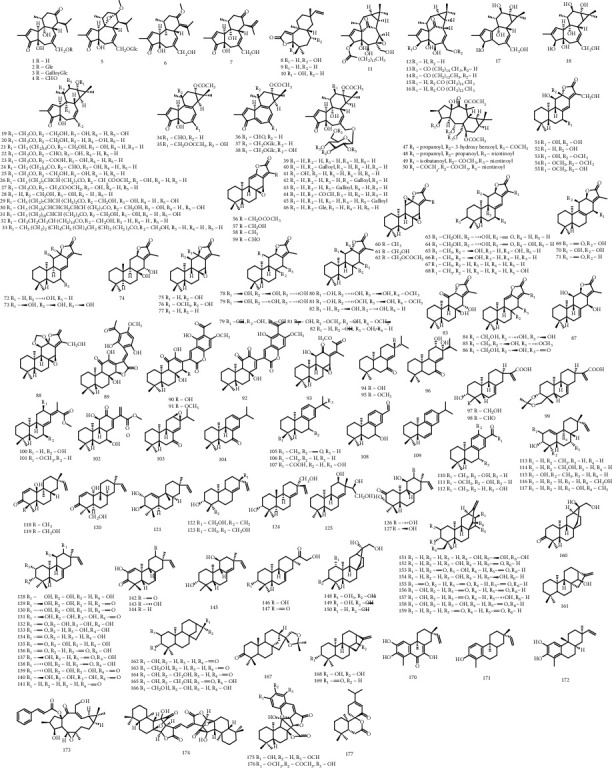
The structures of diterpenoids.

In this study, positive and negative ion modes were both performed. There were more diagnostic fragment ions in the positive ion mode, which were helpful to analyze the structures of diterpenoids. The supposed ions of the candidate compounds simulated by MasterView software were compared with the ions of MS/MS data, which would raise the reliability of the results. In view of compound cracking rules, neutral losses like H_2_O, CO, HCOOH, CH_3_OH, CH_3_COOH, and cleavages of rings A, B, C, and D were responsible for the main fragmentation patterns of diterpenoids. What's more, it showed that diterpene lactones were liable to crack the lactonic rings. Fatty chains and sugar residues were likely to lose when they were attached to diterpenoids. At last, a total of 144 diterpenoids were identified by UHPLC-Q-TOF-MS and GNPS. Among these diterpenoids, 5 compounds were definitely identified by comparing with reference substances, 129 compounds were identified according to the literature, and 10 compounds were tentatively identified according to the data of GNPS. After referring to the literature, another 33 diterpenoids from *E. fischeriana*, which were not detected in the MS data, were searched out. Concerning the carbon skeletons and substituents at specific positions, these 177 diterpenoids (Figure 4) were classified into 13 subtypes, namely, daphnane diterpene, diterpenoid lactone, ingenane diterpene, tigliane diterpene, premyrsinane diterpene, ent-abietane diterpene, rosane diterpene, piramane diterpene, ent-atisane diterpene, ent-kaurane diterpene, norrostane diterpene, lathyrane diterpene, and dimeric diterpene. Among them, tigliane diterpene, ent-abietane diterpene, piramane diterpene, and ent-atisane diterpene accounted for larger proportions than others. The information of the 177 diterpenoids is shown in Table S1.

Lipinski's rule of five is a rule of thumb to evaluate if a compound with certain pharmacological or biological activities could be a likely orally active drug in humans, which includes a molecular mass less than 500 Da, no more than 5 H-bond donors, no more than 10 H-bond acceptors, and partition coefficient logP not greater than 5. The results predicted by the online web server (https://www.swissadme.ch) are shown in Table 1. It indicated that 130 of 177 diterpenoids completely adjusted to Lipinski's rule. The brain or intestinal estimated permeation BOILED-Egg method is an accurate predictive model that works by calculating the polarity and lipophilicity of small molecules. This prediction provides a visual clue to the compounds of the oral absorption potential of drug candidates. In this study, GI absorption of each compound was predicted. The results showed that 142 of 177 diterpenoids had high GI absorption.

### 3.2. Compound-Compound Target Network Analysis

**Figure 5:**
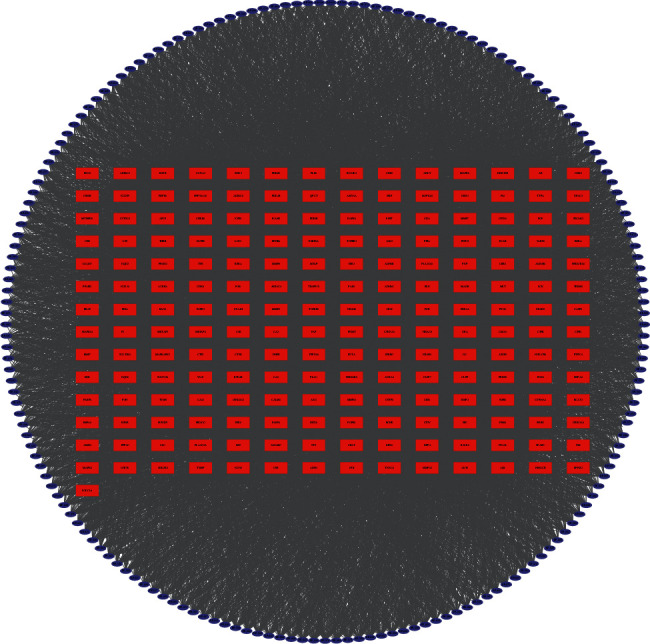
Compound-compound target network.

The compound-compound target network is depicted in Figure 5, including 374 nodes (177 active compound nodes and 197 compound target nodes) and 8455 edges. In this network, the rectangles represented the targets, and the ovals represented the compounds. It was found that some targets were hit by multiple compounds. The average number of targets per component is 47.8, and the mean degree of components per target is 42.9. It clearly showed that *E. fischeriana* fit the multicomponent and multitarget characteristics of traditional Chinese medicine. Consequently, an approximate observation of the relationship between bioactive compounds and compound targets was obtained.

### 3.3. Target Acquisition for Breast Cancer and PPI Network Construction

**Figure 6:**
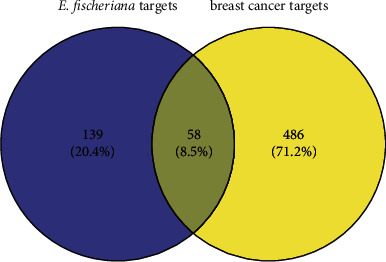
Venn diagram of the target genes for breast cancer and *E. fischeriana*.

**Figure 7:**
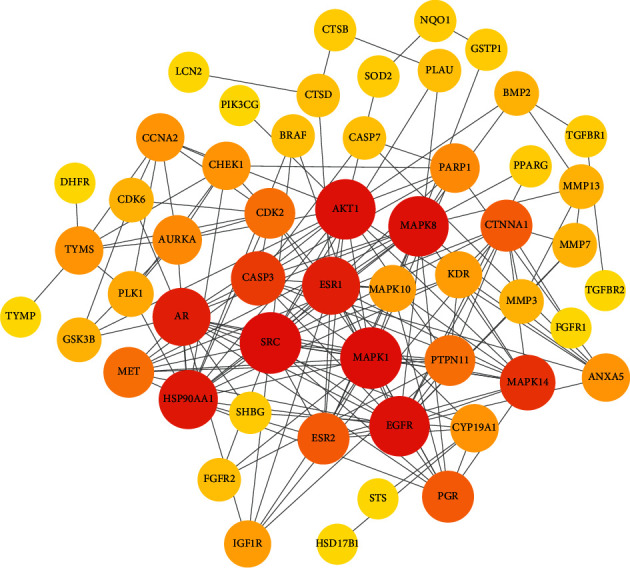
PPI network of the 58 overlapping target genes (the darker and larger the node, the more proteins it interacts with).

There were 544 target genes that could correspond to breast cancer, which were identified from GeneCards. After the intersection process, it was found that there were 58 overlapping target genes between breast cancer targets and compound targets. A Venn diagram of the target genes for breast cancer and *E. fischeriana* compounds is displayed in Figure 6. There were 53 nodes and 175 edges in the PPI network (Figure 7), which meant these targets might be the key targets for *E. fischeriana* treating breast cancer.

### 3.4. GO Enrichment Analysis

**Figure 8:**
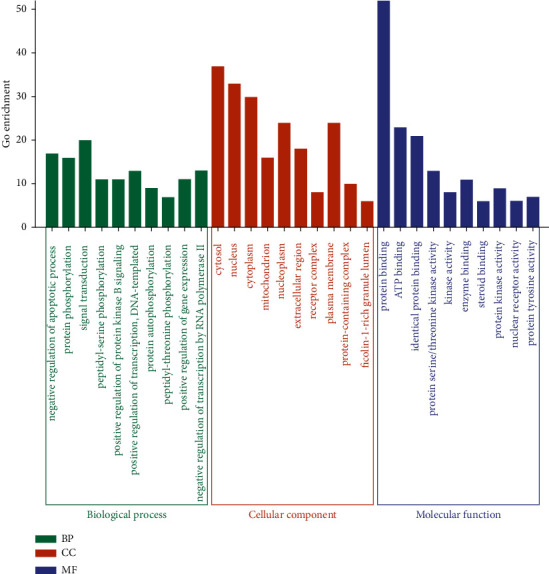
GO enrichment analysis for 58 overlapping targets.

After GO enrichment analysis of 58 overlapping targets, a total of 438 GO entries were found with the corrected *p*-value < 0.01.  Figure 8 lists the top 10 entries of each category, namely BP, CC, and MF. The most significantly enriched terms were significantly associated to the regulation of apoptotic process, cytosol, and protein binding in the three categories, respectively.

### 3.5. KEGG Pathway Analysis

**Figure 9:**
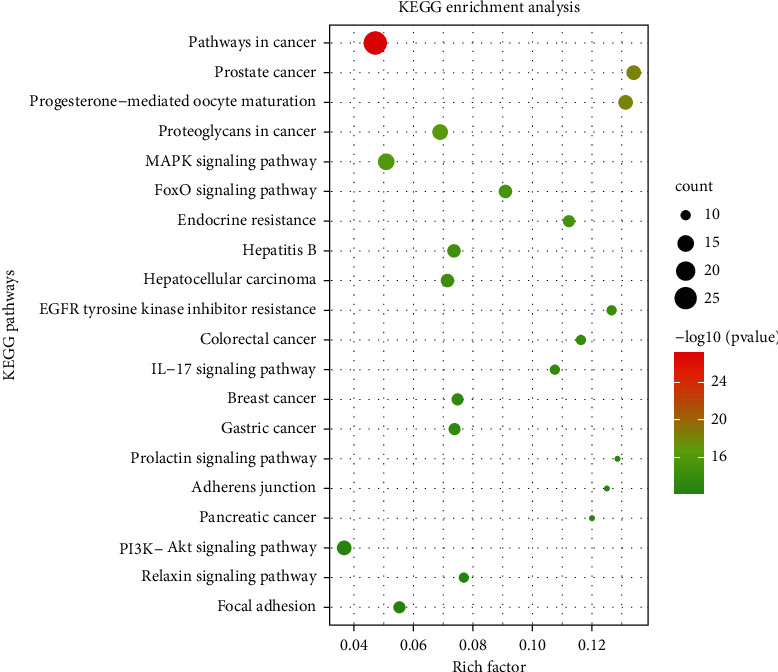
KEGG enrichment analysis for 58 overlapping targets.

The 58 overlapping targets were further mapped to 134 pathways with *p* < 0.01. The top 20 KEGG pathways were shown in Figure 9. The 20 pathways belonged to four categories: human diseases (11/20), organismal systems (4/20), environmental information processing (3/20), and cellular processes (2/20). It showed that *E. fischeriana* integrated multiple signaling pathways to the cancer, endocrine system, immune system, and signal transduction. Based on the results of pathway analysis, it was found that these high-degree pathways were closely related to breast cancer. The main signaling pathways were discussed below.

IL-17 signaling pathway is a typical inflammation pathway that is closely related to the generation of inflammatory responses. Inflammation is a response against pathogens, allergens, and chemical and physical damages, which manifests itself in two types including acute inflammation and chronic inflammation. Acute inflammation leads to tissue repair, while chronic inflammation develops to various types of cancers, metabolic disorders, and autoimmune diseases. IL-17 is produced through several mechanisms including population growth and upregulation expression of genes. It is involved in the development of inflammation such as NF-*κ*B activation [45]. It has been reported that the extracts from *Euphorbia* species may alter the expression of IL-17 [46–48].

MAPK signaling pathway is the core of many signaling pathways and plays a key role in many cell proliferation-related signaling pathways. It is an important type of molecules that carry the signals converted and transmitted by the receiving membrane receptors into the nucleus of the cell [49, 50]. Tumor metastasis is one of the main causes of mortality in cancer patients. Cell adhesion to the extracellular matrix is crucial in cancer progression and metastasis. Sun et al. [51] studied the antiadhesion and anti-invasion effects of jolkinolide B, a diterpenoid compound from *E. fischeriana*. It showed that jolkinolide B possessed antimetastasis activity and influenced cell-ECM adhesion through suppression of *β*1-integrin expression and phosphorylation of FAK in human breast cancer MDA-MB-231 cells. The MAPK signaling pathway may play a critical role in these effects.

PI3K-Akt signaling pathway is one of the most important intracellular signaling pathways, which is associated with numerous aspects of cellular functions. These functions play vital roles in survival, quiescence, and growth in normal physiological circumstances as well as a variety of pathological disorders, including cancers [52]. Ma et al. [53] found that 12-deoxyphorbol-13-palmitate, a tetracyclic diterpene monomer compound from *E. fischeriana*, could inhibit the proliferation of leukemia cells *in vivo and in vitro* and induce the apoptosis of leukemia cells, which might be a result of suppressing the PI3K-Akt signaling pathway.

Prolactin is a secretory cytokine produced by various tissues. Binding to the cognate prolactin receptor, it activates intracellular signaling via JAK, ERK, and STAT proteins. Prolactin regulates diverse activities in normal and abnormal conditions, such as malignancies [54, 55]. Evidence in animals suggested that an extract from *Euphorbia* increases serum prolactin [56]. So far, there is little literature reported regarding if the prolactin signaling pathway participates in treatment of breast cancer by *E. fischeriana*. However, it showed that *Euphorbia* species may inhibit the growth of tumor cells by JAK-STAT [57, 58] or MAPK-ERK signaling pathways [59].

### 3.6. Compounds-Breast Cancer Targets-KEGG Pathways Network Construction and Analysis

**Figure 10:**
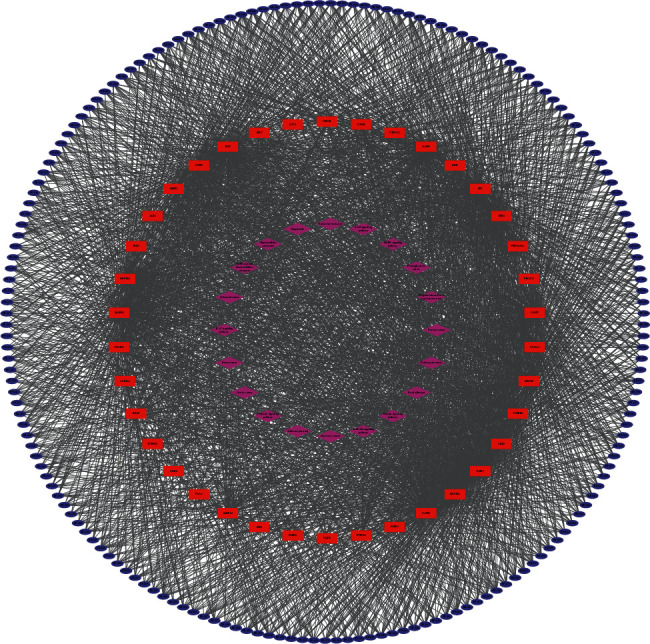
Compound-breast cancer target-KEGG pathway network.

**Figure 11:**
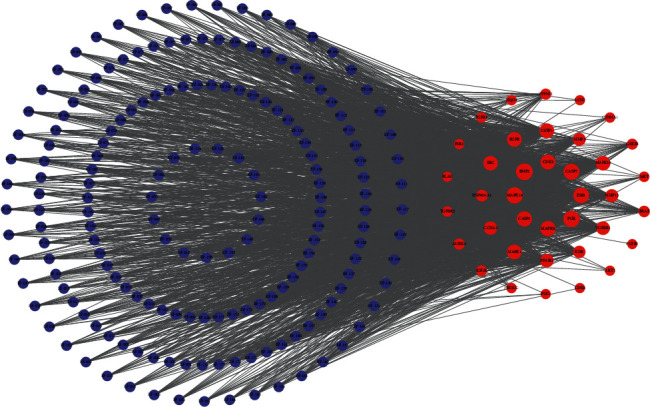
Compound-KEGG pathway-related target network (blue circles represent compounds of *E fischeriana*; red circles represent compound/breast cancer targets. The closer to the center, the greater the degree).

**Figure 12:**
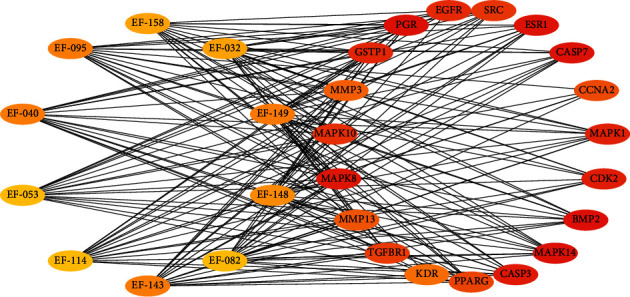
Top 10 compounds and top 19 targets.

The compounds-breast cancer targets-KEGG pathways network was structured by connecting active compounds, top 20 KEGG pathways, and targets related to KEGG pathways (Figure 10). The compounds-KEGG pathways-related targets network was structured by connecting active compounds and top 20 KEGG pathways-related targets (Figure 11). Top 10 compounds and top 19 targets were identified by the tool of cytoHubba (Figure 12).

The top 10 compounds were EF-032, EF-040, EF-053, EF-082, EF-095, EF-114, EF-143, EF-148, EF-149, and EF-158. It suggested that these compounds might play important roles in the treatment of breast cancer. Among these compounds, they were 3 ent-abietane diterpenes, 3 ent-atisane diterpenes, 2 tigliane diterpenes, 1 rosane diterpene, and 1 piramane diterpene. It concluded that *E. fischeriana* treated breast cancer not only by the main components but also by the microconstituents, which reflected the overall regulatory role of multicomponent treating breast cancer.

The top 19 genes were epidermal growth factor receptor (EGFR), estrogen receptor (ESR1), mitogen-activated protein kinase 1 (MAPK1), mitogen-activated protein kinase 10 (MAPK10), mitogen-activated protein kinase 14 (MAPK14), mitogen-activated protein kinase 8 (MAPK8), progesterone receptor (PGR), collagenase 3 (MMP13), stromelysin-1 (MMP3), peroxisome proliferator-activated receptor gamma (PPARG), proto-oncogene tyrosine-protein kinase Src (SRC), bone morphogenetic protein 2 (BMP2), caspase-3 (CASP3), caspase-7 (CASP7), cyclin-A2 (CCNA2), cell division protein kinase 2 (CDK2), glutathione S-transferase P (GSTP1), vascular endothelial growth factor receptor 2 (KDR), and TGF-beta receptor type-1 (TGFBR1). The key targets were discussed below.

Estrogen receptor is a hormone receptor, which is involved in the development and maintenance of the female reproductive system. It is subcategorized into two types: ESR1 and ESR2. In 65% of breast cancer, ESR1 is found to be the main culprit. It expresses in mammary glands and is responsible for initiating many signaling pathways that lead to differentiation and development of breast tissue [60]. Estrogens can activate the MAPK pathways through SRC. MAPK may increase the phosphorylation of cyclins and promote the progression through cell cycle. The MAPK pathways interact with the phosphorylation level and states of ER and PGR [61]. Progestin can activate c-SRC and enhance prolactin-mediated activation of STAT through MAPK pathways to promote cell proliferation [62, 63]. EGFR is an important target for the management of breast cancer. C-SRC is a nonreceptor tyrosine kinase protein that interacts with cell surface growth factor receptors and the intracellular signaling pathway, which promote tumorigenesis and metastatic progression. EGFR and c-SRC are overexpressed in approximately 70% of breast cancer cases. c-SRC-mediated EGFR phosphorylation is critical for receptor function and breast cancer cell survival [64].

Up to now, diterpenoids from *E. fischeriana* have been reported to act on MAPK. However, there were few reports about *E. fischeriana* targeting at ER, PGR, EGFR, Src, and so on. It suggested that the predicted results can serve as references for researchers studying potential targets of *E. fischeriana* on breast cancer in the future. Moreover, these predicted genes were involved in cell proliferation, gene transcription, apoptosis, signal transduction, DNA damage and repair, tumor differentiation, metastasis, and cell cycle, which indicated that *E. fischeriana* can treat breast cancer comprehensively.

### 3.7. Molecular Docking

To estimate the binding affinities, molecular docking simulations between 177 diterpenoids and top 19 targets were carried out by Surflex-Dock. The PDB codes of these targets were obtained from the results of PharmMapper. The scores are shown in Table S2. The visualization of intermolecular forces between top 19 targets and compounds that had the most score of each target are displayed in Figure S2. The detail interactions between EF-030 and CASP3 protein were taken as example to show the binding behaviors. The docking pose of EF-030 showed three conventional hydrogen bond interaction bindings with Thr62, Glu167, and Leu167 and a series of Pi-alkyl and alkyl interaction bindings with His121, Tyr204, Phe256, and Ala254.

## 4. Conclusion

The structures of diterpenoids were identified by the integrated strategy of UHPLC-Q-TOF-MS and GNPS. The fragmentation patterns of diterpenoids were discussed. A total of 177 diterpenoids with 13 types were collected in this article by accurate-target and extensive-target methods. Anti-breast-cancer mechanisms were predicted by the network pharmacological method. There were 58 overlapping target genes between 197 compound-related targets and 544 breast cancer-related targets. It was found by GO analysis that they were closely related to regulation of apoptotic process, cytosol, and protein binding in biological processes, cellular component, and molecular functions. Based on the results of KEGG pathway analysis, it was found that these high-degree pathways were closely related to the breast cancer. The treatment effect of *E. fischeriana* on breast cancer might be performed through signaling pathways, such as IL-17 signaling pathway, MAPK signaling pathway, and PI3K-Akt signaling pathway. In summary, this is the first one that combines diterpenoids identification, target prediction, network analysis, and gene enrichment analysis by a network pharmacology method to elucidate the molecular and pharmacological mechanism of *E. fischeriana* against breast cancer from a systematic perspective. In future, more experiments should be implemented to verify the validity of the findings in further pharmacological and molecular research.

## Figures and Tables

**Table 1:** Main ADME profiling of diterpenoids.

No.	Lipinski #violations	iLOGP	XLOGP3	WLOGP	MLOGP	#H-bond acceptors	#H-bond donors	GI absorption
EF-001	0	2.48	0.40	1.41	1.08	5	3	High
EF-002	2	2.37	−1.20	−0.76	−1.19	10	6	Low
EF-003	3	3.14	−0.58	0.22	−1.29	14	8	Low
EF-004	0	2.83	0.97	1.98	1.43	6	2	High
EF-005	1	2.76	−0.15	0.05	−0.49	10	5	Low
EF-006	0	2.42	0.52	1.48	0.99	5	3	High
EF-007	0	2.20	0.50	1.33	0.99	5	3	High
EF-008	0	3.08	4.02	3.63	3.43	3	1	High
EF-009	1	3.26	5.36	4.66	4.31	2	0	High
EF-010	0	3.12	4.60	3.98	3.84	3	1	High
EF-011	0	3.12	4.60	5.67	3.84	7	3	High
EF-012	0	2.33	0.21	0.82	1.17	5	4	High
EF-013	2	6.91	8.11	6.85	4.25	6	3	Low
EF-014	1	5.31	7.03	6.07	3.89	6	3	Low
EF-015	2	5.75	7.56	6.85	4.25	6	3	Low
EF-016	1	5.05	6.48	6.07	3.89	6	3	Low
EF-017	0	2.54	−1.30	0.67	1.17	5	5	High
EF-018	1	2.29	−1.64	−0.28	0.45	6	6	High
EF-019	0	2.70	−0.26	0.52	0.59	7	4	High
EF-020	0	3.47	0.54	1.54	1.39	6	3	High
EF-021	2	6.14	8.03	6.99	4.25	6	3	Low
EF-022	0	2.83	1.22	1.74	1.43	6	2	High
EF-023	0	2.82	1.28	1.62	1.44	7	3	High
EF-024	2	5.69	8.55	7.2	4.16	6	2	Low
EF-025	0	2.85	0.70	1.53	1.52	6	3	High
EF-026	2	6.96	8.76	8.12	4.79	7	2	Low
EF-027	0	3.57	1.28	2.10	1.87	7	2	High
EF-028	0	2.62	0.13	0.96	1.17	5	4	High
EF-029	2	5.99	7.10	6.77	4.16	6	3	Low
EF-030	2	6.60	7.50	7.32	4.43	6	3	Low
EF-031	2	6.07	8.18	7.55	4.51	6	3	Low
EF-032	2	5.81	8.26	7.34	4.88	5	2	Low
EF-033	2	4.94	7.17	7.54	5.01	5	2	Low
EF-034	0	2.99	1.42	2.69	2.16	5	1	High
EF-035	0	3.40	0.22	2.17	1.79	7	2	High
EF-036	0	3.08	2.56	3.49	2.91	4	0	High
EF-037	1	2.98	0.28	1.11	0.63	9	4	Low
EF-038	1	2.26	−0.97	0.22	−0.14	10	5	Low
EF-039	3	2.77	−0.89	−0.65	−0.74	11	6	Low
EF-040	3	3.13	−0.27	0.34	−0.85	15	8	Low
EF-041	3	3.06	−1.86	−1.67	−1.49	12	7	Low
EF-042	3	3.56	0.28	0.34	−0.85	15	8	Low
EF-043	3	2.35	−0.27	0.34	−0.85	15	8	Low
EF-044	2	3.46	−0.87	−0.07	−0.38	12	5	Low
EF-045	3	2.77	0.28	0.34	−0.85	15	8	Low
EF-046	3	3.24	−3.03	−2.82	−2.91	16	9	Low
EF-047	2	3.24	3.59	3.58	2.17	12	2	Low
EF-048	2	3.71	3.35	3.66	1.90	12	1	Low
EF-049	2	0.99	2.88	3.27	1.72	12	1	Low
EF-050	2	3.13	2.67	2.60	1.60	13	1	Low
EF-051	0	2.31	1.04	1.03	1.57	6	4	High
EF-052	0	2.52	2.34	2.06	2.38	5	3	High
EF-053	0	2.65	1.58	1.69	1.79	6	3	High
EF-054	0	3.02	2.11	2.34	2.01	6	2	High
EF-055	0	2.85	1.58	1.69	1.79	6	3	High
EF-056	0	3.20	3.20	3.68	3.14	5	0	High
EF-057	0	2.98	3.17	3.11	2.81	4	1	High
EF-058	0	3.40	3.87	4.14	3.66	3	0	High
EF-059	0	2.64	2.44	2.74	2.81	4	2	High
EF-060	0	3.31	3.15	3.35	3.31	4	0	High
EF-061	0	3.16	2.45	2.32	2.48	5	1	High
EF-062	0	3.30	2.47	2.89	2.82	6	0	High
EF-063	0	2.03	1.26	1.92	1.89	5	2	High
EF-064	0	1.73	−0.04	0.89	1.07	6	3	High
EF-065	0	2.95	2.50	2.74	2.81	4	2	High
EF-066	0	3.21	3.80	3.77	3.66	3	1	High
EF-067	1	3.43	4.78	4.80	4.54	2	0	High
EF-068	0	3.11	3.51	3.92	3.66	3	1	High
EF-069	0	2.75	3.15	3.09	2.72	4	1	High
EF-070	0	2.75	3.15	3.09	2.72	4	1	High
EF-071	0	3.11	4.27	4.12	3.57	3	0	High
EF-072	0	3.20	3.80	3.77	3.66	3	1	High
EF-073	0	2.64	1.74	2.06	2.38	5	3	High
EF-074	0	3.04	2.90	2.97	2.90	4	2	High
EF-075	0	2.99	2.92	2.98	2.90	4	1	High
EF-076	0	3.20	2.69	2.96	2.70	5	1	High
EF-077	0	3.31	3.90	4.01	3.75	3	0	High
EF-078	0	2.89	1.93	1.94	2.07	5	3	High
EF-079	0	2.20	1.93	1.94	2.07	5	3	High
EF-080	0	2.57	1.00	0.88	1.09	7	4	High
EF-081	0	2.68	1.54	1.54	1.31	7	3	High
EF-082	0	3.09	2.90	2.97	2.90	4	2	High
EF-083	0	2.30	2.27	2.15	1.98	5	2	High
EF-084	0	3.10	2.18	2.06	1.98	5	3	High
EF-085	0	3.49	3.41	3.75	3.03	4	1	High
EF-086	0	2.62	2.98	2.27	1.89	5	2	High
EF-087	0	2.64	2.44	2.74	2.81	4	2	High
EF-088	0	2.87	2.40	2.30	2.30	6	1	High
EF-089	1	3.05	3.92	3.88	1.06	9	4	Low
EF-090	1	3.35	3.53	3.9	0.95	9	4	Low
EF-091	1	3.68	4.06	4.55	1.14	9	3	Low
EF-092	1	2.70	4.31	4.22	1.65	8	2	Low
EF-093	0	3.29	3.26	2.80	2.20	5	1	High
EF-094	0	3.18	4.63	4.52	3.82	2	1	High
EF-095	0	3.65	5.16	5.17	4.05	2	0	High
EF-096	0	3.13	3.65	3.49	2.94	3	2	High
EF-097	0	1.72	3.36	3.15	2.81	4	3	High
EF-098	0	1.69	3.21	3.36	2.72	4	2	High
EF-099	0	3.32	4.37	4.95	3.87	4	1	High
EF-100	0	3.76	4.32	4.41	3.88	3	1	High
EF-101	1	3.92	5.25	5.45	4.31	3	0	High
EF-102	0	3.02	3.47	2.42	2.11	5	2	High
EF-103	0	3.50	4.65	4.53	3.82	2	0	High
EF-104	1	3.52	5.34	5.32	4.65	1	0	High
EF-105	1	3.49	5.97	5.48	4.56	1	0	High
EF-106	1	3.85	7.07	5.84	6.64	0	0	Low
EF-107	0	2.55	3.38	3.9	3.57	3	2	High
EF-108	0	2.90	5.24	3.94	2.83	3	2	High
EF-109	1	3.79	6.85	5.81	6.55	0	0	Low
EF-110	0	3.22	5.82	4.63	3.68	2	1	High
EF-111	0	3.56	5.99	4.21	3.88	3	1	High
EF-112	0	3.00	4.33	4.09	3.41	2	1	High
EF-113	1	3.62	5.85	5.11	4.75	1	1	High
EF-114	0	3.44	5.18	4.08	3.82	2	2	High
EF-115	0	3.48	4.74	4.08	3.82	2	2	High
EF-116	0	3.38	4.44	4.08	3.82	2	2	High
EF-117	0	3.27	4.32	4.08	3.82	2	2	High
EF-118	0	3.19	4.68	4.29	3.73	2	1	High
EF-119	0	2.98	4.01	3.26	2.85	3	2	High
EF-120	0	2.74	4.01	3.26	2.85	3	2	High
EF-121	0	3.41	4.74	4.08	3.82	2	2	High
EF-122	0	3.35	4.09	4.08	3.82	2	2	High
EF-123	0	3.27	4.81	4.08	3.82	2	2	High
EF-124	0	3.24	4.09	4.08	3.82	2	2	High
EF-125	0	3.03	4.12	3.28	3.05	3	3	High
EF-126	0	2.59	2.94	2.31	2.09	4	3	High
EF-127	0	2.56	2.94	2.31	2.09	4	3	High
EF-128	0	2.89	2.93	3.05	2.94	3	3	High
EF-129	0	3.00	2.62	3.26	2.85	3	2	High
EF-130	0	3.09	2.62	3.26	2.85	3	2	High
EF-131	0	2.47	2.06	2.23	1.99	4	3	High
EF-132	0	2.53	2.08	2.23	1.99	4	3	High
EF-133	0	2.67	2.64	3.26	2.85	3	2	High
EF-134	0	3.15	3.61	4.29	3.73	2	1	High
EF-135	0	2.87	3.06	3.26	2.85	3	2	High
EF-136	0	2.52	2.64	3.47	2.76	3	1	High
EF-137	0	2.76	2.94	3.26	2.85	3	2	High
EF-138	0	2.70	2.94	3.26	2.85	3	2	High
EF-139	0	1.89	2.06	2.23	1.99	4	3	High
EF-140	0	2.47	2.06	2.23	1.99	4	3	High
EF-141	1	3.41	4.93	5.32	4.65	1	0	High
EF-142	0	2.83	3.33	4.16	2.67	3	1	High
EF-143	0	3.07	3.63	3.95	2.76	3	2	High
EF-144	0	3.03	3.68	3.95	2.76	3	2	High
EF-145	0	3.34	4.16	4.08	3.82	2	2	High
EF-146	0	2.85	3.02	3.49	2.94	3	2	High
EF-147	0	2.63	2.70	3.70	2.85	3	1	High
EF-148	0	2.51	2.38	2.08	2.34	4	4	High
EF-149	0	2.67	2.38	2.08	2.34	4	4	High
EF-150	0	2.73	3.36	3.11	3.19	3	3	High
EF-151	0	2.33	1.39	2.07	2.09	4	3	High
EF-152	0	3.04	3.53	4.13	3.82	2	1	High
EF-153	0	2.44	2.56	3.30	2.85	3	1	High
EF-154	0	0.00	3.85	3.92	3.93	2	2	High
EF-155	0	0.00	2.53	3.51	2.76	3	0	High
EF-156	0	2.60	2.48	3.30	2.85	3	1	High
EF-157	0	0.00	2.37	3.10	2.94	3	2	High
EF-158	0	2.80	2.80	3.10	2.94	3	2	High
EF-159	0	2.90	3.22	4.33	3.73	2	0	High
EF-160	0	2.61	3.04	3.32	3.05	3	2	High
EF-161	1	3.42	5.38	4.95	4.86	1	1	High
EF-162	0	2.86	3.91	3.96	3.7	2	1	High
EF-163	0	3.04	4.47	4.21	3.93	2	1	High
EF-164	0	2.88	3.04	3.32	3.05	3	2	High
EF-165	0	2.29	2.39	2.29	2.2	4	3	High
EF-166	0	2.82	3.36	3.11	3.19	3	3	High
EF-167	0	3.77	4.60	5.12	4.11	3	0	High
EF-168	0	3.22	3.85	3.92	3.93	2	2	High
EF-169	1	3.26	5.06	5.15	4.75	1	0	High
EF-170	0	2.82	4.19	4.05	2.04	4	3	High
EF-171	1	3.24	6.20	4.90	4.6	1	1	High
EF-172	0	3.17	5.85	4.60	3.94	2	2	High
EF-173	0	3.85	4.38	3.60	2.7	6	2	High
EF-174	0	3.85	4.38	4.49	2.7	10	2	High
EF-175	0	0.00	3.91	3.01	2.42	8	2	High
EF-176	1	2.98	4.14	3.22	1.95	9	2	High
EF-177	0	2.99	4.44	4.45	3.66	3	0	High

## Data Availability

Supplementary materials are available as Supporting Information and can be requested by sending e-mail to the corresponding author.

## Supplementary Materials

Supplementary Materials Figure S1: the XIC spectrograms of compounds identified by UHPLC-Q-TOF-MS. Figure S2: the visualization of intermolecular forces between targets and compounds. Table S1: the information of 177 diterpenoids. Table S2: the results of molecular docking.
