# Supplementary material for: Integrated Strategy of UHPLC-Q-TOF-MS and Molecular Networking for Identification of Diterpenoids from Euphorbia fischeriana Steud. and Prediction of the Anti-Breast-Cancer Mechanism by the Network Pharmacological Method
Source: Evid Based Complement Alternat Med. 2021 Nov 11;2021:3829434. doi: 10.1155/2021/3829434 (PMC8601799; doi:10.1155/2021/3829434)
Supplement: Supplementary Materials — Figure S1: the XIC spectrograms of compounds identified by UHPLC-Q-TOF-MS. Figure S2: the visualization of intermolecular forces between targets and compounds. Table S1: the information of 177 diterpenoids. Table S2: the results of molecular docking. [file 3829434.f1.docx]

**Supplementary Materials**

**Integrated strategy of** **UHPLC-Q-TOF-MS and** **molecular networking for identification of** **diterpenoids from *Euphorbia fischeriana* Steud. and** **prediction the** **anti-breast cancer mechanism by** **network pharmacological method**

MA Tian-Cheng^1,2^, MA Yu-Kun^1^, ZHANG Jin-Ling^1^, LIU Lei^1^, SUN Jia^1^, GUO Li-Na^1^, LIU Qi^1^, SUN Yu^1*^

*^1^Research Institute of Medicine and Pharmacy, Qiqihar Medical University, Bukui Road 333, Qiqihar 161006, Heilongjiang, China*

*^2^School of Traditional Chinese Materia Medica, Shenyang Pharmaceutical University, Wenhua Road 103, Shenyang 110016, Liaoning, China*

**Abstract**

Figure S1: The XIC spectrograms of compounds identified by UHPLC-Q-TOF-MS. The code listed in Table 1 were labeled in the spectrograms.

Figure S2: The visualization of intermolecular forces between top 19 targets and compounds which had the most score of each target.

Table S1: The information of 177 diterpenoids.

Table S2: The results of molecular docking by Surflex-Dock plug-in the Sybyl-X.


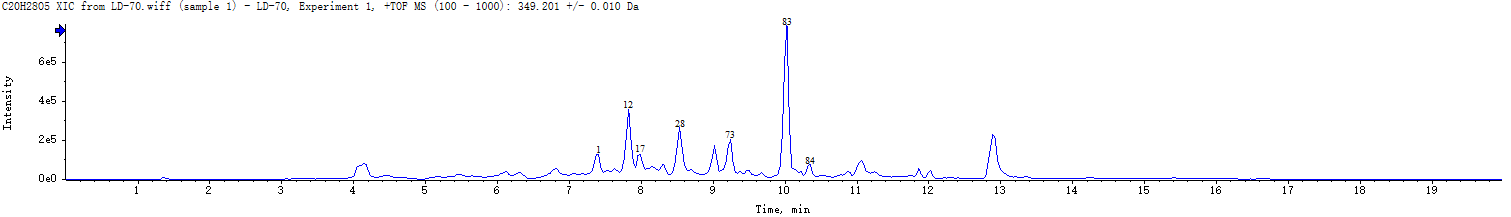


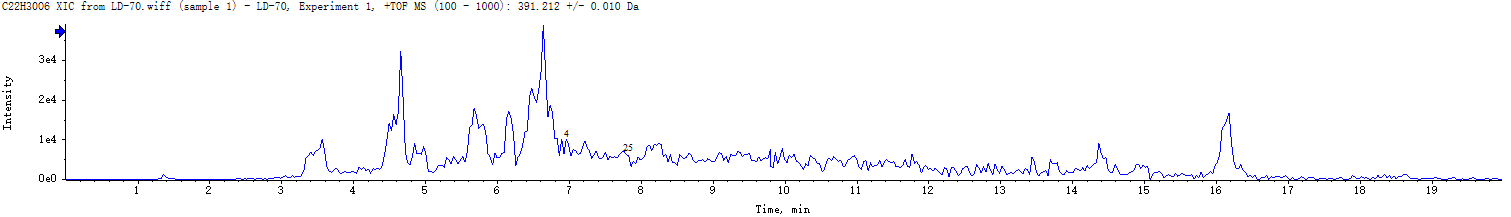


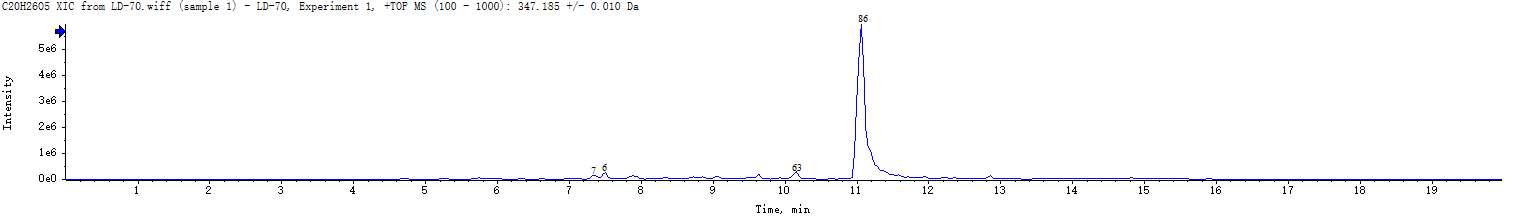


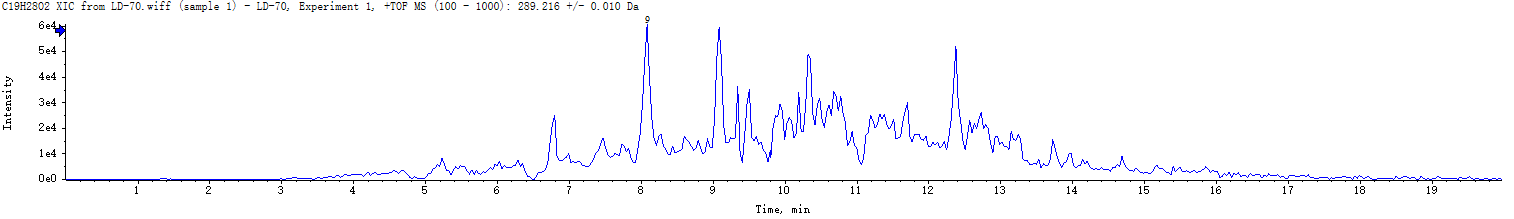


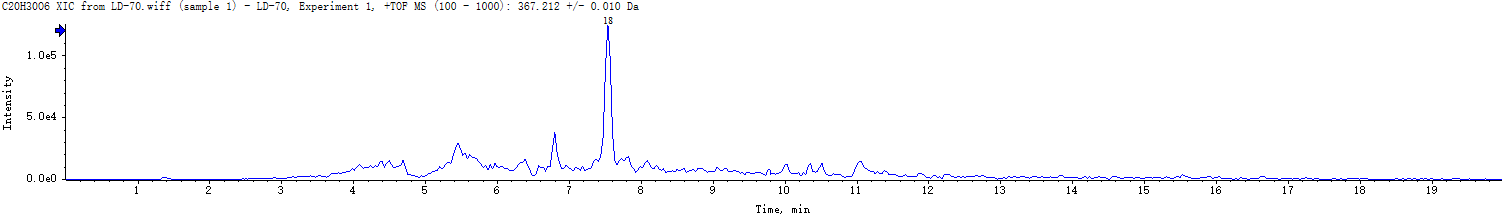


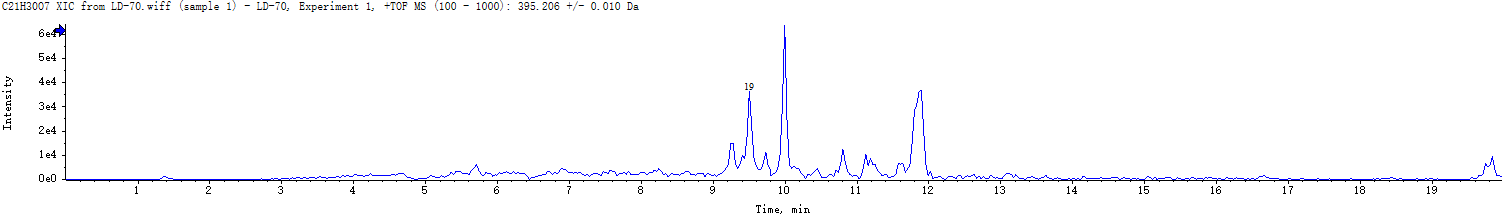


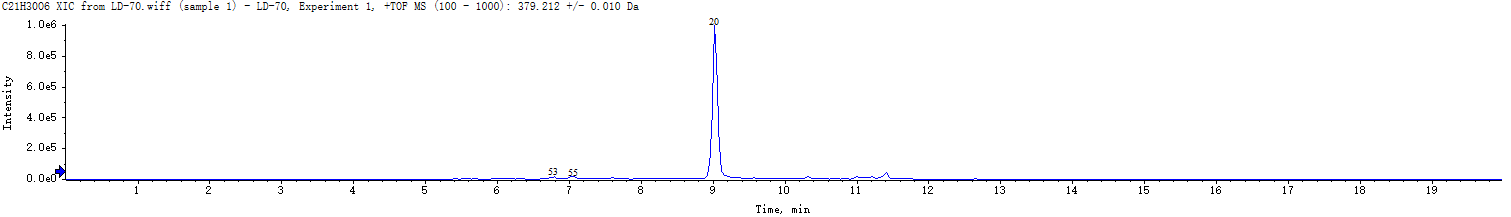


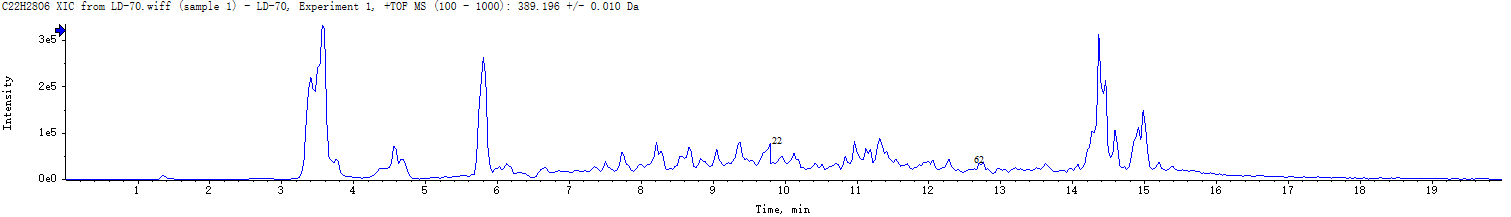


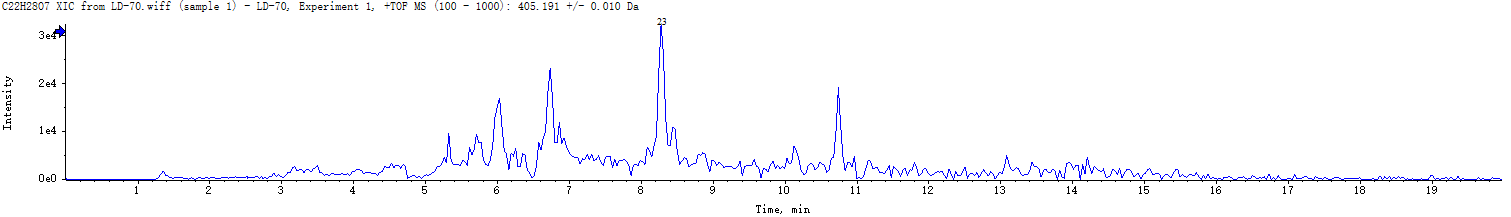


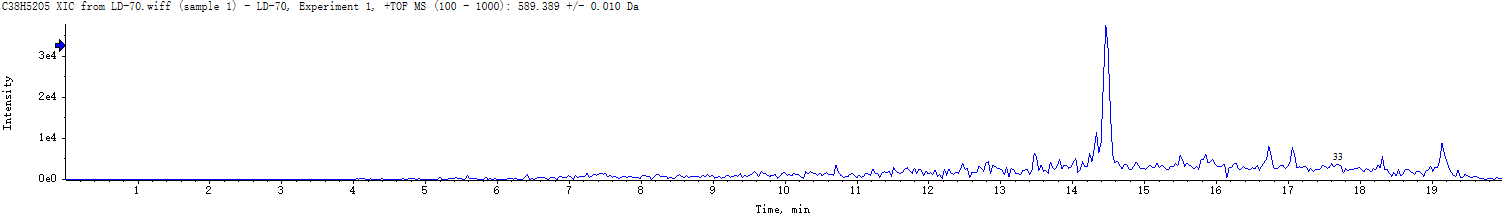


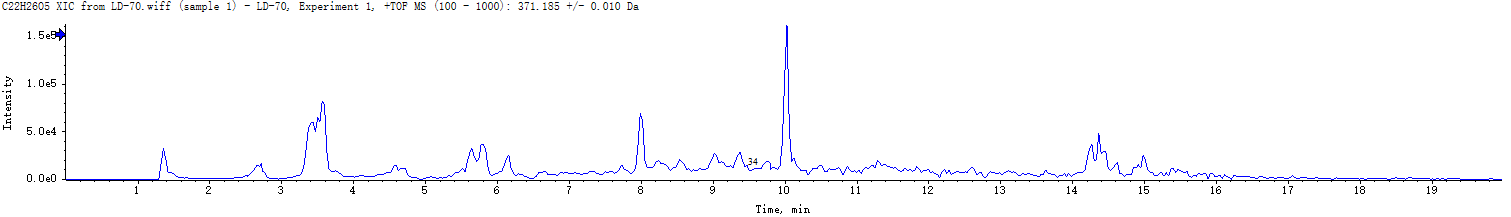


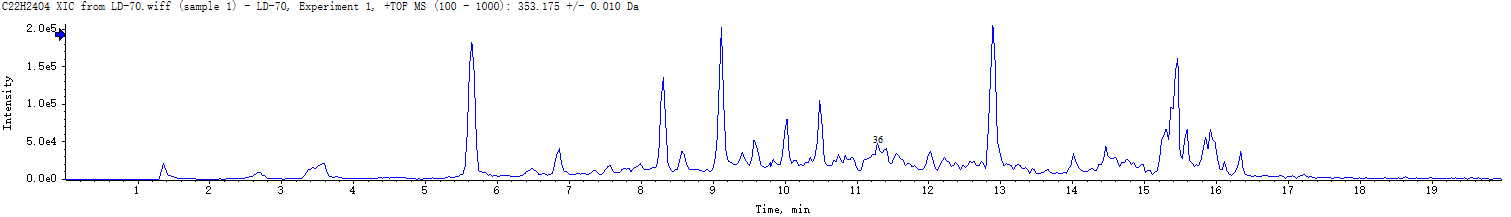


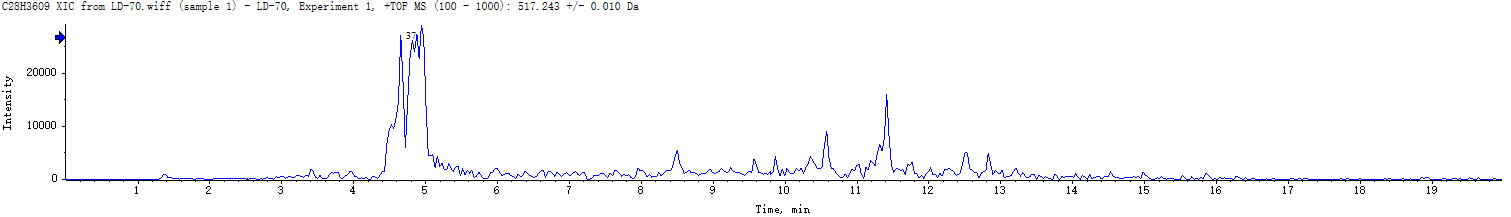


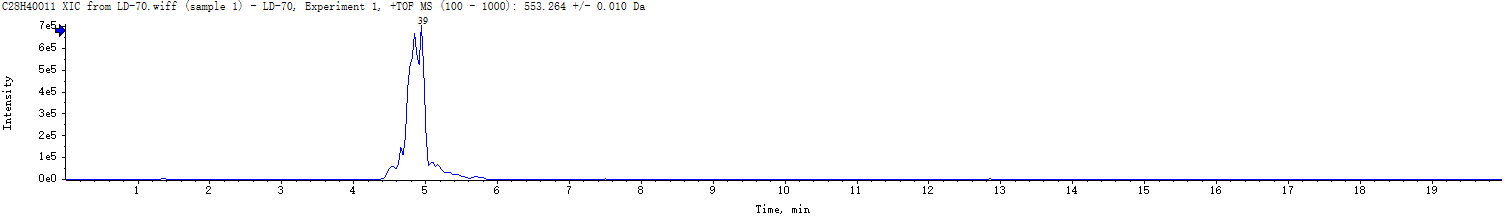


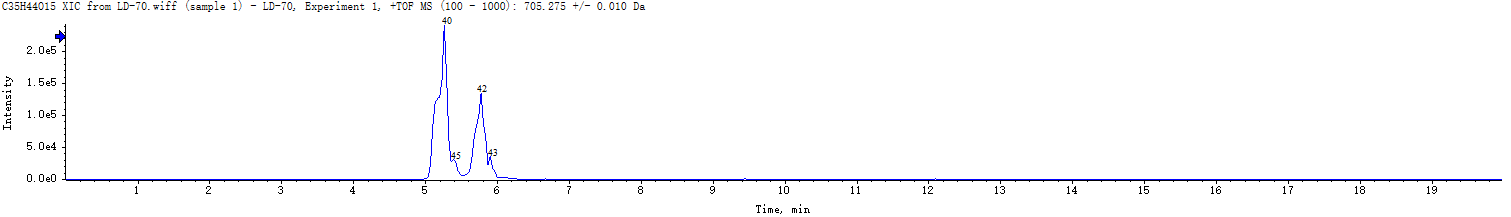


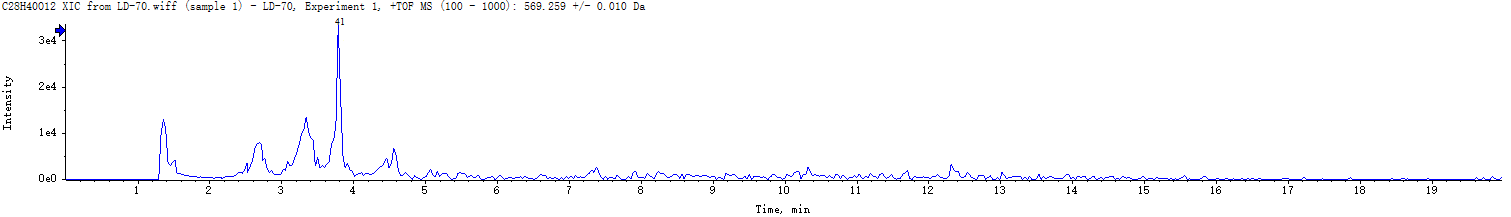


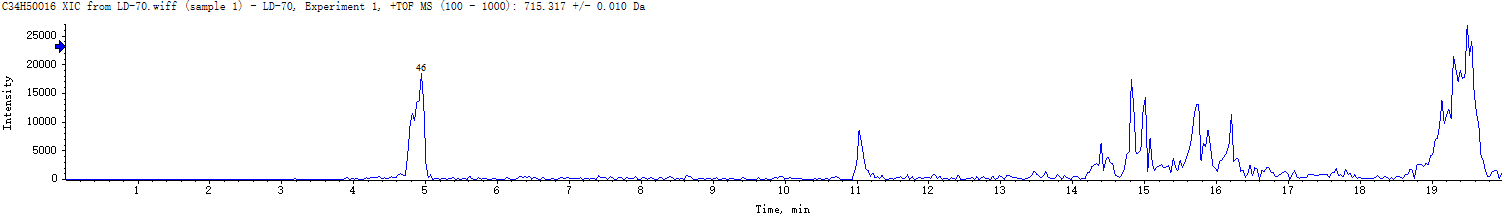


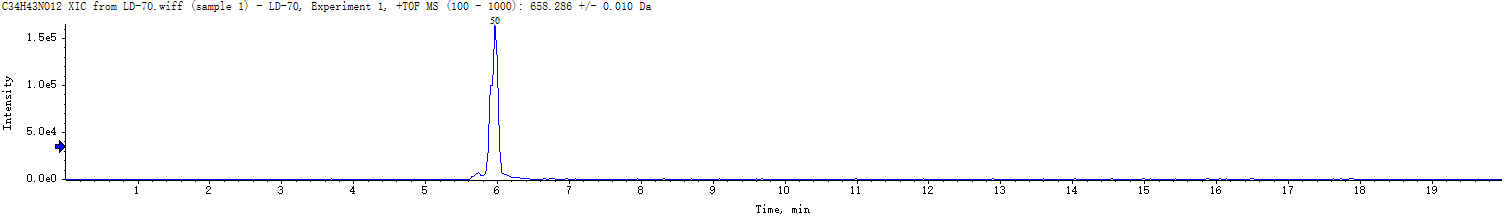


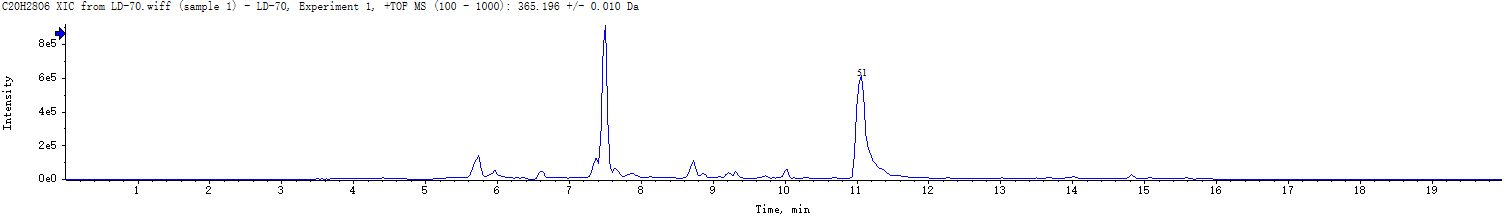


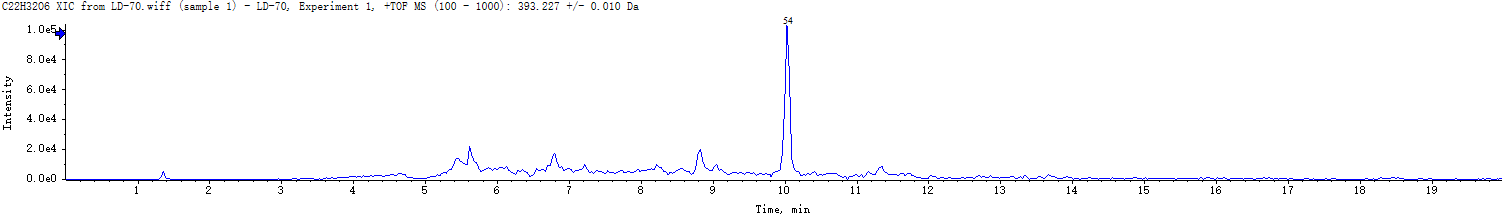


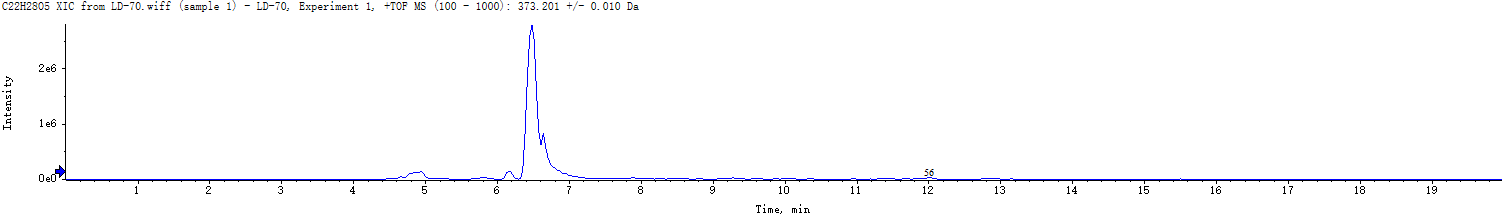


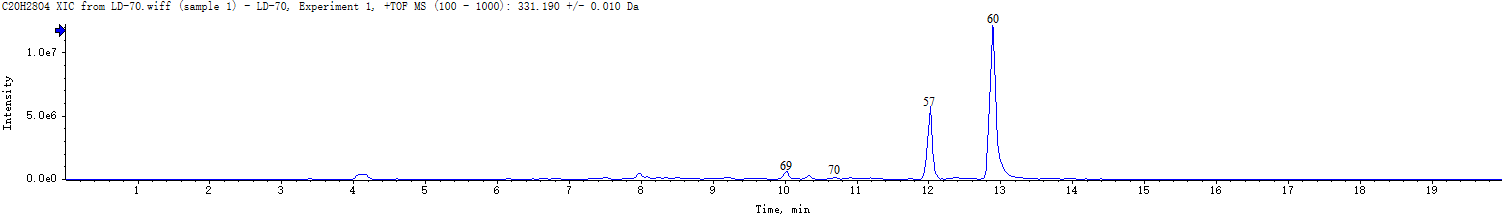


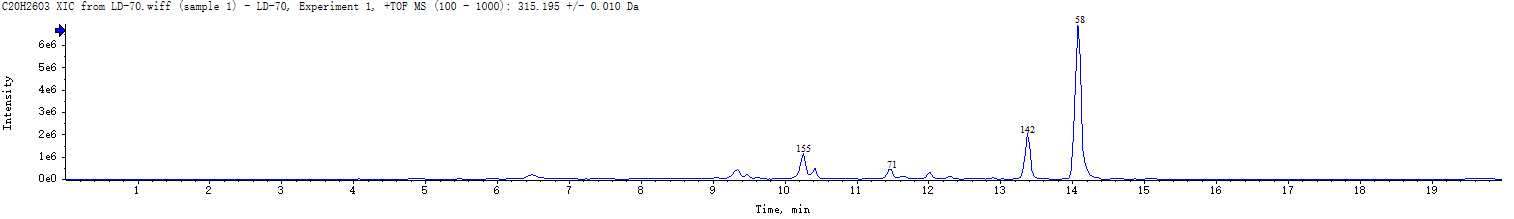


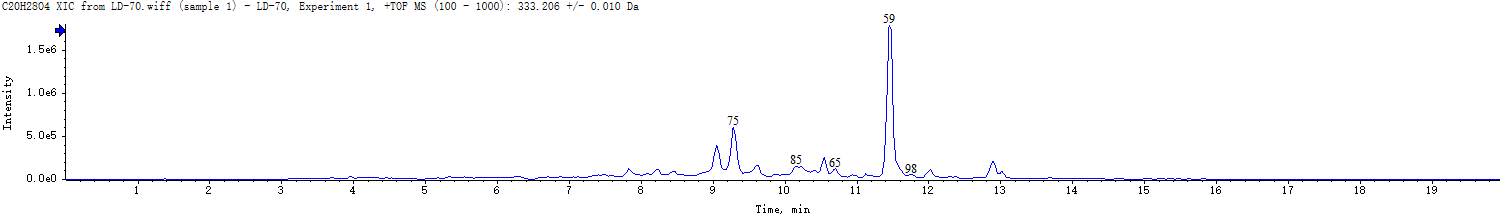


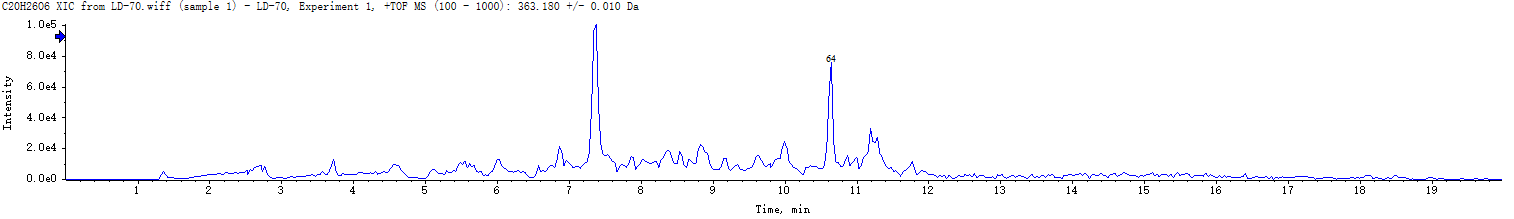


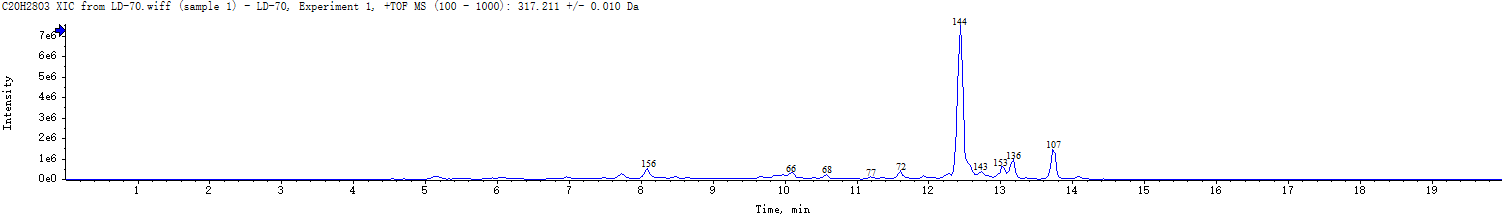


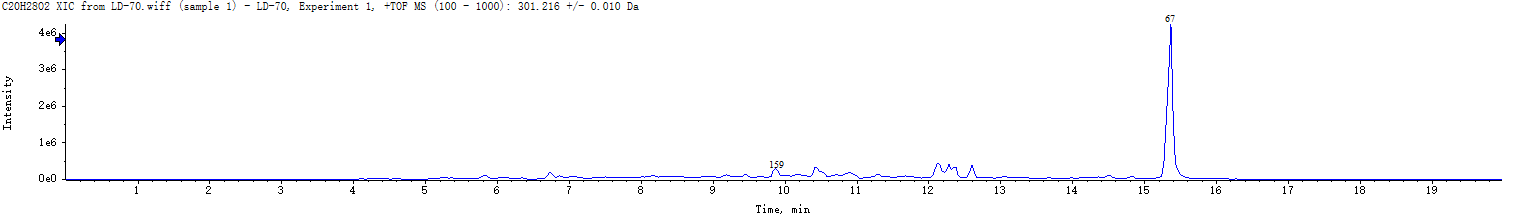


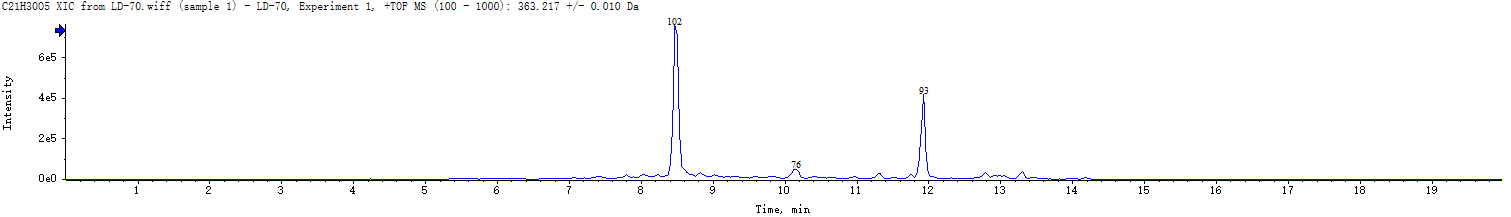


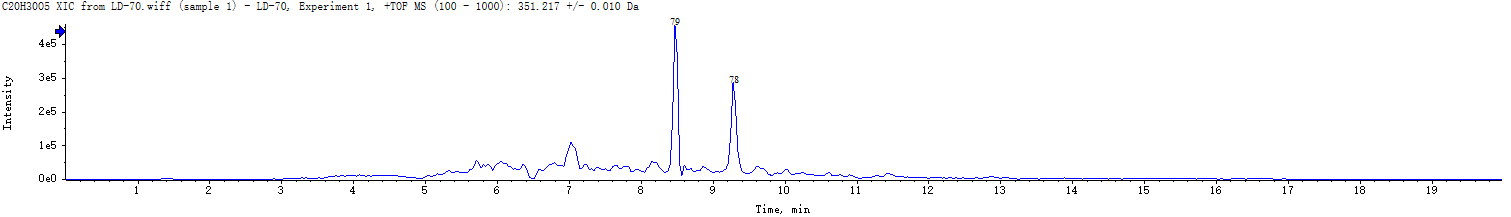


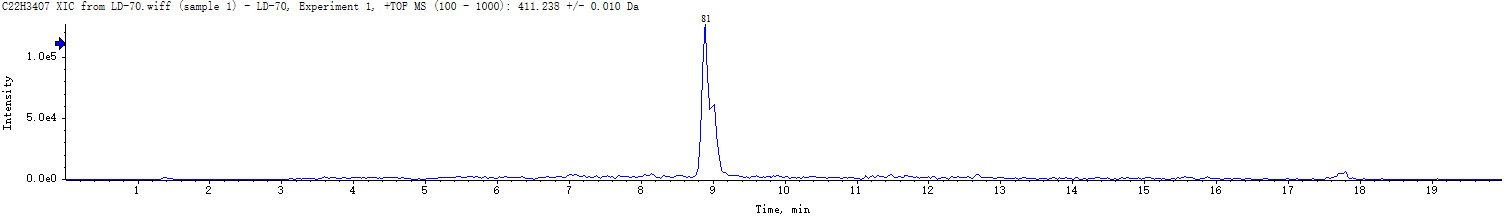


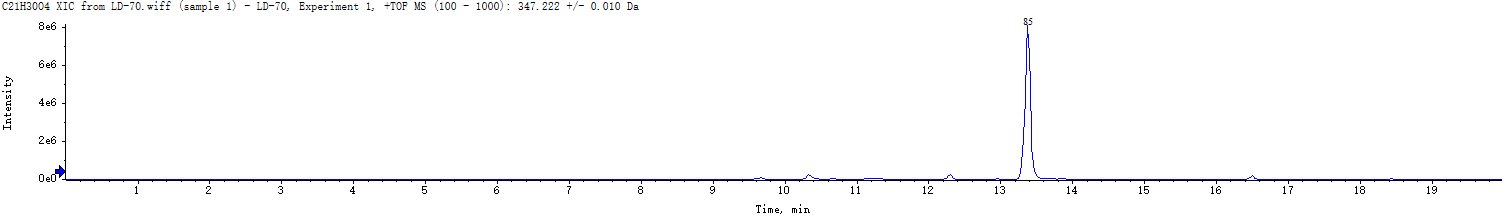


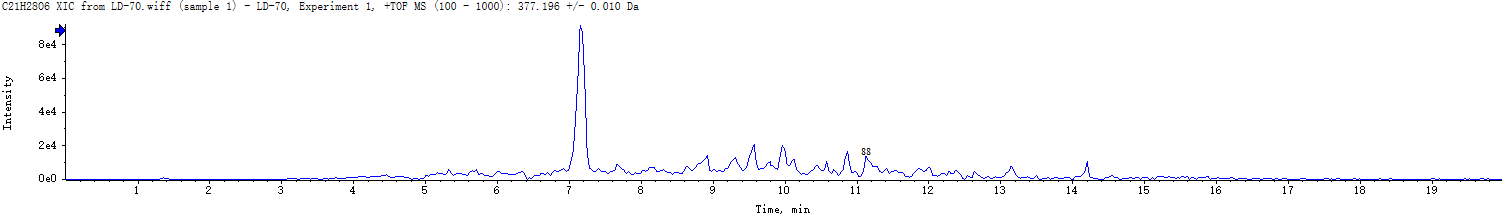


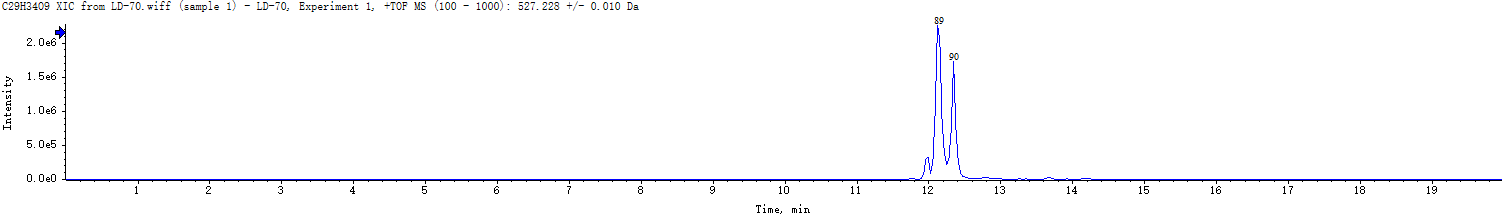


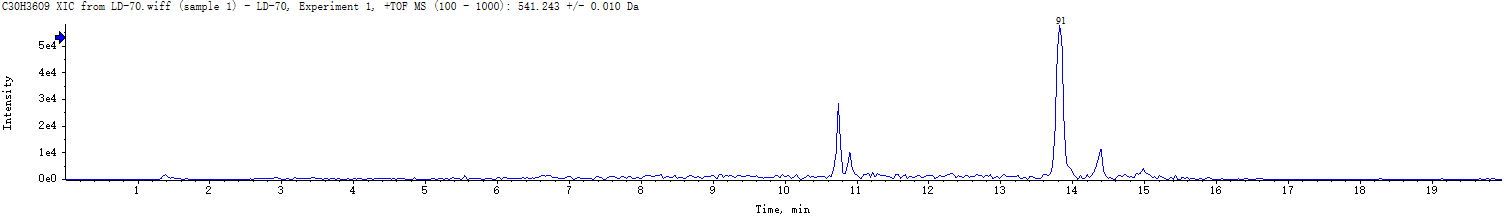


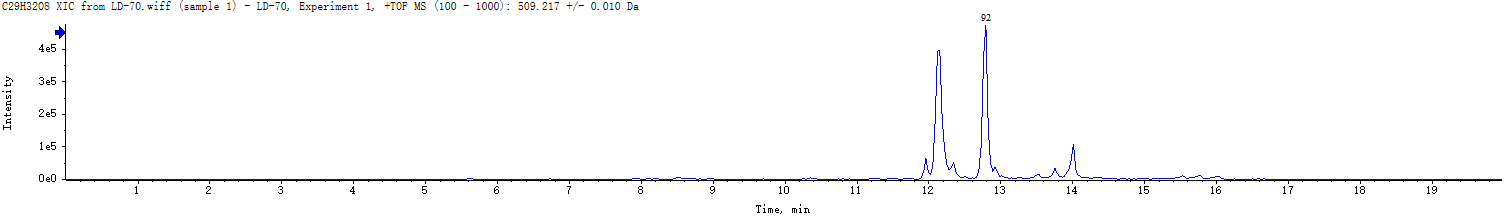


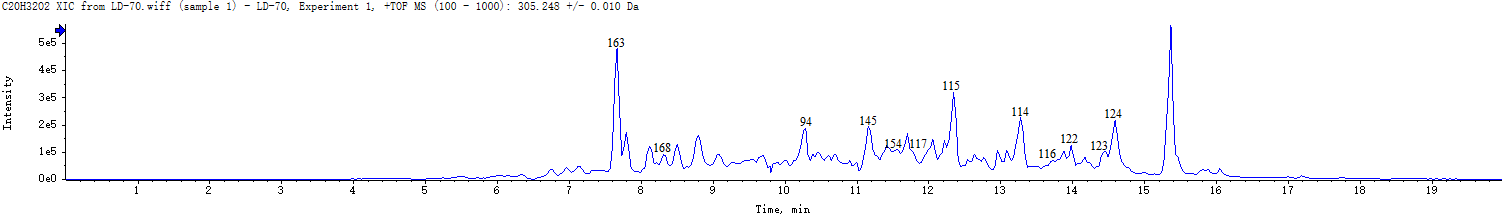


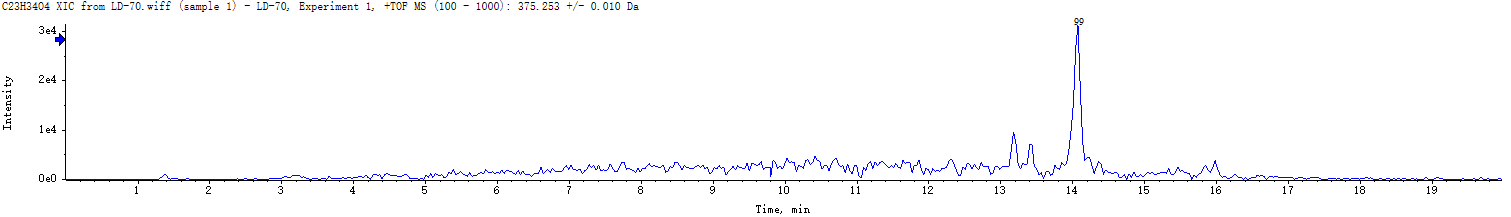


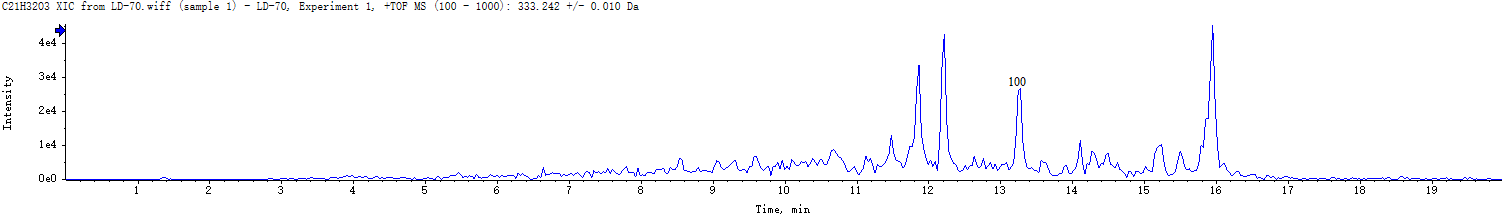


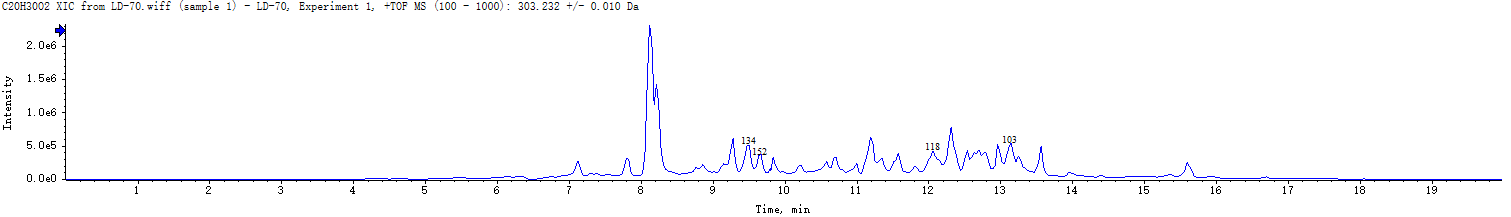


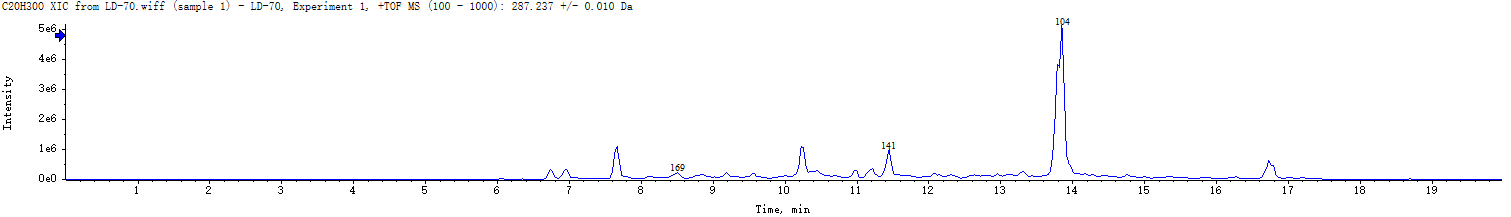


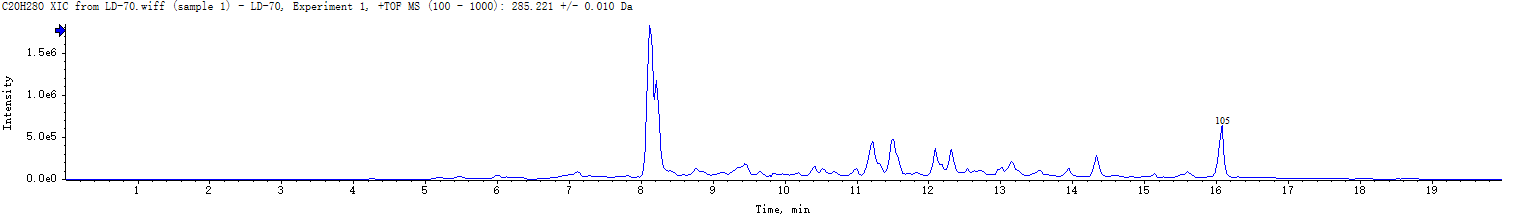


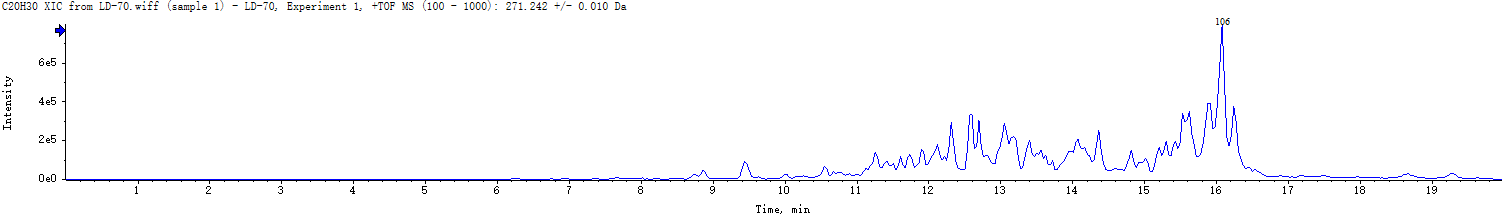


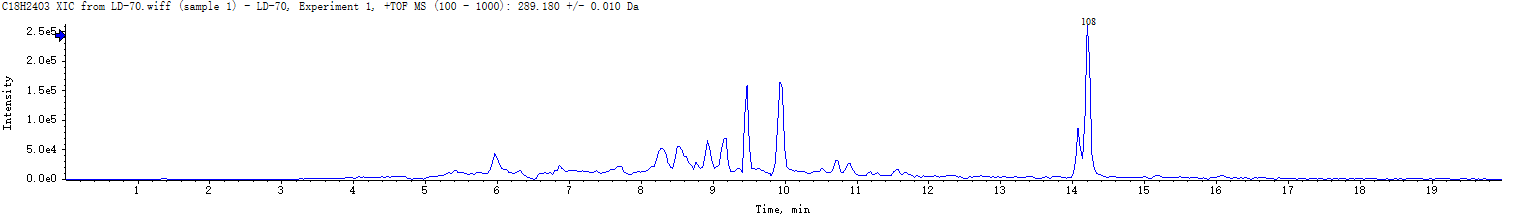


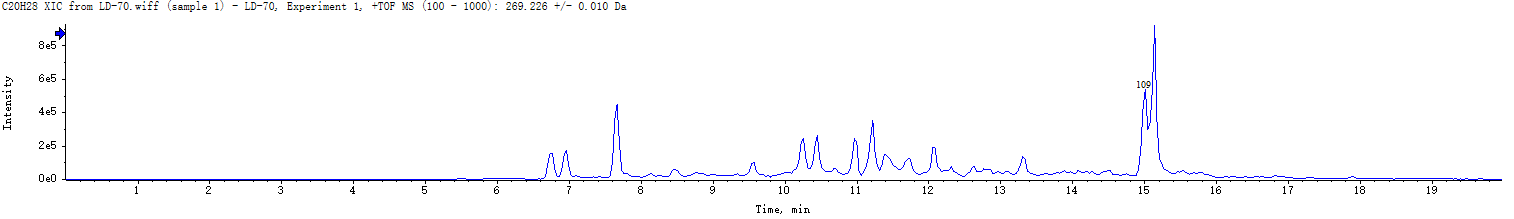


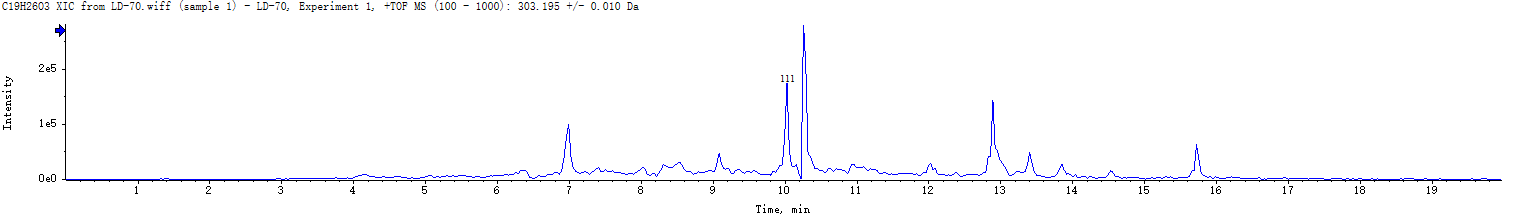


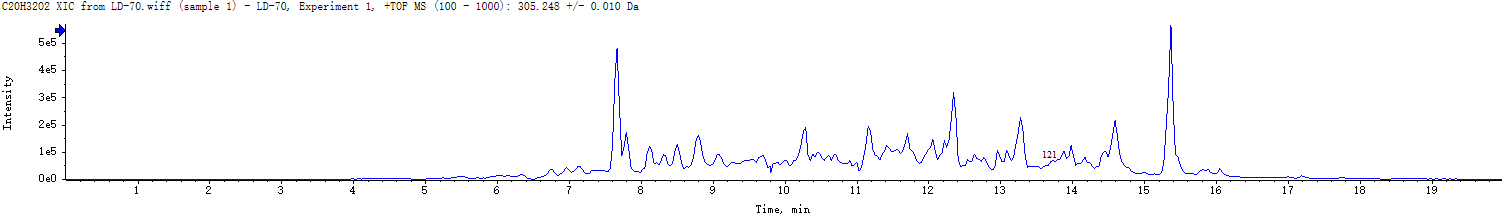


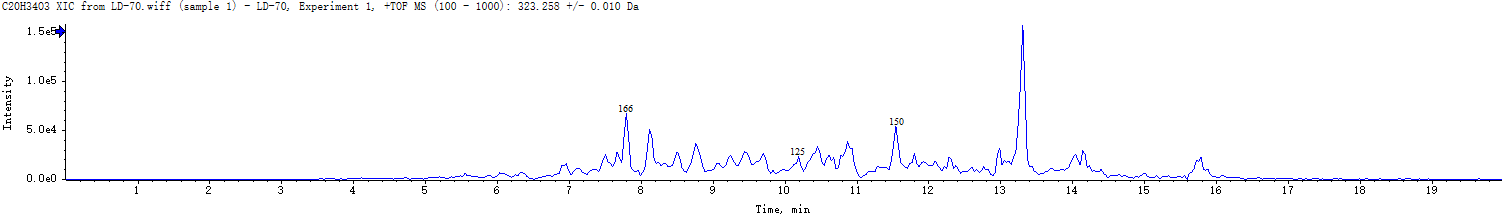


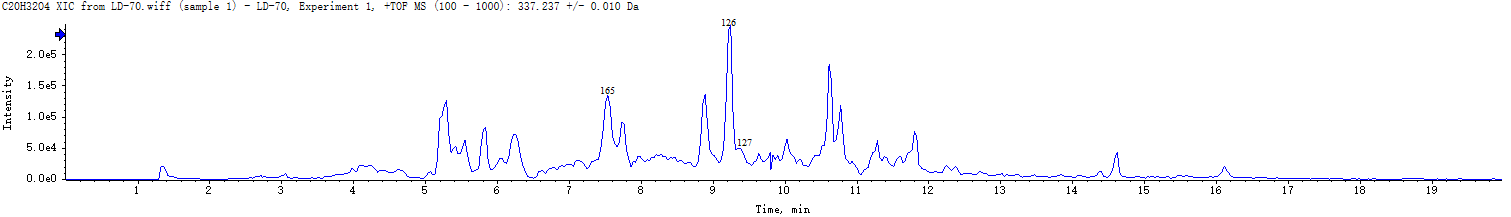


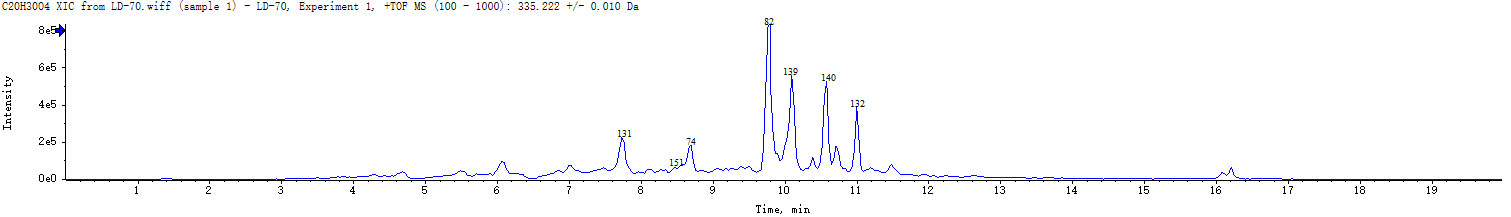


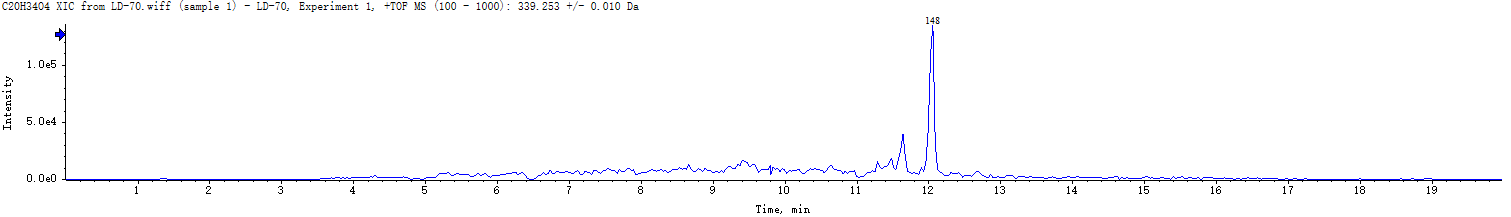


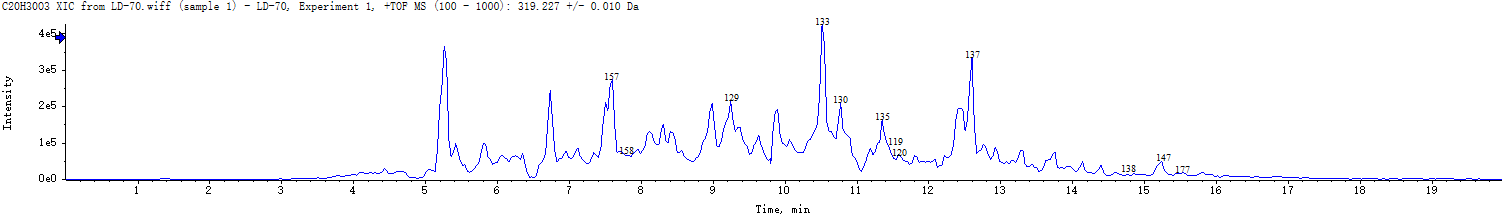


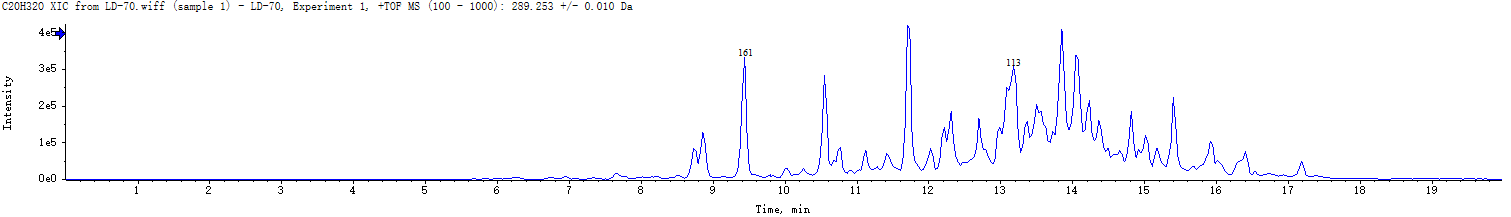


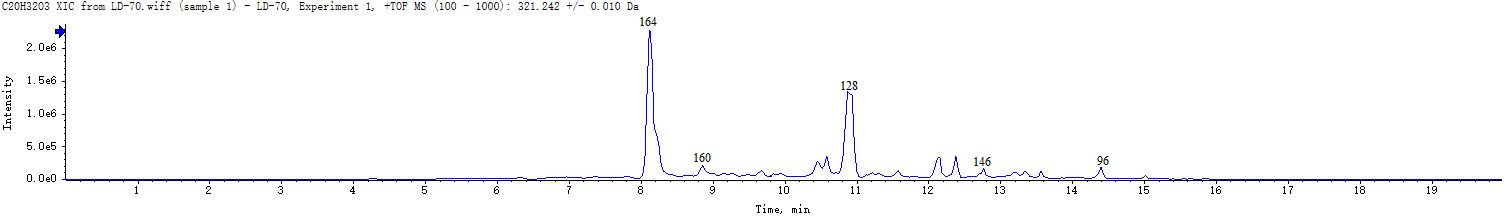


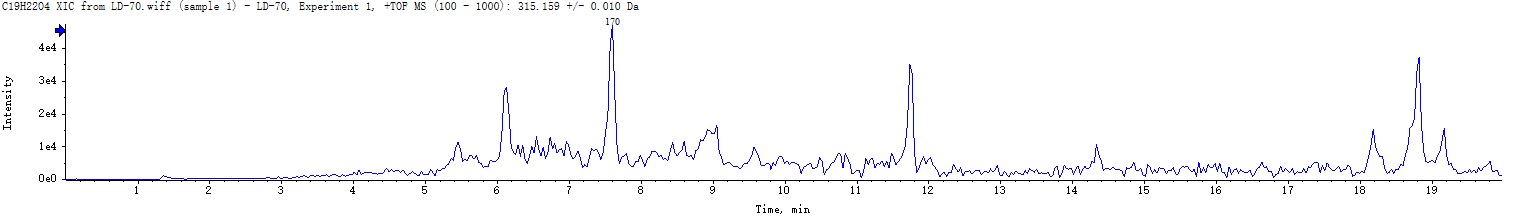


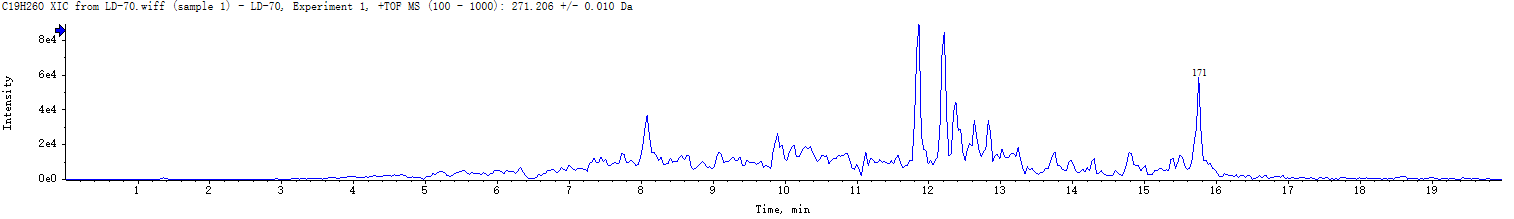


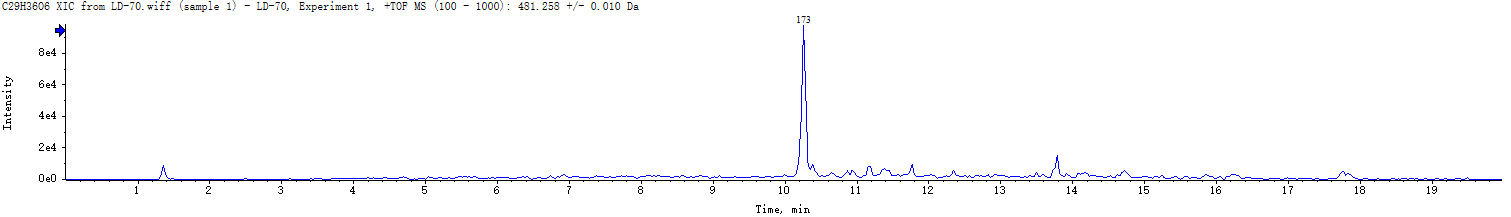


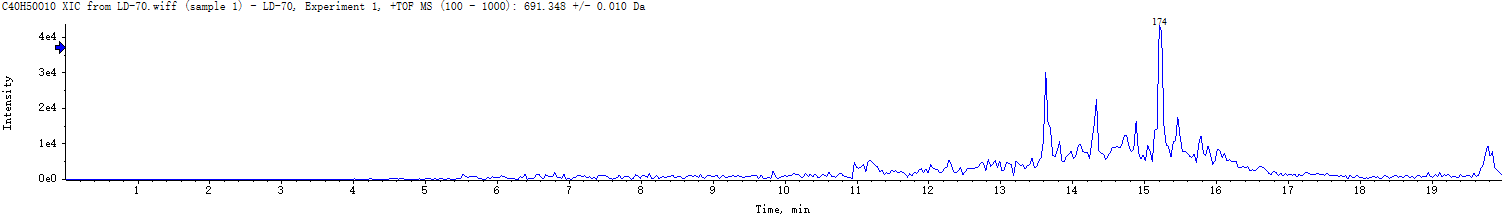


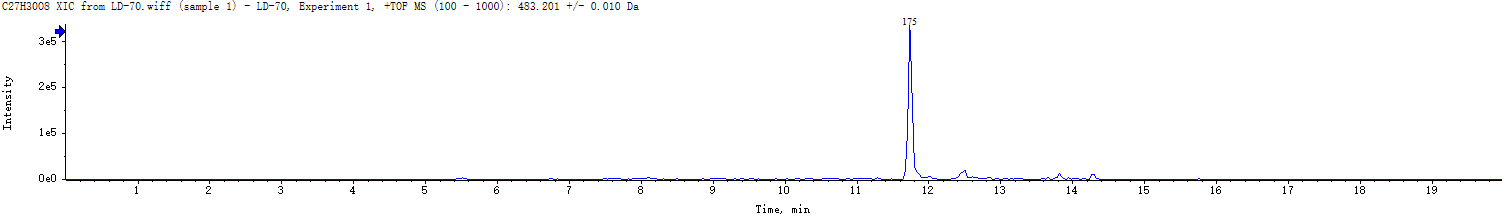


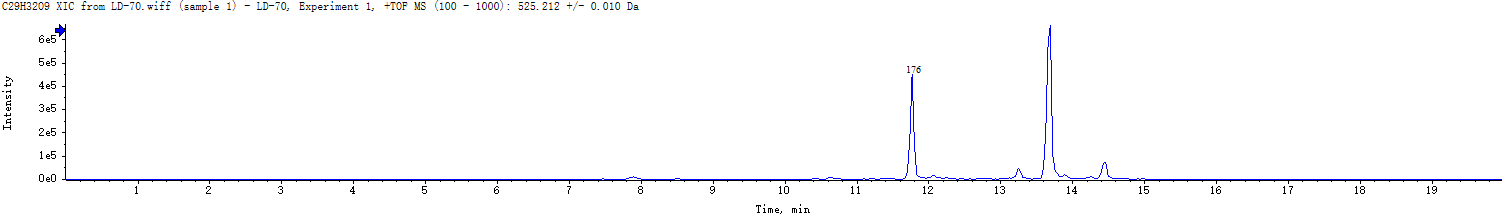


Figure S1 The XIC spectrograms of compounds identified by UHPLC-Q-TOF-MS


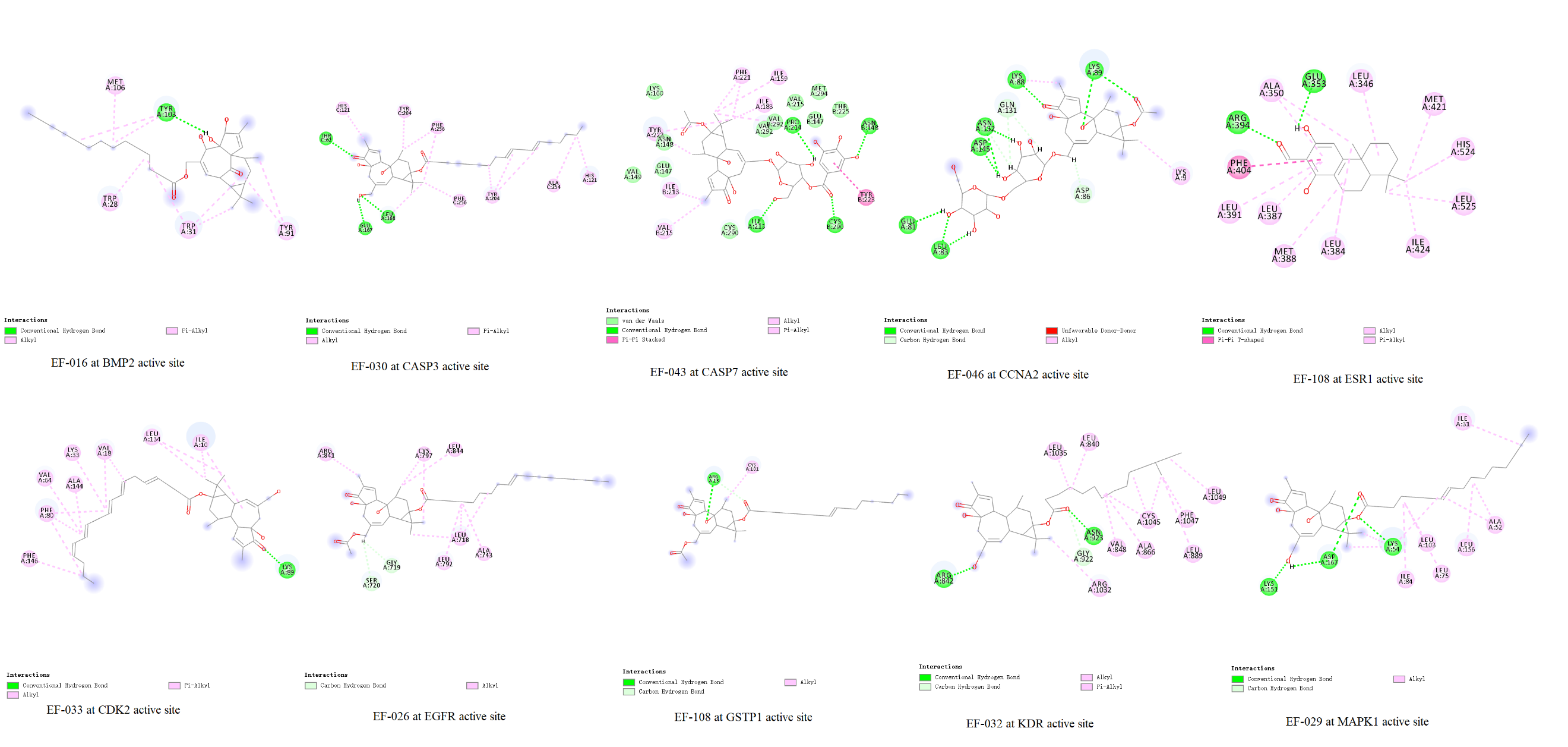


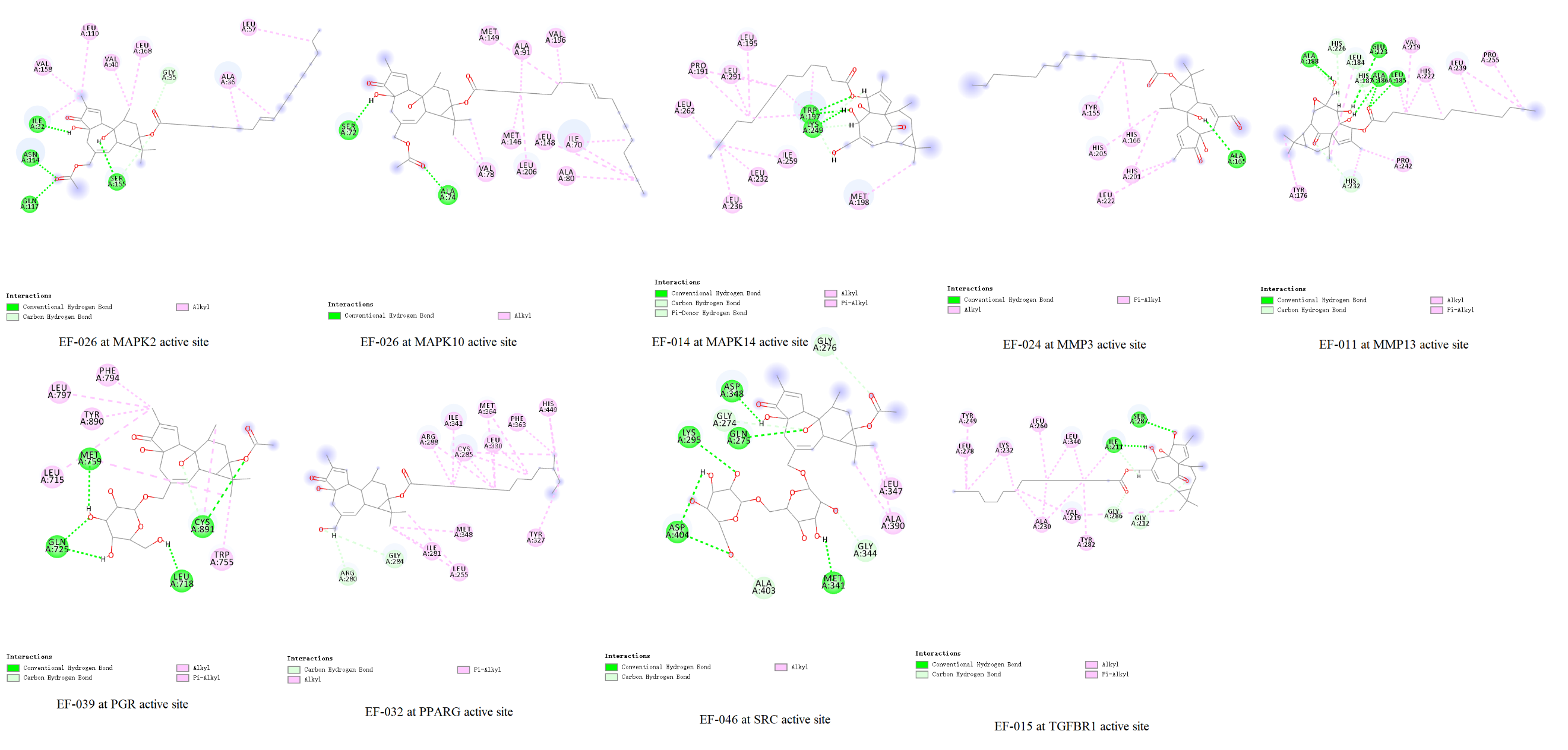


Figure S2 The visualization of intermolecular forces between targets and compounds

Table S1 The information of 177 diterpenoids

| No. | Name | Type | Formula | T_R_ (min) | Experimental mass (*m/z*) | Error (ppm) | MS/MS |
| --- | --- | --- | --- | --- | --- | --- | --- |
| EF-001 | langduin A | daphnane diterpene | C_20_H_28_O_5_ | 7.40 | 349.1997 | -3.7 | 331.2, 315.0, 313.2, 303.2, 285.2, 275.2, 271.2, 267.2, 257.2, 239.2, 229.1, 203.1, 201.1, 193.0, 189.1, 175.0, 147.0, 105.1 |
| EF-002 | euphopiloside A | daphnane diterpene | - | - | - | - | - |
| EF-003 | 4β,9α,20-trihydroxy-13,15-secotiglia-1,6-diene-3,13-dione 20-O-β-D-(6-galloyl)glucopyranoside | daphnane diterpene | - | - | - | - | - |
| EF-004 | langduin A6 | daphnane diterpene | C_22_H_30_O_6_ | 6.86 | 391.2099 | -4.2 | 373.2, 355.2, 347.2, 331.1, 313.2, 295.2, 285.1, 277.2, 271.1, 265.2, 249.1, 239.2, 233.1, 221.1, 211.1, 201.1, 195.1, 185.1, 177.1, 169.1, 149.0, 139.1, 133.1, 121.1 |
| EF-005 | (3aR,6aS,7S,8S,10R,10aR,10bR)-3a-hydroxy-7-isopropyl-8-methoxy-2,10-dimethyl-5-((((2R,3R,5S,6R)-3,4,5-trihydroxy-6-(hydroxymethyl)tetrahydro-2H-pyran-2-yl)oxy)methyl)-3a,4,6a,7,8,9,10,10b-octahydro-3H-8,10a-epoxybenzo[e]azulen-3-one | daphnane diterpene | - | - | - | - | - |
| EF-006 | (3aR,6aS,10R,10aR,10bS)-3a,10a-dihydroxy-5-(hydroxymethyl)-2,10-dimethyl-7-(propan-2-ylidene)-3a,4,6a,7,10,10a-hexahydrobenzo[e]azulene-3,8(9H,10bH)-dione | daphnane diterpene | C_20_H_26_O_5_ | 7.37 | 347.1843 | 1.4 | 329.2, 311.2, 301.2, 283.2, 265.2, 255.2, 241.1, 227.1, 233.1, 213.1, 209.0, 193.0, 181.1, 165.1, 163.1, 135.0, 109.1 |
| EF-007 | (3aR,6aS,7R,10R,10aR,10bS)-3a,10a-dihydroxy-5-(hydroxymethyl)-2,10-dimethyl-7-(prop-1-en-2-yl)-3a,4,6a,7,10,10a-hexahydrobenzo[e]azulene-3,8(9H,10bH)-dione | daphnane diterpene | C_20_H_26_O_5_ | 7.15 | 347.1833 | -5.6 | 329.2, 311.2, 301.2, 285.2, 283.2, 273.2, 265.2, 255.2, 241.1, 227.1, 213.1, 209.0, 201.1, 191.0, 181.1, 173.1, 163.0, 157.1, 119.1 |
| EF-008 | ebractenoid B | diterpenoid lactone | - | - | - | - | - |
| EF-009 | fischeria A | diterpenoid lactone | C_19_H_28_O_2_ | 8.05 | 289.2151 | -4.0 | 271.2, 253.2, 243.2, 229.2, 215.1, 211.1, 199.1, 197.1, 189.1, 185.1, 175.1, 173.1, 169.1, 159.1, 149.1, 145.1, 133.1, 121.1, 119.1, 107.1, 105.1 |
| EF-010 | euphorin D | diterpenoid lactone | - | - | - | - | - |
| EF-011 | ingenol 6,7-epoxy-3-tetradecanoate | ingenane diterpene | - | - | - | - | - |
| EF-012 | ingenol | ingenane diterpene | C_20_H_28_O_5_ | 7.79 | 349.2006 | -1.0 | 331.2, 313.2, 295.2, 285.2, 267.2, 257.1, 239.2, 231.1, 225.1, 203.1, 193.0, 183.1, 175.0, 161.1, 147.0, 137.1, 119.0, 105.1 |
| EF-013 | ingenol-3-palmitate | ingenane diterpene | - | - | - | - | - |
| EF-014 | ingenol-3-myristinate | ingenane diterpene | - | - | - | - | - |
| EF-015 | ingenol-20-palmitate | ingenane diterpene | - | - | - | - | - |
| EF-016 | ingenol-20-myristinate | ingenane diterpene | - | - | - | - | - |
| EF-017 | (1bS,5S,7bS,8R,9R,9aS)-3-(hydroxymethyl)-1,1,6,8-tetramethyl-1a,1b,4,5,7b,8,9,9a-octahydro-1H-cyclopropa[3,4]benzo[1,2-e]azulene-5,7b,9,9a-tetraol | tigliane diterpene | C_20_H_28_O_5_ | 7.86 | 349.2001 | -2.5 | 331.2, 313.2, 303.2, 295.2, 285.2, 271.2, 267.2, 257.2, 249.2, 239.2, 231.1, 225.1, 215.1, 207.1, 193.0, 189.1, 175.0, 169.1, 159.1, 147.0, 145.1, 137.1, 131.1, 123.1, 119.1 |
| EF-018 | phorbolol | tigliane diterpene | C_20_H_30_O_6_ | 7.54 | 367.2104 | -3.0 | 349.2, 331.2, 313.2, 301.2, 295.2, 285.2, 267.2, 257.1, 249.2, 239.2, 231.1, 225.1, 215.1, 203.1, 193.0, 189.1, 185.1, 175.0, 161.1, 147.0, 143.1, 133.1, 123.1, 109.1, 105.1 |
| EF-019 | phorbol-13-acetate | tigliane diterpene | C_21_H_30_O_7_ | 9.54 | 395.2059 | -1.3 | 377.2, 359.2, 345.2, 355.2, 331.2, 327.2, 313.2, 299.2, 285.2, 281.2, 271.2, 263.1, 249.1, 229.2, 221.1, 217.1, 189.1, 187.1, 151.1, 149.1, 147.1, 121.1 |
| EF-020 | 12-deoxyphorbol-13-acetate-prostratin | tigliane diterpene | C_21_H_30_O_6_ | 9.05 | 379.2113 | -0.7 | 361.2, 347.2, 343.2, 329.2, 315.2, 311.2, 301.2, 283.2, 285.2, 283.2, 273.2, 265.2, 255.2, 247.1, 233.1, 213.1, 205.0, 191.0, 177.1, 163.0, 147.0, 135.0, 119.1, 105.1 |
| EF-021 | 12-deoxyphorbol-13-hexadecanoate | tigliane diterpene | - | - | - | - | - |
| EF-022 | 12-deoxyphorbaldehyde-13-acetate | tigliane diterpene | C_22_H_28_O_6_ | 9.83 | 389.1948 | -2.6 | 371.2, 353.2, 329.2, 311.2, 293.2, 283.2, 267.1, 265.2, 243.2, 237.2, 233.1, 211.1, 209.1, 199.1, 193.1, 185.1, 173.1, 131.1, 119.1, 111.0 |
| EF-023 | 20-oxo-prostratin | tigliane diterpene | C_22_H_28_O_7_ | 8.27 | 405.1907 | -0.3 | 387.2, 369.3.355.2, 345.2, 327.2, 309.2, 297.1, 281.2, 271.2, 263.1, 253.1, 251.1, 233.1, 223.1, 219.1, 201.1, 195.1, 181.0, 173.1, 167.1, 145.1, 135.1, 123.0, 107.0 |
| EF-024 | 12-deoxyphorbaldehyde-13-hexadecanoate | tigliane diterpene | - | - | - | - | - |
| EF-025 | prostratin | tigliane diterpene | C_22_H_30_O_6_ | 7.79 | 391.21152 | -7.78 | 373.3, 349.1, 331.2, 313.1, 298.2, 285.1, 271.1, 267.1, 253.1, 239.1, 225.1, 219.1, 201.1, 195.1, 183.1, 177.1, 163.0, 149.0, 135.1, 129.1, 107.1 |
| EF-026 | 12-deoxyphorbol-13-[(9Z)-octadec-9-enoate]-20-acetate | tigliane diterpene | - | - | - | - | - |
| EF-027 | 12-deoxyphorbol 13-acetate | tigliane diterpene | - | - | - | - | - |
| EF-028 | 12-deoxyphorbol | tigliane diterpene | C_20_H_28_O_5_ | 8.56 | 349.2007 | -0.7 | 331.2, 317.2, 313.2, 303.2, 295.2, 285.2, 267.2, 257.2, 249.2, 239.2, 231.1, 225.1, 215.1, 201.1, 193.0, 177.1, 175.0, 161.1, 147.0, 137.1, 123.1, 109.1, 105.1 |
| EF-029 | 12-deoxyphorbol-13-(7Z)-hexadecenoate | tigliane diterpene | - | - | - | - | - |
| EF-030 | 12-deoxyphorbol-13-(9Z,12Z)-octadecadienoate | tigliane diterpene | - | - | - | - | - |
| EF-031 | 12-deoxyphorbol-13-(6Z)-octadecenoate | tigliane diterpene | - | - | - | - | - |
| EF-032 | 12-deoxyphorbol-13-dimethylpentadecanoate | tigliane diterpene | - | - | - | - | - |
| EF-033 | 12-deoxyphorbol-13-(4Z,7E,9E,12E,14E)-octadeca-4,7,9,12,14-pentaenoate | tigliane diterpene | C_38_H_52_O_5_ | 17.67 | 589.3887 | -0.1 | 571.4, 553.4, 543.4, 493.2, 457.2, 433.2, 407.3, 3939.2, 349.3, 333.2, 315.2, 295.2, 277.2, 259.2, 253.2, 227.1, 177.1, 163.1, 133.1 |
| EF-034 | (1aR,4aR,7aS,8R,9aS)-3-formyl-4a-hydroxy-1,1,6,8-tetramethyl-5-oxo-1,1a,4,4a,5,7a,8,9-octahydro-9aH-cyclopropa[3,4]benzo[1,2-e]azulen-9a-yl acetate | tigliane diterpene | C_22_H_26_O_5_ | 9.54 | 371.1841 | -3.3 | 353.3, 335.2, 329.2, 311.2, 293.1, 279.2, 267.2, 265.2, 261.2, 237.2, 225.1, 223.1, 207.1, 199.1, 195.1, 1, 175.1, 173.1, 161.1, 149.1, 135.1, 123.1, 121.1, 113.1, 109.1, 105.1 |
| EF-035 | ((1aR,4aR,7aS,8S,9aS)-9a-acetoxy-4a,8-dihydroxy-1,1,6,8-tetramethyl-5-oxo-1a,2,4a,5,7a,8,9,9a-octahydro-1H-cyclopropa[3,4]benzo[1,2-e]azulen-3-yl)methyl acetate | tigliane diterpene | C_24_H_30_O_7_ | 12.23 | 431.2051 | -3.0 | 413.2, 395.3, 371.2, 353.2, 329.2, 311.2, 283.2, 267.1, 265.2, 251.1, 249.1, 245.1, 235.1, 227.1, 223.1, 213.1, 211.1, 209.1, 203.1, 199.1, 195.1, 187.1, 185.1, 181.1, 175.1, 169.1, 161.1, 121.1, 105.1 |
| EF-036 | 1aR,1bS,8R,9aS)-3-formyl-1,1,6,8-tetramethyl-5-oxo-1,1a,1b,5,8,9-hexahydro-9aH-cyclopropa[3,4]benzo[1,2-e]azulen-9a-yl acetate | tigliane diterpene | C_22_H_24_O_4_ | 11.32 | 353.1752 | 1.2 | 335.3, 311.2, 293.2, 275.1, 263.1, 261.2, 251.1, 247.1, 243.2, 235.1, 223.1, 217.1, 207.1, 193.1, 187.1, 173.1, 163.1, 145.1, 131.1, 121.1, 107.1, 105.1 |
| EF-037 | (1aR,7aS,8R,9aS)-1,1,6,8-tetramethyl-5-oxo-3-((((2R,3R,4S,5S,6R)-3,4,5-trihydroxy-6-(hydroxymethyl)tetrahydro-2H-pyran-2-yl)oxy)methyl)-1,1a,5,7a,8,9-hexahydro-9aH-cyclopropa[3,4]benzo[1,2-e]azulen-9a-yl acetate | tigliane diterpene | C_28_H_36_O_9_ | 4.69 | 517.2426 | -1.3 | 499.2, 481.2, 463.3, 457.2, 439.2, 421.2, 403.2, 391.1, 379.2, 355.2, 337.2, 313.2, 303.1, 295.2, 283.2, 277.2, 265.2, 253.1, 235.1, 225.1, 211.1, 207.1, 187.1, 169.1, 149.1, 127.0, 107.1 |
| EF-038 | ((1aR,8S,9aS)-9a-acetoxy-8-hydroxy-1,1,6,8-tetramethyl-5-oxo-1a,2,5,8,9,9a-hexahydro-1H-cyclopropa[3,4]benzo[1,2-e]azulen-3-yl)methyl acetate | tigliane diterpene | - | - | - | - | - |
| EF-039 | fischeroside A | tigliane diterpene | C_28_H_40_O_11_ | 4.88 | 553.2645 | 0.4 | 313.2, 295.2, 279.2, 277.2, 267.2, 265.2, 255.1, 249.2, 235.1, 225.1, 207.1. 193.1, 185.1, 133.1 |
| EF-040 | fischeroside B | tigliane diterpene | C_35_H_44_O_15_ | 5.16 | 705.2760 | 1.1 | 609.2, 315.1, 313.2, 295.2, 279.2, 277.2, 267.2, 249.2, 153.0 |
| EF-041 | fischeroside C | tigliane diterpene | C_28_H_40_O_12_ | 3.82 | 569.2583 | -1.6 | 329.2, 311.2, 293.2, 283.2, 275.1, 269.2, 265.2, 247.1, 241.1, 237.2, 223.1, 205.1, 201.1, 195.1, 187.1, 183.1, 177.1, 171.1, 165.1, 155.1 |
| EF-042 | prostratin 20-O-(3′-galloyl)-β-D-glucopyranoside | tigliane diterpene | C_35_H_44_O_15_ | 5.80 | 705.2766 | 1.9 | 357.1, 315.1, 313.2, 295.2, 279.2, 277.2, 267.2, 253.1, 249.2, 153.0 |
| EF-043 | prostratin 20-O-(4′-galloyl)-β-D-glucopyranoside | tigliane diterpene | C_35_H_44_O_15_ | 5.84 | 705.2733 | -2.8 | 315.1, 313.2, 297.2, 295.2, 279.2, 277.2, 267.2, 253.1, 249.2, 225.1, 207.1, 153.0 |
| EF-044 | prostratin 20-O-(6′-acetate)-β-D-glucopyranoside | tigliane diterpene | - | - | - | - | - |
| EF-045 | prostratin 20-O-(2′-galloyl)-β-D-glucopyranoside | tigliane diterpene | C_35_H_44_O_15_ | 5.26 | 705.2754 | 0.1 | 609.2, 357.1, 315.1, 313.2, 295.2, 279.2, 277.2, 267.2, 253.1, 249.2, 235.1, 225.1, 153.0 |
| EF-046 | prostratin 20-O-(6′-glucosyl)- β-D-glucopyranoside | tigliane diterpene | C_34_H_50_O_16_ | 4.94 | 715.3193 | 3.0 | 553.3, 475.2, 457.2, 373.2, 355.2, 313.2, 295.2, 277.2, 267.2, 265.2, 205.1, 185.1 |
| EF-047 | euphosantianane E | premyrsinane diterpene | - | - | - | - | - |
| EF-048 | euphosantianane F | premyrsinane diterpene | - | - | - | - | - |
| EF-049 | euphosantianane G | premyrsinane diterpene | - | - | - | - | - |
| EF-050 | (1aS,3S,3aR,4R,4aR,5R,6S,7aR,9R,9aR,9bS)-7a-hydroxy-1,1,6,9-tetramethyl-3a-((nicotinoyloxy)methyl)-8-oxotetradecahydro-1H-cyclopropa[3,4]benzo[1,2-f]azulene-3,4,5,9-tetrayl tetraacetate | premyrsinane diterpene | C_34_H_43_NO_12_ | 5.96 | 658.2867 | 1.4 | 640.3, 598.3, 580.3, 562.2, 313.2, 295.2, 286.1, 279.2, 277.2, 268.1, 262.1, 253.1, 249.2, 235.1, 207.1, 124.0 |
| EF-051 | langduin B | ent-abietane diterpene | C_20_H_28_O_6_ | 11.03 | 365.1953 | -1.5 | 347.2, 329.2, 321.2, 311.2, 301.2, 293.2, 287.2, 283.2, 273.2, 269.2, 261.1, 259.1, 255.2, 247.1, 241.2, 227.1, 219.1, 213.1, 205.0, 199.1, 193.0, 191.0, 185.1, 177.1, 165.1, 147.0, 143.1, 135.0, 133.1, 125.0, 119.1, 105.1 |
| EF-052 | 7-deoxylangduin B | ent-abietane diterpene | C_20_H_28_O_5_ | 11.61 | 349.1998 | -3.3 | 331.2, 317.2, 303.2, 299.2, 285.2, 271.2, 257.2, 243.1, 233.1, 227.2, 217.1, 207.1, 201.1, 189.1, 183.1, 175.1, 161.1, 159.1, 147.1, 133.1, 125.1, 111.0, 105.1 |
| EF-053 | eupholide C | ent-abietane diterpene | C_21_H_30_O_6_ | 6.76 | 379.2134 | 4.9 | 361.2, 343.2, 329.2, 315.2, 311.2, 301.2, 283.2, 265.2, 255.2, 247.1, 241.1, 231.1, 223.1, 213.1, 205.0, 199.1, 191.0, 187.1, 177.1, 163.0, 155.1, 145.1, 133.1, 119.1, 105.1 |
| EF-054 | eupholide D | ent-abietane diterpene | C_22_H_32_O_6_ | 10.06 | 393.2265 | -1.8 | 375.2, 357.2, 347  .2, 333.2, 329.2, 311.2, 301.2, 287.2, 285.2, 273.2, 261.1, 255.2, 237.1, 231.2, 227.1, 205.0, 191.0, 175.1, 169.1, 163.1, 153.0, 147.0, 139.0, 133.1, 121.1, 107.1 |
| EF-055 | eupholide E | ent-abietane diterpene | C_21_H_30_O_6_ | 7.05 | 379.2115 | -0.2 | 361.2, 343.2, 333.2, 329.2, 319.2, 315.2, 311.2, 301.2, 293.2, 283.2, 279.1, 265.2, 255.2, 243.2, 237.2, 223.1, 213.1, 205.0, 199.1, 191.0, 187.1, 181.1, 173.1, 163.0, 155.1, 147.0, 137.1, 123.0, 109.1 |
| EF-056 | 17-acetoxyjolkinolide A | ent-abietane diterpene | C_22_H_28_O_5_ | 12.02 | 373.2007 | -0.6 | 355.2, 337.3, 328.2, 313.2, 295.2, 277.2, 267.2, 257.1, 249.2, 239.1, 231.1, 225.1, 217.1, 201.1, 177.1, 173.1, 163.1, 157.1, 149.1, 141.1, 135.1, 119.1, 107.1 |
| EF-057 | 17-hydroxyjolkinolide A | ent-abietane diterpene | C_20_H_26_O_4_ | 12.25 | 331.1910 | 2.0 | 313.2, 299.2, 295.2, 271.2, 253.2, 215.1, 201.1, 187.1, 175.1, 161.1, 147.1, 135.1, 133.1, 119.1, 105.1 |
| EF-058 | jolkinolide A | ent-abietane diterpene | C_20_H_26_O_3_ | 14.05 | 315.1951 | -1.1 | 297.2, 287.2, 269.2, 251.2, 241.1, 233.1, 231.1, 227.1, 219.1, 213.1, 205.1, 199.1, 191.1, 179.1, 177.1, 175.1, 169.1, 163.0, 157.1, 149.1, 139.0, 131.0, 121.1, 107.1, 105.1 |
| EF-059 | euphonoid B | ent-abietane diterpene | C_20_H_28_O_4_ | 11.48 | 333.2055 | -1.5 | 315.2, 297.2, 287.2, 279.2, 273.2, 269.2, 259.1, 251.2, 241.2, 231.1, 227.1, 217.1, 213.1, 199.1, 185.1, 177.1, 171.1, 157.1, 143.1, 139.0, 137.0, 121.01, 111.0, 107.1 |
| EF-060 | jolkinolide B | ent-abietane diterpene | C_20_H_26_O_4_ | 12.86 | 331.1903 | -0.4 | 313.2, 303.2, 295.2, 271.2, 267.2, 257.2, 249.2, 239.2, 229.1, 221.2, 215.1, 203.1, 197.1, 193.0, 189.1, 183.1, 169.1, 165.1, 157.1, 147.0, 137.1, 125.0, 123.1, 109.1, 105.1 |
| EF-061 | 17-hydroxyjolkinolide B | ent-abietane diterpene | C_20_H_26_O_5_ | 11.03 | 347.1843 | 3.0 | 329.2, 317.2, 311.2, 301.2, 299.2, 293.2, 283.2, 273.2, 265.2, 255.2, 241.1, 237.2, 227.2, 215.1, 213.1, 201.1, 195.1, 191.0, 185.1, 163.0, 157.1, 151.1, 143.1, 131.1, 125.0, 109.1 |
| EF-062 | 17-acetoxyjolkinolide B | ent-abietane diterpene | C_22_H_28_O_6_ | 12.60 | 389.1962 | 0.7 | 371.2, 361.3, 353.2, 343.2, 329.2, 311.2, 293.2, 283.2, 267.1, 265.2, 251.1, 241.1, 225.1, 223.1, 227.1, 225.1, 223.1, 221.1, 213.1, 211.1, 209.1, 207.1, 199.1, 197.1, 193.1, 187.1, 185.1, 181.1, 169.1, 163.1, 159.1, 155.1, 147.1, 137.1, 135.1, 121.1, 117.1, 109.1, 105.1 |
| EF-063 | 11α,17-dihydroxyhelioscopinolide E | ent-abietane diterpene | C_20_H_26_O_5_ | 10.19 | 347.1840 | -3.6 | 329.2, 311.2, 301.2, 297.2, 287.2, 283.2, 273.2, 255.2, 241.1, 233.1, 227.1, 213.1, 205.1, 187.1, 177.1, 163.0, 149.1, 123.1, 121.1, 107.1 |
| EF-064 | 6β,11α,17-trihydroxyhelioscopinolide E | ent-abietane diterpene | C_20_H_26_O_6_ | 10.61 | 363.1795 | -2.1 | 345.2, 327.2, 309.1, 295.2, 271.1, 253.1, 245.1, 239.1, 231.1, 221.0, 207.0, 203.0, 191.0, 189.0, 177.0, 161.0, 151.1, 123.1, 109.1 |
| EF-065 | fischeriolide C | ent-abietane diterpene | C_20_H_28_O_4_ | 9.05 | 333.2052 | -2.6 | 315.2, 297.2, 287.2, 279.2, 269.2, 259.2, 251.2, 241.2, 227.1, 213.1, 191.1, 177.1, 175.1, 159.1, 149.1, 139.0, 131.1, 121.1, 105.1 |
| EF-066 | ent-11α-hydroxyabieta-8(14),13(15)-dien-16,12α-olide | ent-abietane diterpene | C_20_H_28_O_3_ | 10.12 | 317.2107 | -0.9 | 299.2, 289.2, 281.2, 271.2, 257.2, 253.2, 243.1, 231.2, 229.2, 225.2, 213.1, 211.1, 203.1, 197.1, 183.1, 177.1, 171.1, 169.1, 157.1, 149.1, 143.1, 139.1, 133.1, 123.1, 121.1, 119.1, 109.1, 107.1, 105.1 |
| EF-067 | jolkinolide E | ent-abietane diterpene | C_20_H_28_O_2_ | 15.33 | 301.2157 | -1.6 | 283.2, 255.2, 245.2, 231.1, 227.1, 219.1, 217.1, 213.1, 205.1, 203.1, 199.1, 191.1, 185.1, 179.1, 177.1, 173.1, 171.1, 163.1, 157.1, 149.1, 145.1, 143.1, 135.1, 131.1, 129.1, 125.1, 123.1, 119.1, 117.1, 115.1, 109.1, 107.1, 105.1 |
| EF-068 | jolkinolide F | ent-abietane diterpene | C_20_H_28_O_3_ | 10.58 | 317.21112 | -3.5 | 299.2, 281.2, 259.1, 257.2, 253.2, 243.1, 229.1, 225.2, 215.1, 211.1, 203.1, 197.1, 189.1, 185.1, 183.1, 179.1, 169.1, 161.1, 157.1, 145.1, 137.1, 133.1, 131.1, 123.1, 119.1, 109.1, 107.1, 105.1 |
| EF-069 | fischeriolide B | ent-abietane diterpene | C_20_H_26_O_4_ | 10.03 | 331.1903 | -0.2 | 313.2, 303.2, 295.2, 285.2, 271.2, 267.2, 257.2, 239.2, 229.1, 215.1, 211.1, 203.1, 193.0, 189.1, 175.0, 165.1, 155.1, 147.0, 137.1, 131.1, 125.0, 119.1, 105.1 |
| EF-070 | fischeriolide D | ent-abietane diterpene | C_20_H_26_O_4_ | 10.77 | 331.1889 | -4.5 | 313.2, 299.2, 285.2, 281.2, 271.2, 267.2, 257.2, 253.2, 249.2, 243.1, 237.1, 229.1, 225.1, 215.1, 211.1, 203.1, 197.1, 193.0, 183.1, 169.1, 161.1, 157.1, 153.1, 145.1, 133.1, 123.1, 119.1, 105.1 |
| EF-071 | eupholide G | ent-abietane diterpene | C_20_H_26_O_3_ | 11.48 | 315.19547 | -1.7 | 297.2, 287.2, 279.2, 269.2, 259.1, 251.2, 241.2, 229.1, 227.1, 213.1, 199.1, 195.1, 185.1, 177.1, 1731.1, 165.1, 157.1, 149.1, 143.1, 137.0, 131.1, 123.1, 119.1, 107.1, 105.1 |
| EF-072 | ent-11β-hydroxyabieta-8(14),13(15)-dien-16,12β-olide | ent-abietane diterpene | C_20_H_28_O_3_ | 11.65 | 317.2098 | -4.3 | 299.2, 281.2, 271.2, 253.2, 243.1, 235.1, 229.1, 223.1, 215.1, 203.1, 197.1, 183.1, 175.1, 169.1, 161.1, 157.1, 152.1, 145.1, 141.1, 133.1, 123.1, 119.1, 105.1 |
| EF-073 | 7β,11β,12β-trihydroxy-ent-abieta-8(14),13(15)-dien-16,12-olide | ent-abietane diterpene | C_20_H_28_O_5_ | 9.24 | 349.2005 | -1.2 | 331.2, 313.2, 295.2, 285.2, 271.2, 257.3, 231.1, 217.1, 189.1, 175.0, 161.1, 151.1 147.0, 133.1, 125.1, 119.0, 105.1 |
| EF-074 | 8β,14α-dihydroxyabiet-13(15)-ene-16,12-lactone | ent-abietane diterpene | C_20_H_30_O_4_ | 8.69 | 335.2214 | -1.0 | 317.2, 299.2, 292.2, 287.2, 275.2, 271.2, 261.1, 247.2, 241.2, 229.2, 227.1, 217.2, 211.1, 201.1, 197.1, 185.1, 179.1, 173.1, 165.1, 159.1, 149.1, 143.1, 133.1, 143.1, 133.1, 128.1, 121.1, 117.1, 105.1 |
| EF-075 | 11β-hydroxy-8,14-epoxy-ent-abieta-13(15)-en-16,12-olide | ent-abietane diterpene | C_20_H_28_O_4_ | 9.41 | 333.2053 | -2.1 | 315.2, 297.2, 287.2, 279.2, 273.2, 269.2, 255.2, 245.1, 239.1, 227.1, 223.1, 217.1, 213.1, 201.1, 199.1, 187.1, 185.1, 177.1, 175.1, 173.1, 171.1, 157.1, 151.1, 149.1, 135.1, 131.1, 121.1, 117.1, 107.1, 105.1 |
| EF-076 | (5R,8S,9R,10R,11R,12R,14R)-11-hydroxy-12-methoxy-8,14-epoxyabieta-13(15)-en-16,12-olide | ent-abietane diterpene | C_21_H_30_O_5_ | 10.16 | 363.2163 | -0.7 | 345.2, 331.2, 317.2, 313.2, 295.2, 285.2, 271.2, 267.2, 257.1, 247.1, 239.1, 229.1, 225.1, 215.1, 199.1, 191.1, 177.1, 175.1, 173.1, 161.1, 147.1, 133.1, 123.1, 109.1, 107.1, 105.1 |
| EF-077 | euphopilolide | ent-abietane diterpene | C_20_H_28_O_3_ | 11.22 | 317.2108 | -0.9 | 299.2, 289.2, 285.2, 281.2, 271.2, 257.2, 253.2, 239.2, 229.1, 225.1, 211.1, 203.1, 197.1, 187.1, 179.1, 175.0, 161.1, 157.1, 147.0, 141.1, 135.1, 129.1, 123.1, 117.1, 105.1 |
| EF-078 | langduin C | ent-abietane diterpene | C_20_H_30_O_5_ | 9.31 | 351.2161 | -1.3 | 333.2, 315.2, 297.2, 287.2, 269.2, 251.2, 241.2, 233.1, 209.1, 195.0, 191.1, 177.1, 175.1, 161.1, 153.1, 145.1, 137.1, 123.1, 109.1, 105.1 |
| EF-079 | yuexiandajisu D | ent-abietane diterpene | C_20_H_30_O_5_ | 8.50 | 351.2165 | -0.3 | 333.2, 315.2, 297.2, 287.2, 279.2, 269.2, 259.2, 241.2, 227.1, 213.1, 205.1, 199.1, 185.1, 177.1, 171.1, 161.1, 149,.1, 143.1, 133.1, 121.1, 107.1 |
| EF-080 | eupholide A | ent-abietane diterpene | - | - | - | - | - |
| EF-081 | eupholide B | ent-abietane diterpene | C_22_H_34_O_7_ | 8.92 | 411.2383 | 1.3 | 375.2, 347.2, 333.2, 319.1, 313.1, 297.1, 277.2, 273.0, 259.2, 241.2, 235.2, 231.2, 221.2, 213.2, 195.1, 185.1, 171.1, 159.1, 149.1, 135.1, 133.1, 121.1, 119.1, 107.1, 105.1 |
| EF-082 | ent-8α,14β-ldihydroxy-l3 (15)-ene-16 (12α)-abietanolide | ent-abietane diterpene | C_20_H_30_O_4_ | 9.78 | 335.2185 | -9.7 | 317.2, 299.2, 275.2, 263.1, 255.2, 247.2, 227.2, 217.1, 201.2, 133.1, 123.1 |
| EF-083 | yuexiandajisu E | ent-abietane diterpene | C_20_H_28_O_5_ | 10.03 | 349.2007 | -0.8 | 331.2, 313.2, 303.2, 295.2, 285.2, 271.2, 267.2, 257.1, 243.1, 231.1, 229.1, |
| EF-084 | (4R,4aR)-dihydroxy-3-hydroxymethyl-7,7,10a-trimethyl-2,4,4a,5,6,6a,7,8,9,10,10a,l0b-dodecahydrophenanthro[3,2-b]furan-2-one | ent-abietane diterpene | C_20_H_28_O_5_ | 10.32 | 349.1994 | -4.6 | 331.2, 317.2, 303.2, 299.2, 289.2, 285.2, 271.2, 249.1, 243.1,225.1, 221.1, 207.1, 201.1, 189.1, 177.1, 175.0, 161.1, 147.1, 137.0, 123.1, 117.1, 107.1 |
| EF-085 | euphonoid A | ent-abietane diterpene | C_21_H_30_O_4_ | 13.38 | 347.2220 | 0.8 | 315.2, 297.2, 287.2, 269.2, 259.2, 251.2, 241.1, 227.1, 215.1, 213.1, 201.1, 187.1, 177.1, 175.1, 173.1, 161.1, 149.1, 133.1, 123.1, 119.1, 109.1 |
| EF-086 | eupholide F | ent-abietane diterpene | C_20_H_26_O_5_ | 11.73 | 347.1845 | -2.4 | 329.2, 315.2, 311.2, 301.2, 297.2, 283.2, 273.2, 269.2, 259.1, 255.2, 241.1, 237.2, 227.1, 223.1, 213.1, 205.1, 199.1, 191.0, 187.1, 181.1, 177.1, 173.1, 167.1, 163.1, 159.1, 157.1, 145.1, 143.1, 133.1, 123.0, 119.1, 115.1, 109.1, 107.1, 105.1 |
| EF-087 | fischeriolide A | ent-abietane diterpene | C_20_H_28_O_4_ | 10.16 | 333.2050 | -3.1 | 315.2, 297.2, 287.2, 279.2, 269.2, 255.2, 251.2, 245.2, 231.1, 227.1, 213.1, 209.1, 199.1, 185.1, 171.1, 163.1, 157.1, 143.1, 129.1, 123.1, 117.1, 107.1 |
| EF-088 | 17 hydroxy,11a, 8(14) epoxy-ent-abieta-13(15)-ene-11,12-dioxide | ent-abietane diterpene | C_21_H_28_O_6_ | 11.15 | 377.1953 | -1.4 | 359.2, 345.2, 345.2, 327.2, 317.2, 313.2, 299.2, 289.2, 281.2, 257.2, 255.2, 247.1, 241.2, 235.1, 225.2, 217.1, 211.1, 193.1, 189.0, 179.1, 177.1, 175.1, 157.1, 147.0, 133.1, 121.1, 119.1, 109.1 |
| EF-089 | fischernolide A | ent-abietane diterpene | C_29_H_34_O_9_ | 12.12 | 527.2276 | 0.0 | 509.2, 491.2, 473.2, 465.2, 463.2, 445.2, 437.2, 409.1, 393.1, 385.1, 371.1, 367.1, 353.1, 341.1, 327.1, 325.1, 309.1, 281.1, 247.1, 195.1 |
| EF-090 | fischernolide B | ent-abietane diterpene | C_29_H_34_O_9_ | 12.38 | 527.2279 | -0.6 | 509.2, 491.2, 481.2, 473.2, 463.2, 445.2, 371.1, 353.1, 343.1, 327.1, 247.1, 195.1, 183.1, 177.1 |
| EF-091 | fischernolide C | ent-abietane diterpene | C_30_H_36_O_9_ | 13.82 | 541.2432 | 0.1 | 509.2, 491.2, 481.2, 467.2, 463.2, 435.2, 427.1, 385.1, 371.1, 353.1, 343.1, 329.1, 309.1, 301.1, 195.1, 183.1, 177.1, 153.1 |
| EF-092 | fischernolide D | ent-abietane diterpene | C_29_H_32_O_8_ | 12.79 | 509.2170 | 0.0 | 491.2, 473.2, 465.2, 463.2, 445.2, 435.2, 421.2, 407.1, 395.1, 381.1, 371.1, 359.1, 343.1, 339.1, 325.1, 315.1, 301.1, 273.1, 261.1, 247.1, 229.0, 195.1, 137.1 |
| EF-093 | euphorin H | ent-abietane diterpene | C_21_H_30_O_5_ | 11.96 | 363.2162 | -1.2 | 345.2, 331.2, 327.2, 317.2, 313.2, 299.2, 295.2, 285.2, 275.2, 271.2, 267.2, 257.2, 245.1, 239.1, 231.1, 225.2, 215.1, 207.1, 201.1, 189.1, 179.1, 175.0, 171.1, 161.1, 147.0, 133.1, 121.1, 105.1 |
| EF-094 | 13β-hydroxy-ent-abiet-8(14)-en-7-one | ent-abietane diterpene | C_20_H_32_O_2_ | 10.25 | 305.2461 | -4.7 | 287.2, 273.2, 269.2, 259.2, 245.2, 243.2, 227.2, 217.2, 215.2, 213.2, 199.1, 187.1, 173.1, 161.1, 159.1, 151.1, 149.1, 142.1, 135.1, 123.1, 121.1, 119.1, 109.1, 107.1, 105.1 |
| EF-095 | (5R,9S,10R,13R)-13-methoxyabieta-8(14)-en-7-one | ent-abietane diterpene | - | - | - | - | - |
| EF-096 | eupholide H | ent-abietane diterpene | C_20_H_32_O_3_ | 14.43 | 321.2416 | -2.6 | 303.2, 285.2, 275.2, 267.2, 257.2, 247.2, 231.2, 229.2, 217.2, 215.2, 213.2, 199.1, 187.1, 177.2, 173.1, 163.1, 159.1, 157.1, 147.1, 145.1, 142.1, 135.1, 133.1, 121.1, 119.1, 109.1, 107.1, 105.1 |
| EF-097 | fischerianoid A | ent-abietane diterpene | - | - | - | - | - |
| EF-098 | fischerianoid B | ent-abietane diterpene | C_20_H_28_O_4_ | 11.77 | 333.2052 | -2.6 | 315.2, 301.2, 297.2, 287.2, 279.2, 273.2, 271.2, 269.2, 255.2, 245.2, 241.2, 227.1, 217.1, 215.1, 213.1, 205.1, 199.1, 187.1, 162.1, 157.1, 145.1, 133.1, 119.1, 107.1 |
| EF-099 | fischerianoid C | ent-abietane diterpene | C_23_H_34_O_4_ | 14.04 | 375.2498 | -8.5 | 357.2, 339.3, 329.2, 315.2, 301.2, 297.2, 287.2, 279.2, 269.2, 255.1, 249.1, 241.1, 227.1, 211.1, 205.1, 191.1, 177.1, 175.1, 163.0, 149.1, 139.0, 135.0, 105.1 |
| EF-100 | euphonoid C | ent-abietane diterpene | C_21_H_32_O_3_ | 13.27 | 333.2414 | -3.2 | 315.2, 301.2, 287.2, 283.2, 265.2, 255.2, 241.3, 217.1, 213.2, 199.1, 193.1, 187.1, 177.1, 171.1, 163.1, 157.1, 147.1, 143.1, 135.1, 133.1, 131.1, 123.1, 121.1, 119.1, 109.1, 107.1, 105.1 |
| EF-101 | (5R,9S,10R,12R)-12-methoxyabieta-8(14),13(15)-dien-16-oic acid methylester | ent-abietane diterpene | - | - | - | - | - |
| EF-102 | methyl-8,11-3-dihydroxy-12-oxo-ent-abietadi-13,15(17)-ene-16-oate | ent-abietane diterpene | C_21_H_30_O_5_ | 8.50 | 363.2162 | -1.2 | 345.2, 331.2, 327.2, 317.2, 313.2, 303.2, 295.2, 285.2, 283.2, 271.1, 267.2, 255.2, 249.1, 243.1, 231.1, 221.1, 211.1, 207.1, 201.1, 189.1, 183.1 175.0, 171.1, 161.1, 149.1, 147.0, 143.1, 133.1, 123.1, 115.1, 109.1, 107.1, 105.1 |
| EF-103 | euphonoid D | ent-abietane diterpene | C_20_H_30_O_2_ | 13.14 | 303.2311 | -2.4 | 285.2. 267.2, 261.2, 257.2, 247.2, 229.2, 225.2, 215.2, 211.1, 201.2, 197.1, 189.1, 183.1, 175.1, 171.1, 161.1, 157.1, 147.1, 145.1, 131.1, 123.1, 119.1, 109.1, 107.1, 105.1 |
| EF-104 | raserrane B | ent-abietane diterpene | C_20_H_30_O | 13.82 | 287.2369 | -0.3 | 269.2, 231.2, 213.2, 199.1, 187.1, 173.1, 163.1, 149.1, 145.1, 133.1, 123.1, 121.1, 109.1, 107.1, 105.1 |
| EF-105 | 7-dehydroabietanone | ent-abietane diterpene | C_20_H_28_O | 16.11 | 285.2203 | -3.6 | 267.2, 243.2, 255.2, 211.1, 201.1, 197.1, 189.1, 183.1, 173.1, 169.1, 167.1, 161.1, 157.1, 155.1, 147.1, 145.1, 143.1, 141.1, 131.1, 129.1, 123.1 |
| EF-106 | abieta-8,11,13-triene | ent-abietane diterpene | C_20_H_30_ | 16.08 | 271.2411 | -3.1 | 243.2, 229.2, 215.2, 213.2, 201.2, 199.1, 189.2, 187.1, 175.1, 173.1, 163.1, 161.1, 159.1, 157.1, 149.1, 147.1, 145.1, 143.1, 137.1, 135.1, 133.1, 131.1, 129.1, 123.1, 121.1, 119.1, 117.1, 115.1, 111.1, 109.1, 107.1, 105.1 |
| EF-107 | 15-hydroxydehydroabietic acid | ent-abietane diterpene | C_20_H_28_O_3_ | 13.75 | 317.2107 | -1.3 | 299.2, 289.2, 281.2, 271.2, 257.2, 253.2, 243.1, 239.1, 229.1, 217.1, 215.1, 211.1, 201.1, 197.1, 193.1, 189.1, 183.1, 171.1, 161.1, 157.1, 145.1, 133.1, 123.1, 117.1, 107.1, 105.1 |
| EF-108 | euphorfischerin A | ent-abietane diterpene | C_18_H_24_O_3_ | 14.23 | 289.17987 | -3.7 | 271.2, 245.2, 233.2, 229.2, 215.2, 201.2, 187.1, 175.1, 171.0, 161.1, 149.1, 145.1, 137.1, 133.1, 121.1, 109.1, 107.1, 105.1 |
| EF-109 | (4αS,10αS)-1,2,3,4,4α,10α-hexahydro-1,1,4α-trimethyl-7-(1-methyl)phenanthrene | ent-abietane diterpene | C_20_H_28_ | 15.00 | 269.2256 | -3.0 | 211.1, 197.1, 185.1, 173.1, 169.1, 161.1, 157.1, 147.1, 145.1, 143.1, 131.1, 128.1, 119.1, 115.1, 109.1, 105.1 |
| EF-110 | 2-phenanthrenylethanone | ent-abietane diterpene | - | - | - | - | - |
| EF-111 | (4βS,8αS)- 2-phenanthrenecarboxylic acid-4β,5,6,7,8,8α,9,10-octahydro-3-hydroxy-4β,8,8-trimethyl-methyl ester | ent-abietane diterpene | C_19_H_26_O_3_ | 10.06 | 303.1965 | 3.3 | 285.2, 273.2, 267.2, 257.2, 243.1, 239.2, 229.2, 225.1, 213.2, 205.1, 199.1, 187.1, 18301, 173.1, 163.1, 159.1, 151.1, 147.1, 143.1, 113.1, 123.1, 119.1, 109.1, 107.1, 105.1 |
| EF-112 | 17-nor-7α-hydroxy-15-oxoabieta-8,11,13-triene | ent-abietane diterpene | - | - | - | - | - |
| EF-113 | ent-3β-hydroxy-rosa-1(10),15-diene | rosane diterpene | C_20_H_32_O | 13.21 | 289.2516 | -3.4 | 271.2, 259.2, 253.2, 245.2, 233.2, 229.2, 219.2, 215.2, 213.2, 203.2, 201.2, 193.2, 187.1, 179.1, |
| EF-114 | ent-3β,19-dihydroxy-rosa-1(10),15-diene (ebractenoid C) | rosane diterpene | C_20_H_32_O_2_ | 13.31 | 305.2469 | -1.9 | 287.2, 269.2, 259.2, 249.2, 241.2, 231.2, 229.2, 227.2, 215.2, 213.2, 201.2, 191.1, 187.1, 175.1, 167.1, 159.1, 149.1, 147.1, 145.1, 143.1, 131.1, 123.1, 121.1, 119.1, 109.1, 107.1, 105.1 |
| EF-115 | ent-2α,3β-dihydroxy-rosa-1(10),15-diene (yuexiandajisu F) | rosane diterpene | C_20_H_32_O_2_ | 12.41 | 305.2463 | -3.9 | 287.2, 269.2, 259.2, 241.2, 231.2, 229.2, 219.1, 217.2, 213.2, 213.2, 203.2, 201.2, 199.1, 187.1, 285.1, 175.1, 173.1, 171.1, 163.1, 161.1, 159.1, 151.1, 149.1, 147.1, 154.1, 139.1, 135.1, 133., 131.1,123.1, 121.1, 119.1, 109.1, 107.1, 105.1 |
| EF-116 | 3,20-dihydroxy-ent-1(10),15-rosadiene | rosane diterpene | C_20_H_32_O_2_ | 13.75 | 305.2468 | -3.9 | 287.2, 269.2, 243.2, 231.1, 217.2, 203.1, 199.1, 191.2, 185.1, 177.1, 165.1, 163.0, 159.1, 149.1, 139.1, 133.1, 123.1, 119.1, 105.1 |
| EF-117 | 3,7-dihydroxy-ent-1(10),15-rosadiene | rosane diterpene | C_20_H_32_O_2_ | 11.89 | 305.2464 | -3.6 | 287.2, 269.2, 259.2, 254.2, 245.2, 243.2, 241.2, 229.2, 226.1, 213.1, 203.1, 199.1, 191.1, 185.1, 171.1, 159.1, 151.1, 145.1, 137.1, 135.1, 133.1, 131.1, 123.1, 121.1, 119.1, 117.1, 109.1, 107.1, 105.1 |
| EF-118 | euphorin A | rosane diterpene | C_20_H_30_O_2_ | 12.09 | 303.2320 | 0.5 | 285.2, 267.2, 257.2, 243.2, 229.2, 227.2, 217.2, 211.1, 201.2, 197.1, 187.1, 173.1, 161.1, 147.0, 143.1, 133.1, 131.1, 121.1, 119.1, 109.1, 107.1, 105.1 |
| EF-119 | euphorin B | rosane diterpene | C_20_H_30_O_3_ | 11.41 | 319.2261 | -2.1 | 301.2, 283.2, 273.2, 265.2, 257.2, 241.2, 231.1, 217.2, 213.1, 203.1, 199.1, 187.1, 181.1, 177.1, 171.1, 163.1, 157.1, 149.1, 145.1, 135.1, 133.1, 131.1, 123.1, 121.1, 119.1, 107.1, 105.1 |
| EF-120 | ebractenoid H | rosane diterpene | C_20_H_30_O_3_ | 11.54 | 319.2253 | -4.5 | 301.2, 283.2, 273.2, 255.2, 245.1, 231.1, 227.2, 217.1, 213.1, 205.1, 199.1, 191.1, 185.1, 177.1, 171.1, 163.0, 159.1, 149.1, 143.1, 135.1, 123.1, 121.1, 119.1, 109.1 |
| EF-121 | yuexiandajisu F | rosane diterpene | C_20_H_32_O_2_ | 13.62 | 305.2474 | -0.3 | 287.2, 269.2, 259.2, 245.2, 231.2, 229.2, 215.1, 213.2, 203.1, 199.1, 187.0, 171.1, 163.0, 159.1, 157.1, 147.1, 135.1, 133.1, 131.1, 123.1, 121.1, 119.1, 109.1, 107.1, 105.1 |
| EF-122 | 3α,17-dihydroxy-ent-pimara-8(14),15-diene | piramane diterpene | C_20_H_32_O_2_ | 14.01 | 305.2465 | -3.4 | 287.2, 269.2. 259.2, 245.2, 241.2, 231.1, 229.2, 219.2, 213.2, 203.1, 201.2, 191.1, 187.1, 177.1, 173.1, 165.1, 163.0, 159.1, 149.1, 145.1, 135.1, 133.1, 123.1, 121.1, 119.1, 109.1, 107.1, 105.1 |
| EF-123 | isopimara-9(11),15-diene-3,19-diol | piramane diterpene | C_20_H_32_O_2_ | 14.46 | 305.2464 | -1.0 | 287.2, 269.2, 259.2, 243.2, 231.2, 229.2, 219.2, 215.2, 213.2, 205.2, 201.2, 199.1, 191.1, 189.1, 187.1, 185.1, 179.1, 173.1, 163.1, 161.1, 157.1, 149.1, 147.1, 137.1, 135.1, 133.1, 123.1, 121.1, 119.1, 109.1, 107.1 |
| EF-124 | pimara-8(14),15-diene-3α,17-diol | piramane diterpene | C_20_H_32_O_2_ | 14.62 | 305.2461 | -2.7 | 287.2, 269.2, 259.2, 257.2, 245.2, 231.2, 229.2, 219.2, 217.2, 215.2, 213.2, 205.2, 203.2, 201.2, 199.1, 191.1, 187.1, 185.1, 175.1, 173.1, 167.1, 161.1, 159.1, 151.1, 149.1, 147.1, 145.1, 143.1, 135.1, 133.1, 131.1, 123.1, 121.1,119.1, 109.1, 107.1, 105.1 |
| EF-125 | ent-8(14)-pimarene-12β,15S,16-triol | piramane diterpene | C_20_H_34_O_3_ | 10.22 | 323.2582 | 0.3 | 305.2, 291.1, 287.2, 277.2, 269.2, 263.1, 259.2, 245.1, 231.2, 221.2, 219.1, 213.2, 203.2, 199.1, 187.1, 185.1, 171.1, 161.1, 147.1, 143.1, 135.1, 129.1, 123.1, 121.1, 119.1, 109.1, 107.1, 105.1 |
| EF-126 | (3S,11R,12R)-3,11,12-trihydroxy-ent-isopimara-7,15-dien-2-one. | piramane diterpene | C_20_H_32_O_4_ | 9.24 | 337.2365 | -2.6 | 319.2, 301.2, 289.2, 283.2, 273.2, 261.2, 259.2, 255.2, 243.2, 233.2, 227.1, 223.1, 215.2, 199.1, 189.1, 185.1, 177.1, 163.1, 159.1, 145.1, 135.1, 123.1, 121.1, 109.1, 107.1, 105.1 |
| EF-127 | (3S,11R,12S)-3,11,12-trihydroxy-ent-isopimara-7,15-dien-2-one | piramane diterpene | C_20_H_32_O_4_ | 9.41 | 337.2367 | -2.6 | 319.2, 301.2, 291.2, 283.2, 273.2, 261.2, 259.2, 243.2, 231.2, 223.1, 217.2, 213.2, 205.1, 199.1, 191.1, 185.1, 175.1, 165.1, 157.1, 149.1, 137.1, 133.1, 123.1, 119.1, 109.1, 107.1, 105.1 |
| EF-128 | (3R,11R,12S)-3,11,12-trihydroxy-ent-isopimara-7,15-diene | piramane diterpene | C_20_H_32_O_3_ | 10.87 | 321.2423 | -0.2 | 285.2, 275.2, 273.2, 267.2, 257.2, 243.2, 233.2, 227.2, 215.1, 211.1, 203.1, 197.1, 187.1, 173.1, 165.1, 151.1, 147.1, 137.1, 133.1, 123.1, 119.1, 109.1, 107.1, 105.1 |
| EF-129 | (11R,12S)-11,12-dihydroxy-ent-isopimara-7,15-dien-3-one | piramane diterpene | C_20_H_30_O_3_ | 9.28 | 319.2262 | -0.3 | 301.2, 283.2, 273.2, 271.2, 259.2, 255.2, 245.2,243.2, 241.2, 225.2, 215.1, 213.1, 201.1, 191.1, 187.1, 177.1, 173.1, 159.1, 149.1, 147.1, 133.1, 131.1, 123.1, 119.1, 109.1, 107.1, 105.1 |
| EF-130 | (11R,12R)-11,12-dihydroxy-ent-isopimara-7,15-dien-3-one | piramane diterpene | C_20_H_30_O_3_ | 10.93 | 319.2270 | 0.6 | 301.2, 283.2, 277.2, 273.2, 259.2, 255.2, 245.1, 239.2, 231.1, 227.1, 217.2, 205.1, 199.1, 187.1, 185.1, 173.1, 163.1, 149.1, 145.1, 133.1, 129.1, 121.1, 107.1, 105.1 |
| EF-131 | (2S,11R,12S)-2,11,12-trihydroxy-ent-isopimara-7,15-dien-3-one | piramane diterpene | C_20_H_30_O_4_ | 7.76 | 335.2201 | -3.6 | 317.2, 299.2, 289.2, 281.2, 275.2, 271.2, 269.2, 257.2, 253.2, 239.2, 229.2, 223.2, 217.2, 213.1, 197.1, 183.1, 175.1, 169.1, 159.1, 145.1, 135.1, 131.1, 121.1, 109.1, 107.1, 105.1 |
| EF-132 | (2R,3S,11R)-2,3,11-trihydroxy-ent-isopimara-7,15-dien-12-one | piramane diterpene | C_20_H_30_O_4_ | 11.03 | 335.2215 | -0.4 | 317.2, 299.2, 289.2, 281.2, 271.2, 253.2, 241.2, 229.1, 225.2, 215.1, 203.1, 197.1, 183.1, 179.1 175.1, 161.1, 157.1, 143.1, 133.1, 119.1, 105.1 |
| EF-133 | (2R,3S)-2,3-dihydroxy-ent-isopimara-7,15-dien-12-one | piramane diterpene | C_20_H_30_O_3_ | 10.55 | 319.2259 | -2.8 | 301.2, 289.2, 283.2, 271.2, 265.2, 259.2, 243.2, 241.2, 227.2, 225.2, 203.1, 199.1, 185.1, 171.1, 163.0, 159.1, 149.1, 145.1, 133.1, 119.1, 105.1 |
| EF-134 | (3R)-3-hydroxy-ent-isopimara-7,15-dien-12-one | piramane diterpene | C_20_H_30_O_2_ | 9.47 | 303.2307 | -3.9 | 285.2, 267.2, 261.2, 247.2, 229.2, 213.2, 211.1, 201.2, 197.1, 187.1, 179.1, 175.1, 161.1, 157.1, 145.1, 133.1, 119.1, 109.1 |
| EF-135 | (3R,11R)-3,11-dihydroxy-ent-isopimara-7,15-dien-12-one | piramane diterpene | C_20_H_30_O_3_ | 11.32 | 319.2259 | -2.8 | 301.2, 283.2, 273.2, 259.2, 255.2, 243.2, 231.1, 227.1, 217.2, 203.1, 189.1, 187.1, 181.0, 175.1, 173.1, 171.1, 163.0, 157.1, 147.1, 143.1, 133.1, 123.1, 119.1, 109.1, 107.1, 105.1 |
| EF-136 | (3β,13α)-3-hydroxypimara-7,15-dien-2,12-dione | piramane diterpene | C_20_H_28_O_3_ | 13.18 | 317.2102 | -2.7 | 299.2, 281.2, 271.2, 257.2, 253.2, 243.1, 229.1, 217.1, 215.1, 211.1, 201.1, 197.1, 193.1, 189.1, 183.1, 175.1, 161.1, 157.1, 145.1, 137.1, 123.1, 117.1, 109.1, 105.1 |
| EF-137 | (3β,12α,13α)-3,12-dihydroxypimara-7,15-dien-2-one | piramane diterpene | C_20_H_30_O_3_ | 12.63 | 319.2252 | -2.8 | 301.2, 283.2, 273.2, 259.2, 255.2, 245.2, 231.2, 225.2, 219.1, 217.2, 199.1, 191.1, 189.1, 175.1, 173.1, 171.1, 159.1, 149.1, 145.1, 135.1, 123.1, 117.1, 109.1, 107.1, 105.1 |
| EF-138 | (3β,12β)-3,12-dihydroxyisopimara-7,15-dien-2-one | piramane diterpene | C_20_H_30_O_3_ | 14.78 | 319.2266 | -0.4 | 301.2, 283.2, 273.2, 259.2, 249.2, 231.2, 225.2, 213.1, 199.1, 187.1, 181.0, 175.1, 169.1, 163.1, 151.1, 147.1, 141.1, 133.1, 12.1, 121.1, 117.1, 107.1 |
| EF-139 | (11R,12R)-2,11,12-trihydroxy-ent-isopimara-1,7,15-trien-3-one | piramane diterpene | C_20_H_30_O_4_ | 10.26 | 335.2204 | -8.7 | 317.2, 299.2, 289.2, 275.2, 271.2, 261.1, 253.2, 243.2, 229.2, 215.1, 211.1, 201.1, 189.1, 187.1, 185.1, 175.1, 165.1, 147.0, 143.1, 123.1, 121.1, 119.1, 109.1, 107.1, 105.1 |
| EF-140 | (11R,12S)-2,11,12-trihydroxy-ent-isopimara-1,7,15-trien-3-one | piramane diterpene | C_20_H_30_O_4_ | 10.58 | 335.2188 | -3.5 | 317.2, 299.2, 289.2, 281.2, 271.2, 253.2, 243.1, 229.1, 225.2, 217.1, 201.1, 197.1, 183.1, 179.1, 175.1 169.1, 161.1, 155.1, 147.1, 133.1, 131.1, 121.1, 119.1, 109.1, 105.1 |
| EF-141 | isopimara-7,15-dien-3-one | piramane diterpene | C_20_H_30_O | 11.50 | 287.2363 | -2.3 | 269.2, 231.2, 227.2, 213.2, 211.1, 201.2, 199.1, 187.1, 185.1, 175.1, 173.1, 171.1, 163.1, 161.1, 159.1, 157.1, 149.1, 147.1, 145.1, 143.1, 137.1, 135.1, 133.1, 131.1, 123.1, 121.1, 119.1, 117.1, 109.1, 107.1, 105.1 |
| EF-142 | 2-hydroxy-ent-isopimara-1,7,15-trien-3,12-dione | piramane diterpene | C_20_H_26_O_3_ | 13.33 | 315.1953 | -0.5 | 297.2, 269.2, 259.2, 255.1, 245.1, 241.1, 227.1, 215.1, 203.1, 191.1, 187.1, 177.1, 175.1, 173.1, 161.1, 149.1, 145.1, 133.1, 121.1, 115.1, 105.1 |
| EF-143 | (12β)-2,12-dihydroxy isopimara-1,7,15-trien-3-one | piramane diterpene | C_20_H_28_O_3_ | 12.76 | 317.2111 | -3.7 | 299.2, 281.2, 271.2, 253.2, 243.2, 233.1, 229.1, 221.1, 217.1, 211.1, 201.1, 189.1, 179.0, 175.1, 159.1, 145.1, 133.1, 119.1, 105.1 |
| EF-144 | yuexiandajisu C | piramane diterpene | C_20_H_28_O_3_ | 12.44 | 317.2101 | -3.1 | 299.2, 281.2, 271.2, 253.2, 243.2, 229.1, 217.1, 203.1, 197.1, 189.1, 175.1, 173.1, 165.1, 163.1, 161.1, 147.1, 137.1, 133.1, 119.1, 105.1 |
| EF-145 | isopimara-8(14),15-diene-3β,11α-diol | piramane diterpene | C_20_H_32_O_2_ | 11.19 | 305.2461 | -2.4 | 287.2, 273.2, 269.2, 259.2, 245.2, 231.2, 227.2, 213.2, 203.1, 201.1, 199.1, 191.1, 185.1, 177.2, 171.1, 161.1, 151.1, 149.1, 145.1, 143.1, 135.1, 131.1, 121.1, 119.1, 109.1, 107.1, 105.1 |
| EF-146 | araucarol | piramane diterpene | C_20_H_32_O_3_ | 12.79 | 321.2418 | -1.9 | 303.2, 285.2, 275.2, 267.2, 257.2, 247.2, 237.1, 229.2, 219.1, 215.1, 203.1, 189.2, 187.1, 175.1, 173.1, 163.0, 159.1, 149.1, 145.1, 135.1, 133.1, 125.1, 121.1, 119.1, 109.1, 107.1, 105.1 |
| EF-147 | araucarone | piramane diterpene | C_20_H_30_O_3_ | 15.20 | 319.2264 | -1.1 | 301.2, 289.2, 283.2, 273.2, 259.2, 255.2, 245.2, 231.2, 225.2, 223.1, 213.2, 203.1, 199.1, 189.1, 185.1, 173.1, 157.1, 149.1, 145.1, 143.1, 131.1, 119.1, 115.1, 105.1 |
| EF-148 | ent-1β,3β,16β,17-tetrahydroxyatisane | ent-atisane diterpene | C_20_H_34_O_4_ | 12.06 | 339.2524 | -1.7 | 303.2, 285.2, 231.2, 215.2, 213.2, 211.2, 197.1, 187.1, 173.1, 159.1, 145.1, 131.1, 119.1, 109.1, 107.1, 105.1 |
| EF-149 | ent-1β,3α,16β,17-tetrahydroxyatisane | ent-atisane diterpene | - | - | - | - | - |
| EF-150 | ent-atisane-3β,16α,17-triol | ent-atisane diterpene | C_20_H_34_O_3_ | 11.57 | 323.2583 | 0.6 | 305.2, 291.1, 287.2, 275.1, 269.2, 263.2, 245.2, 229.2, 217.2, 213.2, 201.2, 199.1, 187.1, 185.1, 173.1, 171.1, 161.1, 149.1, 145.1, 135.1, 133.1, 123.1, 121.1, 119.1, 109.1, 107.1, 105.1 |
| EF-151 | alboatisin A | ent-atisane diterpene | C_20_H_30_O_4_ | 8.50 | 335.2217 | -1.9 | 317.2, 299.2, 289.2, 281.2, 273.2, 271.2, 259.2, 253.2, 243.2, 235.1, 231.2, 229.1, 219.1, 213.2, 201.1, 197.1, 197.0, 191.1, 187.1, 173.1, 163.1, 159.1, 147.1, 133.1, 123.1, 119.1, 109.1, 107.1, 105.1 |
| EF-152 | ent-3β-hydroxyatis-16-ene-14-one | ent-atisane diterpene | C_20_H_30_O_2_ | 9.67 | 303.2315 | -1.2 | 285.2, 267.2, 261.2, 247.2, 243.2, 231.2, 229.2, 215.1, 211.1, 205.2, 201.2, 197.1, 187.1, 179.1, 175.1, 165.1, 161.1, 159.1, 147.1, 133.1, 121.1, 119.1, 117.1, 109.1, 107.1, 105.1 |
| EF-153 | ent-3β-hydroxyatis-16-ene-2,14-dione | ent-atisane diterpene | C_20_H_28_O_3_ | 13.05 | 317.2096 | -4.9 | 299.2, 289.2, 281.2, 271.2, 253.2, 243.2, 229.1, 225.1, 215.1, 197.1, 189.1, 183.1, 169.1, 161.1, 147.1, 133.1, 123.1, 109.1, 107.1, 105.1 |
| EF-154 | euphonoid E | ent-atisane diterpene | C_20_H_32_O_2_ | 11.44 | 305.2474 | -0.4 | 287.2, 269.2, 259.2, 245.2, 241.2, 235.2, 231.2, 217.2, 215.2, 213.2, 203.1, 201.2, 199.1, 187.1, 175.1, 173.1, 159.1, 149.1, 145.1, 135.1, 133.1, 123.1, 121.1, 119.1, 109.1, 107.1, 105.1 |
| EF-155 | euphonoid F | ent-atisane diterpene | C_20_H_26_O_3_ | 10.25 | 315.1954 | -0.2 | 297.2, 287.2, 279.2, 273.2, 269.2, 259.2, 255.2, 251.2, 241.1, 239.1, 237.1, 227.1, 221.1, 213.2, 209.1, 199.1, 195.1, 187.1, 185.1, 179.1, 173.1, 171.1, 169.1, 165.1, 159.1, 157.1, 145.1, 143.1, 133.1, 119.1, 107.1, 105.1 |
| EF-156 | ent-13α-hydroxyatis-16-ene-3,14-dione | ent-atisane diterpene | C_20_H_28_O_3_ | 8.11 | 317.2102 | -3.3 | 299.2, 289.2, 281.2, 271.2, 257.2, 253.2, 243.2, 241.2, 239.2, 229.1, 225.1, 217.1, 215.1, 211.1, 199.1, 197.1, 193.1, 189.1, 187.1, 183.1, 173.1, 169.1, 161.1, 159.1, 157.1, 155.1, 145.1, 143.1, 135.1, 133.1, 131.1, 131.1, 119.1, 117.1, 109.1, 107.1, 105.1 |
| EF-157 | ent-(13R,14R)-13,14-dihydroxyatis-16-en-3-one | ent-atisane diterpene | C_20_H_30_O_3_ | 7.60 | 319.2259 | -2.6 | 301.2, 283.2, 265.2, 259.2, 241.2, 225.2, 213.2, 203.1, 199.1, 189.1, 185.1, 177.1, 161.1, 147.1, 143.1, 133.1, 133.1, 123.1, 119.1, 109.1, 107.1, 105.1 |
| EF-158 | ent-3β,13α-dihydroxyatis-16-ene-14-one | ent-atisane diterpene | C_20_H_30_O_3_ | 7.80 | 319.2258 | -1.9 | 301.2, 283.2, 273.2, 265.2, 259.2, 245.2, 241.2, 231.2, 227.1, 225.2, 223.1, 217.2, 215.1, 211.1, 209.1, 203.1, 199.1, 195.1, 189.1, 187.1, 185.1, 183.1, 175.1, 171.1, 163.1, 161.1, 157.1, 155.1, 149.1, 147.1, 145.1, 135.1, 133.1, 131.1, 123.1, 121.1, 119.1, 109.1, 107.1, 105.1 |
| EF-159 | ent-atis-16-ene-3,14-dione | ent-atisane diterpene | C_20_H_28_O_2_ | 9.90 | 301.2163 | 0.4 | 283.2, 273.2, 271.2, 255.2, 241.2, 227.2, 225.2, 213.1, 209.1, 199.1, 195.1, 187.1, 185.1, 177.1, 173.1, 171.1, 163.1, 161.1, 147.1, 145.1, 143.1, 135.1, 131.1, 123.1, 119.1, 107.0, 105.1 |
| EF-160 | ent-16α,17-dihydroxy-atisan-3-one | ent-atisane diterpene | C_20_H_32_O_3_ | 8.89 | 321.2436 | 3.6 | 303.2, 285.2, 273.2, 267.2, 261.1, 255.2, 243.2, 227.2, 219.1, 211.1, 201.2, 197.1, 187.1, 1831, 173.1, 164.1, 159.1, 145.1, 133.1, 131.1, 129.1, 123.1, 121.1, 119.1, 117.1, 109.1, 107.1, 105.1 |
| EF-161 | ent-kaur-16-en-14-ol | ent-kaurane diterpene | C_20_H_32_O | 9.44 | 289.2518 | -2.7 | 271.2, 261.2, 253.2, 243.2, 233.2, 229.2, 215.2, 201.2, 191.1, 189.1, 187.1, 175.1, 173.1, 163.1, 159.1, 149.1, 135.1, 131.1, 121.1, 119.1, 109.1, 107.1, 105.1 |
| EF-162 | ent-kaurane-3-oxo-16α,17-diol | ent-kaurane diterpene | - | - | - | - | - |
| EF-163 | ent-kaurane-3-oxo-17β-ol | ent-kaurane diterpene | C_20_H_32_O_2_ | 7.66 | 305.2464 | -3.5 | 287.2, 269.2, 257.2, 241.2, 227.2, 217.2, 215.2, 213.2, 203.2, 201.2, 199.1, 187.1, 185.1, 179.1, 177.1, 175.1, 173.1, 171.1, 161.1, 159.1, 157.1, 149.1, 147.1, 145.1, 143.1, 133.1, 129.1, 121.1, 119.1, 109.1, 107.1, 105.1 |
| EF-164 | ent-16β-H-3-oxo-kauran-17-ol | ent-kaurane diterpene | C_20_H_32_O_3_ | 8.11 | 321.2415 | -2.9 | 303.2, 285.2, 267.2, 257.2, 243.2, 239.2, 229.2, 227.2, 221.2, 215.1, 213.1, 211.1, 201.2, 199.1, 197.1, 187.1, 185.1, 183.1, 173.1, 171.1, 169.1, 159.1, 157.1, 147.1, 145.1, 143.1, 131.1, 117.1, 109.1, 107.1, 105.1 |
| EF-165 | 3S,16S,17-trihydroxy-2-one-ent-kaurane | ent-kaurane diterpene | C_20_H_32_O_4_ | 7.73 | 337.2358 | -4.6 | 319.2, 301.2, 291.2, 283.2, 277.2, 273.2, 259.2, 255.2, 241.1, 233.2, 227.1, 215.1, 213.2, 203.1, 199.1, 187.1, 173.1, 159.1, 145.1, 133.1, 123.1, 121.1, 109.1, 107.1, 105.1 |
| EF-166 | ent-kaurane-3β,16β,17-trio1 | ent-kaurane diterpene | C_20_H_34_O_3_ | 7.82 | 323.2571 | -3.2 | 305.2, 291.1, 287.2, 269.2, 259.2, 245.2, 229.2, 213.2, 201.1, 199.1, 189.2, 187.1, 185.1, 175.1, 173.1, 163.1, 159.1, 157.1, 149.1, 139.1, 135.1, 133.1, 123.1, 121.1, 119.1, 117.1, 109.1, 107.1, 105.1 |
| EF-167 | ent-kaurane-3-oxo-16β, 17-acetonide | ent-kaurane diterpene | - | - | - | - | - |
| EF-168 | euphonoid G | ent-kaurane diterpene | C_20_H_32_O_2_ | 8.33 | 305.2468 | -2.4 | 287.2, 269.2, 259.2, 255.2, 241.2, 229.2, 215.2, 213.2, 203.2, 201.2, 189.2, 187.1, 185.1, 175.1, 173.1, 171.1, 161.1, 159.1, 157.1, 145.1, 143.1, 135.1, 133.1, 127.1, 119.1, 117.1, 109.1, 107.1, 105.1 |
| EF-169 | 3α-hydroxy-ent-16-kauren | ent-kaurane diterpene | C_20_H_30_O | 8.43 | 287.2361 | -3.0 | 269.2, 241.2, 231.2, 227.2, 217.2, 213.2, 201.2, 199.1, 189.2, 187.1, 185.1, 175.1, 173.1, 171.1, 161.1, 159.1, 147.1, 145.1, 143.1, 135.1, 133.1, 131.1, 123.1, 121.1, 117.1, 111.1, 109.1, 107.1, 105.1 |
| EF-170 | ent-2,3-dihydroxy-18-nor-rosa-1,3,5(10),15-tetraene | norrostane diterpene | C_19_H_22_O_4_ | 7.63 | 315.1586 | -1.7 | 297.2, 287.2, 279.2, 269.2, 255.2, 241.2, 237.1, 229.1, 227.1, 213.1, 211.1, 207.1, 203.1, 199.1, 195.1, 191.1, 187.1, 177.1, 171.1, 153.0, 149.1, 145.1, 133.1, 123.1, 121.1, 115.1, 107.1, 105.1 |
| EF-171 | (8S,9S,13S)-3-hydroxy-18-norrosa-1,3,5(10),15-tetraene | norrostane diterpene | C_19_H_26_O | 15.72 | 271.2061 | 1.7 | 253.2, 243.2, 227.2, 215.2, 213.2, 201.2, 199.1, 187.1, 185.1, 175.1, 173.1, 171.1, 161.1, 159.1, 147.1, 145.1, 135.1, 133.1, 131.1, 129.1, 123.1, 121.1, 119.1, 117.1, 109.1, 107.1, 105.1 |
| EF-172 | ebractenoid F | norrostane diterpene | - | - | - | - | - |
| EF-173 | jolkinol A | lathyrane diterpene | C_29_H_36_O_6_ | 10.25 | 481.2567 | -3.7 | 303.2, 297.2, 287.2, 279.2, 269.2, 257.2, 255.1, 251.2, 241.1, 239.1, 237.1, 227.1, 217.2, 215.1, 213.1, 209.1, 203.1, 199.1, 195.1, 183.1, 171.1, 157.1, 145.1, 133.1, 131.1, 119.1, 107.1, 105.1, 103.1 |
| EF-174 | langduin C | dimeric diterpene | C_40_H_50_O_10_ | 15.24 | 691.3498 | 3.1 | 673.3, 655.3, 637.3, 627.3, 609.3, 583.3, 550.5, 517.2, 511.2, 499.2, 481.2, 455.2, 437.2, 415.2, 395.1, 381.1, 371.2, 361.2, 345.2, 327.2, 317.2, 299.2, 283.2, 269.0, 241.2, 207.0, 187.1, 173.1, 149.1, 123.1 |
| EF-175 | fischeriana A | other diterpene | C_27_H_30_O_8_ | 11.73 | 483.2009 | -0.9 | 465.2, 447.2, 439.2, 421.2, 411.2, 403.2, 393.2, 323.1, 315.1, 301.1, 269.1, 257.1, 247.1, 235.1, 219.1, 205.0, 175.0, 163.1, 153.0, 119.1, 107.1 |
| EF-176 | langduin D | other diterpene | C_29_H_32_O_9_ | 11.80 | 525.2114 | -0.9 | 507.2, 489.2, 481.2, 479.2, 463.2, 453.2, 435.2, 417.2, 371.1, 353.1, 339.1, 327.1, 315.1, 297.1, 277.1, 261.1, 247.1, 229.0, 219.1, 205.1, 195.1, 163.1, 121.1, 107.1 |
| EF-177 | decandrol A | other diterpene | C_20_H_30_O_3_ | 15.59 | 319.22677 | -1.9 | 301.2, 273.2, 259.2, 255.2, 247.2, 241.2, 235.1, 231.1, 223.1, 213.2, 201.1, 187.1, 185.1, 177.1, 157.1, 149.1, 145.1, 137.1, 133.1, 123.1, 121.1, 107.1, 105.1 |

“-” means the compound was not be detected from 70% ethanol fraction

Table S2 The results of molecular docking

| No. | MAPK1 | | MAPK8 | | MAPK10 | | MAPK14 | | CDK2 | | CASP3 | |
| --- | --- | --- | --- | --- | --- | --- | --- | --- | --- | --- | --- | --- |
|  | Score | PDB | Score | PDB | Score | PDB | Score | PDB | Score | PDB | Score | PDB |
| EF-001 | 4.46 | 1PME | 4.40 | 2NO3 | 4.78 | 3CGF | 5.55 | 1W84 | 5.63 | 1JVP | 3.77 | 3DEJ |
| EF-002 | 5.43 | 1PME | 7.09 | 2G01 | 8.01 | 1PMV | 7.25 | 2ZAZ | 7.86 | 2R3F | 5.62 | 3DEJ |
| EF-003 | 5.47 | 1PME | 5.63 | 2G01 | 3.75 | 1PMV | 4.34 | 2ZAZ | 6.24 | 2UZN | 4.08 | 3DEJ |
| EF-004 | 6.05 | 1PME | 5.64 | 2NO3 | 5.44 | 3CGF | 2.99 | 1ZYJ | 5.48 | 2B53 | 4.51 | 3DEJ |
| EF-005 | 5.57 | 2OJI | 6.28 | 2G01 | 8.00 | 3CGF | 6.68 | 1DI9 | 7.90 | 2B53 | 5.89 | 3DEJ |
| EF-006 | 6.11 | 2OJI | 4.47 | 2NO3 | 4.14 | 3CGF | 5.39 | 1W84 | 5.74 | 2R3F | 4.56 | 3DEJ |
| EF-007 | 4.46 | 2OJI | 4.48 | 2NO3 | 4.10 | 3CGF | 5.17 | 1W84 | 4.76 | 2R3F | 3.98 | 3DEJ |
| EF-008 | 4.02 | 2OJI | 5.41 | 1UKI | 4.60 | 3CGF | 4.21 | 1W7H | 3.55 | 2UZN | 3.34 | 1GFW |
| EF-009 | 4.66 | 2OJI | 4.96 | 2G01 | 5.09 | 3CGF | 4.82 | 1DI9 | 3.49 | 1JVP | 3.73 | 3DEJ |
| EF-010 | 3.68 | 2OJI | 5.17 | 2NO3 | 4.21 | 3CGF | 4.69 | 2ZAZ | 4.10 | 2B53 | 2.76 | 1GFW |
| EF-011 | 7.75 | 2OJI | 8.93 | 2NO3 | 5.20 | 3CGF | 8.65 | 1ZYJ | 8.99 | 2UZN | 9.86 | 3DEJ |
| EF-012 | 4.52 | 2OJI | 4.84 | 2G01 | 3.97 | 3CGF | 5.22 | 2ZAZ | 5.48 | 1GIH | 4.60 | 3DEJ |
| EF-013 | 7.67 | 1PME | 7.38 | 1UKI | 5.16 | 3CGF | 8.96 | 1ZYJ | 9.49 | 2B53 | 7.25 | 3DEJ |
| EF-014 | 6.87 | 1PME | 5.80 | 2G01 | 7.32 | 3CGF | 9.67 | 1ZYJ | 8.74 | 2B53 | 7.29 | 3DEJ |
| EF-015 | 6.25 | 1PME | 8.26 | 2G01 | 9.33 | 3CGF | 7.42 | 1DI9 | 9.40 | 1PXI | 7.14 | 3DEJ |
| EF-016 | 6.72 | 2OJI | 6.71 | 2NO3 | 6.66 | 3CGF | 6.67 | 1ZYJ | 9.93 | 1GIH | 5.96 | 3DEJ |
| EF-017 | 3.88 | 1PME | 3.57 | 1UKI | 2.60 | 3CGF | 5.51 | 2ZAZ | 5.88 | 1GIH | 3.13 | 3DEJ |
| EF-018 | 4.46 | 1PME | 3.65 | 2NO3 | 3.87 | 3CGF | 6.94 | 1W7H | 3.98 | 2B53 | 4.79 | 3DEJ |
| EF-019 | 4.17 | 2OJI | 4.52 | 2G01 | 3.71 | 3CGF | 4.43 | 1W7H | 6.34 | 2B53 | 3.62 | 3DEJ |
| EF-020 | 4.32 | 2OJI | 5.19 | 2NO3 | 4.86 | 3CGF | 5.31 | 1DI9 | 5.19 | 2B53 | 4.47 | 3DEJ |
| EF-021 | 6.09 | 2OJI | 9.27 | 2G01 | 6.14 | 3CGF | 8.26 | 1ZYJ | 7.02 | 2UZN | 9.75 | 3DEJ |
| EF-022 | 3.58 | 2OJI | 4.88 | 2NO3 | 5.28 | 3CGF | 5.05 | 1DI9 | 5.12 | 2UZN | 3.22 | 3DEJ |
| EF-023 | 3.38 | 2OJI | 4.22 | 2NO3 | 4.70 | 3CGF | 4.34 | 1W84 | 6.75 | 2B53 | 4.91 | 1GFW |
| EF-024 | 9.62 | 1PME | 7.20 | 1UKI | 7.80 | 3CGF | 9.18 | 1ZYJ | 7.02 | 1GIH | 7.60 | 3DEJ |
| EF-025 | 4.23 | 2OJI | 5.52 | 2NO3 | 4.06 | 3CGF | 4.99 | 1DI9 | 5.80 | 2UZN | 4.16 | 3DEJ |
| EF-026 | 10.44 | 2OJI | 10.99 | 2NO3 | 10.08 | 3CGF | 8.32 | 1W84 | 9.65 | 1GIH | 9.31 | 3DEJ |
| EF-027 | 4.64 | 2OJI | 4.47 | 2NO3 | 4.89 | 3CGF | 5.56 | 1W84 | 6.21 | 1GIH | 6.25 | 1GFW |
| EF-028 | 3.84 | 1PME | 3.89 | 2NO3 | 3.16 | 3CGF | 5.03 | 1W7H | 5.30 | 2B53 | 3.38 | 1GFW |
| EF-029 | 10.46 | 1PME | 6.74 | 1UKI | 8.87 | 3CGF | 6.86 | 1ZYJ | 8.14 | 2B53 | 6.55 | 3DEJ |
| EF-030 | 9.73 | 1PME | 6.20 | 1UKI | 7.65 | 3CGF | 9.27 | 1DI9 | 7.77 | 1PXI | 9.90 | 3DEJ |
| EF-031 | 9.13 | 2OJI | 5.32 | 1UKI | 7.91 | 3CGF | 6.60 | 1ZYJ | 9.63 | 2UZN | 4.98 | 3DEJ |
| EF-032 | 7.37 | 2OJI | 7.32 | 2NO3 | 4.67 | 3CGF | 7.20 | 1DI9 | 7.00 | 1GIH | 7.11 | 3DEJ |
| EF-033 | 6.36 | 2OJI | 8.06 | 1UKI | 8.73 | 3CGF | 6.88 | 1W84 | 10.45 | 1GIH | 6.86 | 3DEJ |
| EF-034 | 4.78 | 2OJI | 4.16 | 2G01 | 5.10 | 3CGF | 4.58 | 1W84 | 4.95 | 2UZN | 4.20 | 3DEJ |
| EF-035 | 5.31 | 2OJI | 6.84 | 2G01 | 6.02 | 3CGF | 6.15 | 2ZAZ | 6.72 | 2B53 | 5.25 | 3DEJ |
| EF-036 | 4.51 | 1PME | 5.69 | 2G01 | 3.99 | 3CGF | 4.11 | 1DI9 | 2.90 | 2UZN | 5.56 | 1GFW |
| EF-037 | 8.74 | 2OJI | 7.85 | 2G01 | 8.36 | 3CGF | 7.04 | 1W84 | 8.44 | 2UZN | 5.96 | 3DEJ |
| EF-038 | 8.39 | 2OJI | 8.81 | 1UKI | 6.94 | 3CGF | 5.93 | 1DI9 | 8.69 | 2B53 | 5.63 | 1GFW |
| EF-039 | 7.62 | 2OJI | 6.98 | 1UKI | 5.72 | 3CGF | 6.00 | 2ZAZ | 7.73 | 1PXI | 5.44 | 1GFW |
| EF-040 | 6.91 | 2OJI | 6.19 | 1UKI | 4.80 | 3CGF | 5.76 | 2ZAZ | 6.70 | 2B53 | 4.61 | 1GFW |
| EF-041 | 6.54 | 2OJI | 6.40 | 2G01 | 4.91 | 1PMV | 4.53 | 1DI9 | 8.57 | 2UZN | 4.29 | 1GFW |
| EF-042 | 6.73 | 2OJI | 5.48 | 2G01 | 6.05 | 3CGF | 4.41 | 2ZAZ | 6.03 | 2UZN | 5.15 | 1GFW |
| EF-043 | 7.74 | 2OJI | 7.72 | 2NO3 | 4.88 | 3CGF | 9.47 | 1DI9 | 5.42 | 1PXI | 5.44 | 1GFW |
| EF-044 | 6.64 | 2OJI | 8.15 | 1UKI | 7.35 | 3CGF | 7.03 | 1DI9 | 8.81 | 2B53 | 5.92 | 3DEJ |
| EF-045 | 8.79 | 2OJI | 7.55 | 1UKI | 5.21 | 3CGF | 6.06 | 1DI9 | 5.55 | 2UZN | 7.68 | 1GFW |
| EF-046 | 8.72 | 2OJI | 7.82 | 1UKI | 7.34 | 3CGF | 6.59 | 1W84 | 8.91 | 2R3F | 7.60 | 3DEJ |
| EF-047 | 1.18 | 1PME | 1.63 | 2NO3 | 1.84 | 3CGF | 2.80 | 1W84 | 0.70 | 2UZN | 4.28 | 3DEJ |
| EF-048 | 1.68 | 2OJI | 3.52 | 1UKI | 3.37 | 3CGF | 4.25 | 2ZAZ | 3.60 | 2UZN | 7.89 | 1GFW |
| EF-049 | 3.43 | 2OJI | 2.89 | 2NO3 | - | 3CGF | 2.37 | 1DI9 | 2.14 | 1PXI | 5.16 | 1GFW |
| EF-050 | 3.40 | 2OJI | 2.70 | 2NO3 | 0.38 | 3CGF | 4.45 | 2ZAZ | 1.06 | 1PXI | 5.64 | 3DEJ |
| EF-051 | 4.82 | 2OJI | 4.88 | 2G01 | 4.13 | 3CGF | 7.46 | 1W7H | 5.35 | 2R3F | 3.94 | 3DEJ |
| EF-052 | 5.84 | 2OJI | 5.24 | 2G01 | 6.98 | 3CGF | 4.20 | 1DI9 | 4.56 | 2R3F | 3.67 | 1GFW |
| EF-053 | 5.68 | 2OJI | 4.48 | 2G01 | 5.45 | 3CGF | 5.55 | 1W7H | 3.70 | 2B53 | 4.34 | 1GFW |
| EF-054 | 7.39 | 2OJI | 5.42 | 2NO3 | 6.04 | 3CGF | 6.90 | 1W7H | 3.76 | 2B53 | 4.91 | 3DEJ |
| EF-055 | 4.74 | 2OJI | 5.69 | 2G01 | 6.14 | 3CGF | 4.94 | 2ZAZ | 5.39 | 2R3F | 4.26 | 3DEJ |
| EF-056 | 5.31 | 2OJI | 4.88 | 2G01 | 9.19 | 3CGF | 5.49 | 2ZAZ | 4.96 | 1GIH | 4.52 | 3DEJ |
| EF-057 | 4.57 | 2OJI | 4.87 | 2NO3 | 6.09 | 3CGF | 5.81 | 1W7H | 6.47 | 1PXI | 4.74 | 1GFW |
| EF-058 | 3.83 | 2OJI | 3.94 | 2NO3 | 4.92 | 3CGF | 4.99 | 1W7H | 6.02 | 1PXI | 3.96 | 1GFW |
| EF-059 | 3.42 | 1PME | 5.49 | 2G01 | 6.15 | 3CGF | 5.61 | 2ZAZ | 4.38 | 1GIH | 3.68 | 1GFW |
| EF-060 | 4.97 | 2OJI | 4.60 | 2NO3 | 4.53 | 1PMV | 5.38 | 1W7H | 4.33 | 1PXI | 4.05 | 3DEJ |
| EF-061 | 5.04 | 2OJI | 6.65 | 2G01 | 4.80 | 3CGF | 4.96 | 1DI9 | 3.86 | 2B53 | 4.26 | 1GFW |
| EF-062 | 5.74 | 2OJI | 4.59 | 2G01 | 6.65 | 3CGF | 6.70 | 2ZAZ | 4.24 | 2B53 | 5.74 | 3DEJ |
| EF-063 | 4.95 | 2OJI | 4.59 | 2G01 | 5.01 | 3CGF | 5.01 | 1DI9 | 5.87 | 1GIH | 4.28 | 1GFW |
| EF-064 | 4.31 | 2OJI | 4.93 | 2NO3 | 4.95 | 3CGF | 5.29 | 1W84 | 5.79 | 1GIH | 4.40 | 1GFW |
| EF-065 | 3.24 | 1PME | 6.07 | 2G01 | 4.87 | 3CGF | 4.78 | 1ZYJ | 3.45 | 1PXI | 4.12 | 1GFW |
| EF-066 | 4.26 | 2OJI | 4.35 | 2G01 | 5.44 | 3CGF | 4.08 | 1ZYJ | 4.89 | 1JVP | 4.00 | 1GFW |
| EF-067 | 3.95 | 2OJI | 3.74 | 2NO3 | 5.62 | 3CGF | 4.38 | 1DI9 | 4.48 | 1JVP | 3.97 | 3DEJ |
| EF-068 | 3.72 | 2OJI | 4.08 | 2NO3 | 5.12 | 3CGF | 5.46 | 1W7H | 4.69 | 2UZN | 3.61 | 1GFW |
| EF-069 | 3.35 | 2OJI | 3.85 | 2G01 | 4.94 | 3CGF | 4.16 | 1DI9 | 4.12 | 1JVP | 3.66 | 3DEJ |
| EF-070 | 4.00 | 2OJI | 5.01 | 2NO3 | 6.06 | 3CGF | 3.98 | 2ZAZ | 4.17 | 1JVP | 4.15 | 3DEJ |
| EF-071 | 5.21 | 2OJI | 4.11 | 1UKI | 4.96 | 3CGF | 4.46 | 1W84 | 6.02 | 1PXI | 4.38 | 3DEJ |
| EF-072 | 3.57 | 1PME | 3.85 | 2NO3 | 4.05 | 3CGF | 4.75 | 1DI9 | 4.30 | 1JVP | 3.90 | 3DEJ |
| EF-073 | 3.38 | 2OJI | 4.34 | 2G01 | 2.54 | 3CGF | 3.85 | 2ZAZ | 2.36 | 2UZN | 4.21 | 1GFW |
| EF-074 | 1.58 | 2OJI | 1.76 | 2G01 | 1.57 | 3CGF | 2.40 | 1ZYJ | 0.74 | 1JVP | 2.24 | 3DEJ |
| EF-075 | 3.67 | 1PME | 3.54 | 2G01 | 3.74 | 3CGF | 4.39 | 1W7H | 3.84 | 1GIH | 4.64 | 3DEJ |
| EF-076 | 5.87 | 2OJI | 4.57 | 1UKI | 5.81 | 3CGF | 4.97 | 1W7H | 4.54 | 1PXI | 3.65 | 3DEJ |
| EF-077 | 3.82 | 2OJI | 4.71 | 2G01 | 3.66 | 3CGF | 4.65 | 1W7H | 5.12 | 2B53 | 3.94 | 3DEJ |
| EF-078 | 0.69 | 2OJI | 0.82 | 2G01 | - | 3CGF | 1.41 | 1ZYJ | 1.80 | 2R3F | 2.01 | 3DEJ |
| EF-079 | 3.23 | 1PME | 3.86 | 2NO3 | 2.13 | 3CGF | 3.71 | 1ZYJ | 4.25 | 1PXI | 3.23 | 3DEJ |
| EF-080 | 3.64 | 2OJI | 3.79 | 2NO3 | 3.25 | 3CGF | 5.64 | 2ZAZ | 2.86 | 1PXI | 3.14 | 3DEJ |
| EF-081 | 4.03 | 2OJI | 5.03 | 2NO3 | 5.67 | 3CGF | 4.79 | 2ZAZ | 3.10 | 2R3F | 4.84 | 1GFW |
| EF-082 | 5.01 | 2OJI | 3.89 | 2G01 | 3.36 | 3CGF | 4.03 | 1DI9 | 5.15 | 2B53 | 3.63 | 3DEJ |
| EF-083 | 4.37 | 2OJI | 4.48 | 1UKI | 4.71 | 3CGF | 4.13 | 1W7H | 4.13 | 2UZN | 3.63 | 1GFW |
| EF-084 | 4.54 | 2OJI | 4.95 | 2NO3 | 4.69 | 3CGF | 4.50 | 2ZAZ | 4.08 | 2B53 | 3.43 | 1GFW |
| EF-085 | 4.23 | 2OJI | 5.95 | 2G01 | 4.66 | 3CGF | 4.98 | 1W7H | 5.59 | 2B53 | 4.35 | 1GFW |
| EF-086 | 3.79 | 2OJI | 4.72 | 2NO3 | 6.25 | 3CGF | 4.27 | 1W7H | 4.62 | 1PXI | 4.50 | 1GFW |
| EF-087 | 3.42 | 1PME | 5.17 | 2G01 | 5.94 | 3CGF | 4.75 | 2ZAZ | 4.16 | 1PXI | 4.24 | 3DEJ |
| EF-088 | 4.01 | 2OJI | 3.75 | 2G01 | 4.52 | 3CGF | 4.61 | 1DI9 | 3.36 | 1PXI | 4.72 | 3DEJ |
| EF-089 | 5.17 | 2OJI | 6.12 | 2G01 | 4.87 | 3CGF | 5.83 | 2ZAZ | 6.67 | 1GIH | 5.29 | 1GFW |
| EF-090 | 5.84 | 2OJI | 6.63 | 1UKI | 6.27 | 1PMV | 5.12 | 1DI9 | 6.24 | 2R3F | 6.19 | 3DEJ |
| EF-091 | 5.53 | 2OJI | 4.54 | 2G01 | 3.92 | 3CGF | 5.54 | 2ZAZ | 5.35 | 1PXI | 4.94 | 1GFW |
| EF-092 | 4.80 | 2OJI | 5.25 | 1UKI | 7.83 | 3CGF | 4.18 | 1DI9 | 7.79 | 1PXI | 5.37 | 3DEJ |
| EF-093 | 5.28 | 2OJI | 4.12 | 2G01 | 5.15 | 3CGF | 4.35 | 2ZAZ | 2.53 | 2B53 | 4.43 | 3DEJ |
| EF-094 | 3.83 | 2OJI | 3.51 | 2G01 | 3.77 | 3CGF | 3.46 | 1ZYJ | 3.27 | 2UZN | 3.76 | 1GFW |
| EF-095 | 5.02 | 2OJI | 5.27 | 2G01 | 5.79 | 3CGF | 4.87 | 1DI9 | 7.15 | 1GIH | 4.22 | 3DEJ |
| EF-096 | 4.13 | 2OJI | 3.69 | 2G01 | 5.08 | 3CGF | 4.87 | 1ZYJ | 4.32 | 1GIH | 3.98 | 1GFW |
| EF-097 | 3.33 | 2OJI | 5.02 | 2G01 | 5.52 | 3CGF | 3.94 | 1DI9 | 5.98 | 1PXI | 4.97 | 3DEJ |
| EF-098 | 4.34 | 2OJI | 4.64 | 2NO3 | 5.05 | 3CGF | 3.68 | 2ZAZ | 4.60 | 1GIH | 5.17 | 3DEJ |
| EF-099 | 7.02 | 2OJI | 4.82 | 2NO3 | 4.32 | 3CGF | 6.01 | 1W84 | 7.81 | 1PXI | 4.53 | 3DEJ |
| EF-100 | 4.49 | 2OJI | 3.89 | 1UKI | 5.04 | 3CGF | 4.88 | 2ZAZ | 4.91 | 1GIH | 5.14 | 1GFW |
| EF-101 | 5.50 | 2OJI | 4.76 | 2G01 | 6.44 | 3CGF | 4.95 | 2ZAZ | 6.15 | 1PXI | 3.61 | 3DEJ |
| EF-102 | 4.84 | 2OJI | 5.04 | 2G01 | 3.66 | 3CGF | 6.58 | 2ZAZ | 5.97 | 1JVP | 4.79 | 3DEJ |
| EF-103 | 3.79 | 1PME | 4.35 | 2G01 | 4.19 | 3CGF | 5.04 | 2ZAZ | 6.21 | 2B53 | 3.42 | 1GFW |
| EF-104 | 3.46 | 2OJI | 5.00 | 2G01 | 6.34 | 3CGF | 6.11 | 1W84 | 5.55 | 2UZN | 4.26 | 1GFW |
| EF-105 | 3.60 | 2OJI | 3.58 | 1UKI | 5.64 | 3CGF | 5.40 | 1W7H | 5.43 | 1PXI | 4.49 | 3DEJ |
| EF-106 | 4.59 | 2OJI | 4.77 | 2NO3 | 6.86 | 3CGF | 3.74 | 1W84 | 6.00 | 1JVP | 3.94 | 3DEJ |
| EF-107 | 4.24 | 2OJI | 4.48 | 2G01 | 4.74 | 3CGF | 4.83 | 1W7H | 4.76 | 1GIH | 4.30 | 1GFW |
| EF-108 | 3.58 | 2OJI | 6.10 | 2G01 | 5.38 | 3CGF | 5.18 | 2ZAZ | 4.84 | 2B53 | 5.68 | 3DEJ |
| EF-109 | 3.85 | 1PME | 5.63 | 2NO3 | 6.05 | 3CGF | 3.81 | 1ZYJ | 6.38 | 1JVP | 4.98 | 1GFW |
| EF-110 | 4.94 | 2OJI | 3.78 | 2NO3 | 5.27 | 3CGF | 4.94 | 1DI9 | 5.68 | 2B53 | 5.29 | 1GFW |
| EF-111 | 4.18 | 2OJI | 4.43 | 2G01 | 4.64 | 3CGF | 4.52 | 2ZAZ | 5.34 | 1JVP | 3.82 | 3DEJ |
| EF-112 | 4.15 | 2OJI | 4.60 | 2NO3 | 5.92 | 3CGF | 4.21 | 2ZAZ | 7.63 | 1PXI | 4.50 | 1GFW |
| EF-113 | 3.91 | 2OJI | 4.69 | 1UKI | 4.85 | 3CGF | 3.67 | 2ZAZ | 3.26 | 2UZN | 3.29 | 3DEJ |
| EF-114 | 3.69 | 2OJI | 5.36 | 2G01 | 4.43 | 3CGF | 4.52 | 2ZAZ | 5.67 | 2UZN | 4.08 | 3DEJ |
| EF-115 | 4.06 | 2OJI | 4.49 | 2NO3 | 4.32 | 3CGF | 4.62 | 2ZAZ | 4.96 | 2B53 | 3.78 | 3DEJ |
| EF-116 | 4.96 | 2OJI | 4.40 | 1UKI | 5.16 | 3CGF | 4.87 | 2ZAZ | 3.11 | 2UZN | 3.11 | 1GFW |
| EF-117 | 3.76 | 2OJI | 2.98 | 2G01 | 4.86 | 3CGF | 4.04 | 2ZAZ | 4.38 | 2UZN | 3.31 | 1GFW |
| EF-118 | 3.80 | 2OJI | 4.83 | 2NO3 | 4.35 | 3CGF | 3.44 | 1W84 | 4.86 | 2B53 | 3.40 | 3DEJ |
| EF-119 | 4.59 | 2OJI | 5.71 | 2NO3 | 4.48 | 3CGF | 4.72 | 1W7H | 4.22 | 2R3F | 3.73 | 3DEJ |
| EF-120 | 2.49 | 2OJI | 4.78 | 2G01 | 6.59 | 3CGF | 5.41 | 1W7H | 4.76 | 2B53 | 2.86 | 1GFW |
| EF-121 | 5.35 | 2OJI | 5.18 | 2G01 | 4.08 | 3CGF | 3.82 | 1DI9 | 4.23 | 2B53 | 3.52 | 3DEJ |
| EF-122 | 3.98 | 2OJI | 3.42 | 2NO3 | 5.08 | 3CGF | 4.17 | 1DI9 | 3.73 | 1GIH | 4.75 | 1GFW |
| EF-123 | 5.20 | 2OJI | 5.09 | 2G01 | 4.84 | 3CGF | 4.54 | 1W7H | 4.42 | 1PXI | 4.51 | 3DEJ |
| EF-124 | 5.38 | 2OJI | 5.39 | 2G01 | 6.31 | 3CGF | 3.98 | 1W7H | 5.99 | 1GIH | 4.18 | 1GFW |
| EF-125 | 3.36 | 2OJI | 5.90 | 2G01 | 4.64 | 3CGF | 4.87 | 1DI9 | 3.84 | 2R3F | 3.86 | 3DEJ |
| EF-126 | 3.44 | 2OJI | 2.63 | 2NO3 | 5.15 | 3CGF | 4.78 | 1W7H | 3.31 | 2B53 | 3.98 | 3DEJ |
| EF-127 | 3.77 | 2OJI | 5.14 | 2NO3 | 3.51 | 3CGF | 5.23 | 1W7H | 3.48 | 1PXI | 3.63 | 3DEJ |
| EF-128 | 3.53 | 2OJI | 3.70 | 2G01 | 4.63 | 3CGF | 4.07 | 1W84 | 4.22 | 2R3F | 4.14 | 3DEJ |
| EF-129 | 3.26 | 2OJI | 3.36 | 2G01 | 5.95 | 3CGF | 4.81 | 2ZAZ | 2.51 | 2B53 | 4.70 | 3DEJ |
| EF-130 | 3.96 | 2OJI | 4.63 | 2NO3 | 4.38 | 3CGF | 4.81 | 2ZAZ | 4.50 | 1PXI | 4.37 | 3DEJ |
| EF-131 | 3.38 | 2OJI | 3.54 | 2NO3 | 4.54 | 3CGF | 4.39 | 1DI9 | 4.43 | 2B53 | 4.05 | 3DEJ |
| EF-132 | 3.75 | 2OJI | 4.09 | 2G01 | 2.46 | 3CGF | 4.61 | 1DI9 | 4.08 | 1GIH | 3.63 | 1GFW |
| EF-133 | 4.37 | 2OJI | 3.35 | 2G01 | 3.85 | 3CGF | 4.53 | 2ZAZ | 4.61 | 2R3F | 4.04 | 3DEJ |
| EF-134 | 3.16 | 1PME | 4.14 | 2G01 | 4.89 | 3CGF | 4.23 | 2ZAZ | 4.39 | 2R3F | 4.71 | 3DEJ |
| EF-135 | 3.46 | 2OJI | 4.08 | 1UKI | 3.52 | 3CGF | 4.49 | 1DI9 | 3.74 | 2R3F | 4.59 | 3DEJ |
| EF-136 | 4.93 | 2OJI | 4.63 | 2G01 | 4.44 | 3CGF | 3.49 | 1DI9 | 4.02 | 2R3F | 5.02 | 1GFW |
| EF-137 | 3.81 | 2OJI | 3.46 | 2G01 | 4.21 | 3CGF | 2.96 | 2ZAZ | 3.04 | 2B53 | 4.76 | 3DEJ |
| EF-138 | 4.01 | 2OJI | 3.84 | 1UKI | 2.87 | 3CGF | 4.17 | 2ZAZ | 4.91 | 2B53 | 4.71 | 3DEJ |
| EF-139 | 4.02 | 2OJI | 4.52 | 1UKI | 4.40 | 3CGF | 4.51 | 2ZAZ | 4.42 | 2UZN | 3.88 | 1GFW |
| EF-140 | 4.58 | 2OJI | 3.53 | 2NO3 | 4.69 | 3CGF | 5.23 | 2ZAZ | 4.08 | 2B53 | 4.40 | 3DEJ |
| EF-141 | 3.53 | 2OJI | 4.68 | 1UKI | 5.96 | 3CGF | 4.70 | 1W7H | 4.58 | 1PXI | 4.63 | 3DEJ |
| EF-142 | 5.19 | 2OJI | 5.13 | 2G01 | 4.96 | 3CGF | 4.63 | 1DI9 | 5.19 | 2B53 | 4.23 | 1GFW |
| EF-143 | 4.77 | 2OJI | 5.46 | 2NO3 | 4.95 | 3CGF | 5.18 | 1W7H | 7.00 | 2B53 | 3.53 | 3DEJ |
| EF-144 | 3.62 | 2OJI | 5.67 | 2G01 | 4.57 | 3CGF | 4.26 | 2ZAZ | 4.35 | 2UZN | 4.31 | 3DEJ |
| EF-145 | 3.06 | 2OJI | 5.41 | 2G01 | 5.46 | 3CGF | 4.44 | 1W84 | 3.68 | 2B53 | 3.26 | 1GFW |
| EF-146 | 3.38 | 2OJI | 3.35 | 1UKI | 6.25 | 3CGF | 4.29 | 2ZAZ | 3.64 | 2UZN | 4.42 | 3DEJ |
| EF-147 | 4.21 | 2OJI | 4.27 | 2NO3 | 6.18 | 3CGF | 4.29 | 1W7H | 4.16 | 2B53 | 4.15 | 3DEJ |
| EF-148 | 4.81 | 2OJI | 3.55 | 2G01 | 4.37 | 3CGF | 3.76 | 2ZAZ | 4.65 | 1PXI | 5.71 | 3DEJ |
| EF-149 | 3.84 | 2OJI | 2.66 | 2NO3 | 2.92 | 3CGF | 4.37 | 2ZAZ | 2.17 | 2UZN | 3.75 | 1GFW |
| EF-150 | 4.26 | 2OJI | 4.31 | 2NO3 | 4.35 | 3CGF | 3.71 | 2ZAZ | 2.86 | 2UZN | 4.50 | 3DEJ |
| EF-151 | 4.01 | 2OJI | 3.97 | 2G01 | 2.93 | 3CGF | 3.84 | 1DI9 | 4.17 | 2B53 | 3.95 | 3DEJ |
| EF-152 | 4.55 | 2OJI | 4.40 | 2G01 | 4.11 | 3CGF | 4.64 | 1W7H | 3.56 | 1PXI | 3.21 | 1GFW |
| EF-153 | 3.94 | 2OJI | 3.42 | 2NO3 | 3.96 | 3CGF | 4.00 | 1W7H | 4.53 | 2B53 | 3.72 | 3DEJ |
| EF-154 | 2.74 | 2OJI | 4.72 | 2NO3 | 4.05 | 3CGF | 5.18 | 1W7H | 3.66 | 2B53 | 3.03 | 1GFW |
| EF-155 | 4.85 | 2OJI | 3.82 | 2NO3 | 3.90 | 3CGF | 3.51 | 1W84 | 3.31 | 1GIH | 3.29 | 3DEJ |
| EF-156 | 3.03 | 2OJI | 5.13 | 2NO3 | 4.33 | 3CGF | 4.66 | 2ZAZ | 4.00 | 1PXI | 3.74 | 3DEJ |
| EF-157 | 2.42 | 2OJI | 2.79 | 2NO3 | 2.20 | 3CGF | 2.54 | 1W84 | 2.32 | 2UZN | 2.39 | 3DEJ |
| EF-158 | 3.48 | 2OJI | 2.53 | 2G01 | 4.89 | 3CGF | 4.48 | 2ZAZ | 3.89 | 2B53 | 3.45 | 3DEJ |
| EF-159 | 3.47 | 2OJI | 3.18 | 1UKI | 4.82 | 3CGF | 4.00 | 2ZAZ | 4.05 | 1PXI | 3.19 | 3DEJ |
| EF-160 | 4.25 | 2OJI | 6.60 | 2NO3 | 5.69 | 3CGF | 4.77 | 1DI9 | 4.67 | 1JVP | 3.34 | 3DEJ |
| EF-161 | 5.52 | 2OJI | 4.22 | 2G01 | 4.57 | 3CGF | 3.20 | 1DI9 | 4.38 | 1JVP | 4.15 | 3DEJ |
| EF-162 | 4.10 | 2OJI | 4.34 | 2NO3 | 4.22 | 3CGF | 4.82 | 1W7H | 3.90 | 1PXI | 4.47 | 3DEJ |
| EF-163 | 2.96 | 2OJI | 3.50 | 2G01 | 4.56 | 3CGF | 5.37 | 1W7H | 3.39 | 1PXI | 3.09 | 3DEJ |
| EF-164 | 3.14 | 1PME | 3.83 | 2G01 | 3.31 | 3CGF | 4.23 | 2ZAZ | 3.73 | 1GIH | 5.30 | 3DEJ |
| EF-165 | 4.36 | 2OJI | 4.95 | 2G01 | 1.80 | 3CGF | 3.60 | 1DI9 | 3.73 | 1PXI | 3.59 | 1GFW |
| EF-166 | 3.24 | 2OJI | 4.07 | 2G01 | 5.56 | 3CGF | 3.25 | 1DI9 | 2.32 | 2B53 | 5.33 | 3DEJ |
| EF-167 | 3.82 | 1PME | 3.24 | 2G01 | 4.77 | 3CGF | 4.02 | 1DI9 | 3.90 | 2UZN | 5.66 | 3DEJ |
| EF-168 | 2.85 | 2OJI | 3.90 | 2G01 | 4.59 | 3CGF | 4.12 | 2ZAZ | 2.98 | 1GIH | 3.18 | 3DEJ |
| EF-169 | 4.46 | 2OJI | 4.09 | 2G01 | 4.84 | 3CGF | 4.36 | 1W7H | 5.18 | 1PXI | 3.46 | 1GFW |
| EF-170 | 5.64 | 2OJI | 5.77 | 2NO3 | 5.81 | 3CGF | 4.94 | 1W84 | 6.70 | 1JVP | 4.67 | 3DEJ |
| EF-171 | 4.78 | 1PME | 5.41 | 2NO3 | 5.34 | 3CGF | 5.01 | 1DI9 | 6.25 | 1JVP | 4.36 | 1GFW |
| EF-172 | 5.15 | 2OJI | 5.70 | 2G01 | 4.90 | 3CGF | 5.46 | 1W7H | 5.50 | 1PXI | 4.48 | 1GFW |
| EF-173 | 5.07 | 1PME | 6.54 | 2G01 | 3.51 | 3CGF | 5.64 | 1DI9 | 5.55 | 2B53 | 4.99 | 1GFW |
| EF-174 | 3.26 | 2OJI | 3.52 | 2NO3 | - | 3CGF | 3.73 | 1W7H | 1.31 | 1GIH | 4.45 | 1GFW |
| EF-175 | 4.67 | 1PME | 2.99 | 1UKI | 2.48 | 3CGF | 3.31 | 1W84 | 3.34 | 2B53 | 3.98 | 3DEJ |
| EF-176 | 3.89 | 2OJI | 3.75 | 2G01 | 2.52 | 3CGF | 3.91 | 2ZAZ | 3.33 | 2B53 | 4.05 | 3DEJ |
| EF-177 | 4.24 | 2OJI | 5.19 | 2G01 | 4.82 | 3CGF | 4.93 | 1W84 | 2.95 | 1GIH | 3.29 | 3DEJ |

| No. | EGFR | | ESR1 | | PGR | | GSTP1 | | SRC | | MMP3 | | MMP13 | |
| --- | --- | --- | --- | --- | --- | --- | --- | --- | --- | --- | --- | --- | --- | --- |
|  | Score | PDB | Score | PDB | Score | PDB | Score | PDB | Score | PDB | Score | PDB | Score | PDB |
| EF-001 | 6.62 | 1M17 | 5.57 | 1ERE | 3.35 | 1SQN | 3.87 | 19GS | 6.85 | 2H8H | 3.09 | 1BM6 | 4.57 | 830C |
| EF-002 | 6.14 | 2J5F | 0.70 | 1QKT | 6.17 | 2OVM | 7.26 | 19GS | 7.25 | 1YOM | 5.26 | 1BM6 | 7.08 | 830C |
| EF-003 | 4.53 | 1M17 | - | 1QKT | 7.59 | 2OVH | 6.31 | 1MD3 | 7.71 | 2BDJ | 3.03 | 1BM6 | 4.73 | 830C |
| EF-004 | 4.38 | 1M17 | 5.16 | 1QKT | 4.00 | 2OVM | 4.11 | 1LBK | 4.28 | 2H8H | 3.24 | 1BM6 | 4.33 | 830C |
| EF-005 | 6.63 | 1M17 | - | 1QKT | 7.02 | 2OVM | 6.27 | 19GS | 5.92 | 2H8H | 5.15 | 1BM6 | 5.29 | 830C |
| EF-006 | 6.02 | 1M17 | 4.04 | 1QKU | 3.07 | 2OVM | 4.21 | 19GS | 4.06 | 2BDJ | 3.66 | 1BM6 | 4.32 | 1EUB |
| EF-007 | 6.38 | 1M17 | 5.41 | 1QKU | 3.25 | 2OVH | 4.36 | 19GS | 4.92 | 2H8H | 2.62 | 1BM6 | 4.71 | 1EUB |
| EF-008 | 1.51 | 2J5F | 4.10 | 2Q70 | 4.94 | 1A28 | 4.18 | 1LBK | 3.91 | 2H8H | 3.79 | 1BM6 | 3.76 | 830C |
| EF-009 | 2.64 | 1M17 | 4.01 | 2Q70 | 5.00 | 2OVH | 3.35 | 19GS | 3.90 | 2H8H | 3.60 | 1BM6 | 3.32 | 830C |
| EF-010 | 2.61 | 2J5F | 3.58 | 1QKT | 3.69 | 1SQN | 3.33 | 1LBK | 4.07 | 1YOM | 3.06 | 1BM6 | 4.46 | 1EUB |
| EF-011 | 5.95 | 1M17 | - | 2Q70 | 3.14 | 2OVH | 9.13 | 19GS | 7.48 | 2H8H | 6.00 | 1BM6 | 10.70 | 830C |
| EF-012 | 3.57 | 2ITY | 5.19 | 1QKT | 4.94 | 2OVM | 3.93 | 1LBK | 3.94 | 2H8H | 3.19 | 1BM6 | 4.32 | 830C |
| EF-013 | 5.21 | 2ITY | - | 2QE4 | 4.32 | 2OVM | 9.91 | 19GS | 6.46 | 1YOM | 5.24 | 1BM6 | 8.82 | 830C |
| EF-014 | 4.66 | 2ITY | 0.93 | 1QKT | 3.44 | 2OVH | 10.05 | 19GS | 6.54 | 2H8H | 3.78 | 1BM6 | 10.47 | 830C |
| EF-015 | 7.79 | 1M17 | 1.92 | 1QKT | 5.82 | 2OVM | 8.30 | 1MD3 | 7.07 | 2H8H | 5.40 | 1BM6 | 7.67 | 830C |
| EF-016 | 5.49 | 2ITY | - | 1QKT | 4.38 | 2OVH | 8.42 | 1LBK | 8.54 | 1YOM | 5.99 | 1BM6 | 8.94 | 830C |
| EF-017 | 3.32 | 2ITY | 0.86 | 1G50 | 5.34 | 2OVM | 4.22 | 1LBK | 4.24 | 1YOM | 3.11 | 1BM6 | 3.48 | 830C |
| EF-018 | 6.59 | 1M17 | 4.87 | 1G50 | 4.85 | 2OVM | 3.32 | 19GS | 4.13 | 2H8H | 2.45 | 1BM6 | 3.98 | 1EUB |
| EF-019 | 7.24 | 1M17 | 4.91 | 2QE4 | 5.55 | 2OVM | 4.54 | 19GS | 4.97 | 1YOM | 2.04 | 1BM6 | 4.89 | 830C |
| EF-020 | 6.48 | 1M17 | 5.95 | 2QE4 | 4.39 | 2OVM | 5.21 | 19GS | 3.93 | 2H8H | 3.21 | 1BM6 | 3.77 | 830C |
| EF-021 | 7.63 | 1M17 | 0.56 | 2QE4 | 6.66 | 2OVH | 6.30 | 1LBK | 8.18 | 2H8H | 4.43 | 1BM6 | 8.56 | 830C |
| EF-022 | 4.77 | 2ITY | 4.83 | 2QE4 | 4.03 | 2OVH | 3.49 | 19GS | 3.72 | 2BDJ | 2.83 | 1BM6 | 4.75 | 830C |
| EF-023 | 6.00 | 1M17 | 3.26 | 2QE4 | 3.93 | 2OVH | 4.20 | 19GS | 4.35 | 2BDJ | 2.76 | 1BM6 | 3.74 | 830C |
| EF-024 | 6.49 | 1M17 | 0.35 | 2QE4 | 3.57 | 2OVH | 7.80 | 19GS | 6.53 | 1YOM | 6.17 | 1BM6 | 7.36 | 830C |
| EF-025 | 6.79 | 1M17 | 3.98 | 2QE4 | 2.99 | 2OVH | 3.41 | 1LBK | 5.89 | 1YOM | 3.23 | 1BM6 | 5.20 | 830C |
| EF-026 | 8.54 | 2ITY | - | 2QE4 | 6.79 | 2OVH | 11.57 | 19GS | 8.45 | 2H8H | 3.52 | 1BM6 | 5.91 | 830C |
| EF-027 | 5.03 | 1M17 | 4.02 | 2QE4 | 4.33 | 2OVH | 5.91 | 19GS | 3.84 | 2H8H | 3.47 | 1BM6 | 5.43 | 830C |
| EF-028 | 6.63 | 1M17 | 4.37 | 2QE4 | 2.95 | 2OVM | 4.61 | 19GS | 3.23 | 2H8H | 2.92 | 1BM6 | 4.80 | 830C |
| EF-029 | 6.10 | 1M17 | 6.49 | 1QKT | 4.35 | 2OVM | 8.10 | 19GS | 6.87 | 1YOM | 5.41 | 1BM6 | 8.11 | 830C |
| EF-030 | 6.47 | 1M17 | 7.69 | 1QKT | 3.20 | 2OVM | 7.89 | 19GS | 9.07 | 2BDJ | 3.90 | 1BM6 | 5.88 | 830C |
| EF-031 | 7.86 | 2ITY | 6.92 | 1QKT | 4.45 | 2OVM | 9.83 | 19GS | 4.63 | 1YOM | 3.78 | 1BM6 | 7.64 | 830C |
| EF-032 | 6.79 | 1M17 | 5.96 | 1QKT | 4.50 | 2OVM | 8.62 | 19GS | 7.61 | 2H8H | 3.84 | 1BM6 | 8.45 | 830C |
| EF-033 | 6.92 | 1M17 | 0.95 | 2QE4 | 4.75 | 2OVH | 8.30 | 1LBK | 8.13 | 2H8H | 5.74 | 1BM6 | 7.53 | 830C |
| EF-034 | 4.49 | 1M17 | 4.56 | 1QKT | 4.54 | 2OVH | 3.51 | 1LBK | 3.78 | 2H8H | 4.38 | 1BM6 | 4.68 | 830C |
| EF-035 | 5.23 | 2ITY | 2.11 | 2QE4 | 7.10 | 2OVM | 5.29 | 1LBK | 4.46 | 2H8H | 4.55 | 1BM6 | 3.95 | 1EUB |
| EF-036 | 6.92 | 2ITY | 4.23 | 2QE4 | 6.27 | 2OVH | 4.06 | 19GS | 3.47 | 1O4J | 3.87 | 1BM6 | 3.41 | 1EUB |
| EF-037 | 7.52 | 2ITY | - | 2Q70 | 5.44 | 2OVM | 7.30 | 1LBK | 8.60 | 2H8H | 5.50 | 1BM6 | 6.76 | 830C |
| EF-038 | 6.71 | 1M17 | 0.27 | 2Q70 | 6.72 | 2OVH | 6.78 | 1LBK | 8.75 | 2H8H | 5.09 | 1BM6 | 6.12 | 830C |
| EF-039 | 7.43 | 1M17 | - | 1QKT | 7.77 | 2OVM | 5.78 | 19GS | 7.40 | 2H8H | 4.92 | 1BM6 | 4.79 | 830C |
| EF-040 | 5.99 | 2ITY | - | 2Q70 | 5.57 | 2OVH | 8.11 | 19GS | 6.44 | 2H8H | 2.74 | 1BM6 | 3.79 | 1EUB |
| EF-041 | 7.44 | 2ITY | - | 2QE4 | 4.60 | 2OVM | 7.22 | 19GS | 7.87 | 2BDJ | 4.01 | 1BM6 | 5.57 | 830C |
| EF-042 | 4.79 | 2ITY | 3.99 | 1QKT | 3.71 | 2OVH | 10.10 | 1LBK | 6.96 | 2H8H | 3.95 | 1BM6 | 6.09 | 830C |
| EF-043 | 6.05 | 2J5F | 2.06 | 2QE4 | 7.42 | 2OVH | 6.17 | 1LBK | 8.18 | 2H8H | 3.44 | 1BM6 | 4.33 | 1EUB |
| EF-044 | 5.88 | 2ITY | - | 2QE4 | 5.06 | 2OVH | 7.21 | 19GS | 7.93 | 2H8H | 4.41 | 1BM6 | 5.40 | 830C |
| EF-045 | 7.28 | 2ITY | 1.23 | 1QKT | 4.47 | 2OVM | 7.52 | 1LBK | 5.82 | 1YOM | 4.82 | 1BM6 | 6.78 | 1EUB |
| EF-046 | 7.96 | 1M17 | 1.77 | 2Q70 | 4.98 | 2OVH | 7.28 | 1LBK | 9.35 | 2H8H | 5.17 | 1BM6 | 8.40 | 830C |
| EF-047 | 2.88 | 2ITY | - | 1QKT | - | 2OVM | 4.34 | 1LBK | 3.15 | 1O4J | 2.69 | 1BM6 | 2.64 | 830C |
| EF-048 | 2.72 | 2ITY | - | 1QKT | - | 2OVH | 6.42 | 1LBK | 3.17 | 1O4J | 3.16 | 1BM6 | 2.62 | 830C |
| EF-049 | 2.38 | 2ITY | - | 1QKT |  | 2OVM | 4.90 | 19GS | 4.32 | 2BDJ | 3.37 | 1BM6 | 2.13 | 830C |
| EF-050 | 2.95 | 1M17 | - | 1QKT | - | 2OVM | 4.26 | 19GS | 3.61 | 2BDJ | 2.74 | 1BM6 | 2.61 | 830C |
| EF-051 | 4.09 | 2ITY | 5.03 | 2B1Z | 4.62 | 2OVH | 4.45 | 1LBK | 5.71 | 2BDJ | 3.86 | 1BM6 | 4.92 | 830C |
| EF-052 | 4.81 | 1M17 | 6.01 | 1QKT | 5.14 | 1A28 | 4.87 | 1LBK | 6.43 | 2BDJ | 4.13 | 1BM6 | 4.72 | 830C |
| EF-053 | 5.11 | 1M17 | 4.26 | 1QKU | 3.93 | 2OVM | 4.61 | 1LBK | 5.01 | 2BDJ | 3.16 | 1BM6 | 4.31 | 830C |
| EF-054 | 5.72 | 2ITY | 3.04 | 1QKT | 3.57 | 2OVM | 4.09 | 19GS | 4.62 | 2H8H | 4.87 | 1BM6 | 3.09 | 830C |
| EF-055 | 3.70 | 1M17 | 6.88 | 1QKU | 5.47 | 2OVM | 3.99 | 1MD3 | 4.01 | 1O4J | 4.25 | 1BM6 | 3.35 | 830C |
| EF-056 | 4.72 | 2ITY | 5.35 | 1QKT | 6.29 | 2OVH | 5.85 | 19GS | 6.12 | 2BDJ | 3.87 | 1BM6 | 3.25 | 830C |
| EF-057 | 4.21 | 1M17 | 5.45 | 2Q70 | 5.17 | 1SQN | 3.86 | 19GS | 4.53 | 1YOM | 3.37 | 1BM6 | 3.65 | 830C |
| EF-058 | 3.66 | 2J5F | 4.82 | 1QKT | 4.74 | 1A28 | 3.83 | 19GS | 3.10 | 2H8H | 3.44 | 1BM6 | 3.94 | 830C |
| EF-059 | 3.00 | 1M17 | 6.94 | 1QKT | 5.21 | 1A28 | 3.44 | 1LBK | 3.68 | 1YOM | 3.27 | 1BM6 | 4.84 | 1EUB |
| EF-060 | 3.27 | 2J5F | 5.00 | 1QKT | 3.99 | 2OVH | 3.41 | 19GS | 3.05 | 2H8H | 3.81 | 1BM6 | 3.40 | 830C |
| EF-061 | 4.22 | 1M17 | 4.73 | 1QKT | 4.70 | 2OVH | 3.85 | 19GS | 4.00 | 2H8H | 3.88 | 1BM6 | 3.14 | 830C |
| EF-062 | 4.47 | 2ITY | 3.56 | 1QKT | 4.54 | 2OVM | 4.97 | 1LBK | 3.97 | 1O4J | 4.02 | 1BM6 | 3.85 | 830C |
| EF-063 | 5.55 | 1M17 | 6.10 | 2Q70 | 4.39 | 2OVH | 7.10 | 1LBK | 5.74 | 2BDJ | 4.58 | 1BM6 | 4.51 | 830C |
| EF-064 | 4.84 | 1M17 | 6.08 | 2Q70 | 4.15 | 2OVM | 3.33 | 1MD3 | 5.37 | 2BDJ | 3.97 | 1BM6 | 3.42 | 830C |
| EF-065 | 3.70 | 2J5F | 5.55 | 2Q70 | 5.98 | 1SQN | 4.48 | 1LBK | 3.70 | 1O4J | 3.49 | 1BM6 | 2.95 | 830C |
| EF-066 | 4.15 | 2ITY | 4.35 | 2B1Z | 3.97 | 1A28 | 3.87 | 19GS | 4.68 | 2BDJ | 4.00 | 1BM6 | 3.75 | 1EUB |
| EF-067 | 2.96 | 2J5F | 4.88 | 1QKT | 4.22 | 1A28 | 3.13 | 19GS | 2.50 | 1O4J | 3.84 | 1BM6 | 3.71 | 1EUB |
| EF-068 | 3.45 | 1M17 | 4.48 | 1QKU | 4.80 | 1A28 | 4.82 | 1LBK | 4.39 | 2BDJ | 3.41 | 1BM6 | 4.52 | 1EUB |
| EF-069 | 3.87 | 2ITY | 4.23 | 2B1Z | 5.12 | 1A28 | 3.84 | 19GS | 4.51 | 2H8H | 4.18 | 1BM6 | 3.94 | 830C |
| EF-070 | 3.74 | 1M17 | 5.08 | 1QKT | 4.93 | 1A28 | 3.93 | 19GS | 4.58 | 2H8H | 3.30 | 1BM6 | 5.79 | 1EUB |
| EF-071 | 5.65 | 1M17 | 5.00 | 1QKT | 4.78 | 1A28 | 3.26 | 19GS | 3.17 | 2BDJ | 2.90 | 1BM6 | 2.86 | 1EUB |
| EF-072 | 3.79 | 1M17 | 3.41 | 2Q70 | 3.45 | 2OVH | 3.83 | 19GS | 3.98 | 1YOM | 3.33 | 1BM6 | 3.51 | 830C |
| EF-073 | 3.98 | 2ITY | 6.35 | 1G50 | 4.47 | 2OVM | 3.82 | 1MD3 | 4.67 | 2BDJ | 3.59 | 1BM6 | 2.54 | 1EUB |
| EF-074 | 1.01 | 1M17 | 2.72 | 1QKT | 1.93 | 2OVM | 1.84 | 19GS | 1.21 | 1YOM | 0.59 | 1BM6 | - | 830C |
| EF-075 | 3.38 | 1M17 | 5.26 | 1QKT | 5.75 | 1A28 | 4.12 | 19GS | 3.12 | 2BDJ | 4.00 | 1BM6 | 3.99 | 1EUB |
| EF-076 | 3.91 | 2J5F | 3.73 | 1QKU | 4.94 | 2OVM | 4.95 | 1LBK | 3.23 | 1O4J | 3.75 | 1BM6 | 4.13 | 830C |
| EF-077 | 4.00 | 2J5F | 5.79 | 1QKT | 6.17 | 1A28 | 2.85 | 19GS | 3.28 | 2BDJ | 4.04 | 1BM6 | 3.86 | 1EUB |
| EF-078 | 1.76 | 1M17 | - | 1QKU | 3.67 | 2OVH | 2.06 | 19GS | 2.30 | 1O4J | 1.07 | 1BM6 | 1.40 | 830C |
| EF-079 | 2.57 | 2ITY | 0.86 | 1QKT | 3.08 | 2OVM | 3.76 | 1LBK | 3.38 | 1O4J | 2.92 | 1BM6 | 4.28 | 830C |
| EF-080 | 7.02 | 1M17 | 3.39 | 1QKT | 5.09 | 1A28 | 3.34 | 19GS | 3.39 | 1O4J | 3.33 | 1BM6 | 4.22 | 830C |
| EF-081 | 6.34 | 2ITY | 0.19 | 2OCF | 3.15 | 2OVH | 4.70 | 1LBK | 3.97 | 1O4J | 2.79 | 1BM6 | 3.43 | 830C |
| EF-082 | 2.52 | 2ITY | 4.77 | 1QKT | 3.29 | 1A28 | 2.26 | 19GS | 4.23 | 2BDJ | 2.75 | 1BM6 | 4.90 | 1EUB |
| EF-083 | 3.43 | 2ITY | 5.08 | 1QKT | 3.26 | 1SQN | 3.77 | 1LBK | 3.96 | 2BDJ | 3.87 | 1BM6 | 3.96 | 830C |
| EF-084 | 4.50 | 1M17 | 4.62 | 1QKT | 4.62 | 1SQN | 3.86 | 19GS | 4.33 | 2BDJ | 5.06 | 1BM6 | 2.33 | 830C |
| EF-085 | 4.01 | 2ITY | 5.01 | 2Q70 | 3.46 | 1A28 | 3.91 | 1LBK | 3.63 | 1O4J | 3.29 | 1BM6 | 3.59 | 830C |
| EF-086 | 3.66 | 1M17 | 4.73 | 1QKT | 5.04 | 1SQN | 4.84 | 19GS | 3.88 | 1YOM | 3.38 | 1BM6 | 4.03 | 830C |
| EF-087 | 3.28 | 1M17 | 6.76 | 1QKT | 5.02 | 1SQN | 3.50 | 1LBK | 3.72 | 1YOM | 3.61 | 1BM6 | 4.81 | 1EUB |
| EF-088 | 4.59 | 2ITY | 2.08 | 1G50 | 5.10 | 2OVM | 3.68 | 1LBK | 2.95 | 2BDJ | 3.22 | 1BM6 | 2.87 | 830C |
| EF-089 | 6.55 | 1M17 | 1.64 | 1QKT | 4.29 | 2OVH | 6.08 | 1LBK | 6.36 | 2H8H | 3.55 | 1BM6 | 5.44 | 1EUB |
| EF-090 | 5.39 | 1M17 | 5.59 | 1QKT | 4.59 | 2OVH | 6.49 | 1LBK | 6.38 | 2H8H | 4.36 | 1BM6 | 3.70 | 830C |
| EF-091 | 4.82 | 1M17 | 3.53 | 1QKT | 5.53 | 2OVH | 6.30 | 1LBK | 6.85 | 2H8H | 3.52 | 1BM6 | 2.68 | 830C |
| EF-092 | 5.07 | 1M17 | 4.47 | 1QKT | 2.67 | 2OVM | 6.08 | 1LBK | 4.94 | 2BDJ | 4.31 | 1BM6 | 3.55 | 830C |
| EF-093 | 3.19 | 2ITY | 0.16 | 1G50 | 6.56 | 2OVH | 5.47 | 19GS | 3.36 | 1YOM | 3.51 | 1BM6 | 2.63 | 830C |
| EF-094 | 4.40 | 1M17 | 5.76 | 2B1Z | 4.01 | 1A28 | 4.88 | 19GS | 3.51 | 1YOM | 4.11 | 1BM6 | 3.01 | 830C |
| EF-095 | 4.33 | 1M17 | 4.32 | 2OCF | 5.61 | 2OVH | 4.63 | 1LBK | 3.70 | 2BDJ | 4.24 | 1BM6 | 4.11 | 830C |
| EF-096 | 3.95 | 1M17 | 5.15 | 1QKT | 5.51 | 1A28 | 4.48 | 1LBK | 3.99 | 1YOM | 3.91 | 1BM6 | 5.07 | 1EUB |
| EF-097 | 4.58 | 2ITY | 5.44 | 2Q70 | 5.86 | 2OVH | 3.84 | 1LBK | 5.38 | 2BDJ | 3.61 | 1BM6 | 3.72 | 1EUB |
| EF-098 | 3.98 | 1M17 | 5.43 | 1QKT | 5.54 | 1A28 | 3.84 | 1LBK | 4.59 | 1YOM | 3.49 | 1BM6 | 4.62 | 830C |
| EF-099 | 5.43 | 1M17 | 2.68 | 2QE4 | 2.42 | 2OVM | 5.27 | 1LBK | 6.48 | 2H8H | 4.17 | 1BM6 | 3.67 | 830C |
| EF-100 | 6.10 | 2ITY | 6.16 | 1QKT | 6.47 | 1SQN | 4.40 | 1LBK | 4.33 | 2H8H | 3.59 | 1BM6 | 4.72 | 830C |
| EF-101 | 5.15 | 1M17 | 5.48 | 1QKT | 2.54 | 2OVH | 5.40 | 19GS | 4.56 | 2H8H | 4.08 | 1BM6 | 3.99 | 830C |
| EF-102 | 4.23 | 1M17 | 6.00 | 1QKT | 5.86 | 2OVH | 3.78 | 1MD3 | 4.90 | 2BDJ | 3.75 | 1BM6 | 4.97 | 830C |
| EF-103 | 4.55 | 2J5F | 5.56 | 1QKU | 5.54 | 1A28 | 3.98 | 1LBK | 4.41 | 1YOM | 3.84 | 1BM6 | 4.33 | 1EUB |
| EF-104 | 3.38 | 1M17 | 6.86 | 1QKU | 4.77 | 1A28 | 3.61 | 1LBK | 3.43 | 2H8H | 3.41 | 1BM6 | 3.02 | 830C |
| EF-105 | 4.19 | 2J5F | 4.57 | 1QKU | 5.02 | 2OVM | 4.89 | 1LBK | 4.35 | 1YOM | 3.48 | 1BM6 | 3.28 | 830C |
| EF-106 | 3.43 | 2J5F | 5.00 | 1QKU | 4.81 | 1A28 | 4.37 | 1LBK | 5.42 | 2BDJ | 3.68 | 1BM6 | 5.00 | 830C |
| EF-107 | 5.34 | 2J5F | 4.54 | 1QKU | 4.36 | 2OVH | 5.13 | 1MD3 | 5.00 | 2BDJ | 3.32 | 1BM6 | 3.31 | 830C |
| EF-108 | 5.23 | 2ITY | 7.72 | 2B1Z | 5.30 | 1A28 | 4.61 | 1LBK | 4.33 | 1YOM | 4.24 | 1BM6 | 4.55 | 830C |
| EF-109 | 4.12 | 2ITY | 7.07 | 2B1Z | 5.46 | 1A28 | 3.84 | 1MD3 | 4.39 | 1YOM | 4.12 | 1BM6 | 4.34 | 830C |
| EF-110 | 5.19 | 1M17 | 6.12 | 2B1Z | 5.42 | 2OVH | 4.98 | 1MD3 | 5.20 | 2H8H | 4.11 | 1BM6 | 4.22 | 830C |
| EF-111 | 4.30 | 2ITY | 5.54 | 1QKT | 4.02 | 1A28 | 4.67 | 1LBK | 4.60 | 2BDJ | 3.94 | 1BM6 | 3.97 | 1EUB |
| EF-112 | 4.73 | 1M17 | 5.99 | 2OCF | 5.77 | 2OVH | 4.31 | 1LBK | 3.88 | 2BDJ | 3.87 | 1BM6 | 4.06 | 830C |
| EF-113 | 3.38 | 2ITY | 4.77 | 2Q70 | 5.32 | 2OVM | 3.68 | 19GS | 2.93 | 2H8H | 3.45 | 1BM6 | 3.12 | 830C |
| EF-114 | 2.98 | 2ITY | 4.32 | 2Q70 | 5.76 | 2OVM | 3.38 | 1LBK | 4.50 | 2H8H | 3.54 | 1BM6 | 3.76 | 830C |
| EF-115 | 3.97 | 1M17 | 3.18 | 2B1Z | 5.61 | 2OVM | 3.42 | 1MD3 | 4.09 | 2H8H | 2.47 | 1BM6 | 4.35 | 830C |
| EF-116 | 2.90 | 2ITY | 4.49 | 2Q70 | 5.98 | 2OVH | 3.92 | 1LBK | 3.58 | 2H8H | 3.86 | 1BM6 | 3.50 | 1EUB |
| EF-117 | 2.22 | 2J5F | 2.76 | 1G50 | 6.09 | 2OVH | 3.53 | 1MD3 | 2.66 | 1O4J | 3.68 | 1BM6 | 3.70 | 1EUB |
| EF-118 | 3.76 | 2ITY | 2.71 | 1QKT | 5.90 | 2OVM | 2.70 | 1LBK | 3.33 | 1YOM | 3.89 | 1BM6 | 2.91 | 1EUB |
| EF-119 | 4.13 | 2ITY | 2.56 | 2OCF | 6.36 | 2OVH | 2.73 | 19GS | 3.56 | 2H8H | 3.35 | 1BM6 | 3.40 | 1EUB |
| EF-120 | 3.38 | 1M17 | 3.83 | 1QKT | 5.83 | 1A28 | 3.48 | 1MD3 | 2.82 | 1YOM | 3.99 | 1BM6 | 4.48 | 1EUB |
| EF-121 | 4.39 | 2ITY | 0.74 | 1QKT | 5.65 | 1A28 | 4.36 | 1LBK | 3.28 | 2H8H | 3.91 | 1BM6 | 3.93 | 1EUB |
| EF-122 | 4.15 | 2ITY | 4.92 | 2Q70 | 7.48 | 1A28 | 4.07 | 1MD3 | 4.12 | 2H8H | 3.66 | 1BM6 | 5.49 | 1EUB |
| EF-123 | 4.91 | 2ITY | 4.94 | 2Q70 | 5.86 | 2OVH | 3.61 | 1MD3 | 3.19 | 1O4J | 2.96 | 1BM6 | 3.14 | 830C |
| EF-124 | 6.47 | 2J5F | 5.31 | 2Q70 | 5.78 | 1A28 | 5.74 | 1LBK | 4.09 | 2H8H | 3.58 | 1BM6 | 3.27 | 1EUB |
| EF-125 | 4.73 | 2ITY | 5.58 | 1QKT | 6.20 | 2OVH | 4.07 | 1LBK | 4.63 | 1O4J | 4.13 | 1BM6 | 4.37 | 830C |
| EF-126 | 2.82 | 2ITY | 4.02 | 1QKU | 4.60 | 2OVH | 3.48 | 19GS | 3.60 | 1O4J | 4.42 | 1BM6 | 2.34 | 1EUB |
| EF-127 | 3.40 | 1M17 | 5.46 | 1QKU | 5.80 | 2OVH | 3.77 | 1MD3 | 3.42 | 1YOM | 3.44 | 1BM6 | 2.99 | 1EUB |
| EF-128 | 2.09 | 1M17 | 3.32 | 2B1Z | 4.52 | 2OVM | 3.61 | 1LBK | 3.60 | 2BDJ | 2.64 | 1BM6 | 2.38 | 1EUB |
| EF-129 | 2.45 | 1M17 | 3.74 | 1G50 | 4.09 | 2OVH | 4.95 | 1LBK | 3.72 | 2BDJ | 2.64 | 1BM6 | 2.86 | 830C |
| EF-130 | 4.07 | 1M17 | 4.62 | 1G50 | 5.08 | 1A28 | 3.23 | 1LBK | 3.61 | 1O4J | 3.38 | 1BM6 | 3.18 | 830C |
| EF-131 | 3.46 | 1M17 | 3.06 | 1QKT | 4.97 | 1A28 | 4.36 | 1LBK | 2.53 | 1YOM | 3.17 | 1BM6 | 3.63 | 1EUB |
| EF-132 | 2.43 | 1M17 | 4.75 | 1QKT | 4.63 | 1A28 | 3.63 | 1LBK | 3.78 | 1YOM | 4.23 | 1BM6 | 2.11 | 830C |
| EF-133 | 4.13 | 1M17 | 4.85 | 1QKT | 5.31 | 2OVH | 2.66 | 19GS | 4.14 | 1O4J | 3.20 | 1BM6 | 3.70 | 830C |
| EF-134 | 3.90 | 2ITY | 6.17 | 1QKT | 5.32 | 2OVH | 3.54 | 19GS | 3.34 | 1O4J | 2.72 | 1BM6 | 2.72 | 1EUB |
| EF-135 | 1.98 | 1M17 | 4.59 | 1QKT | 4.73 | 2OVH | 2.95 | 1MD3 | 3.94 | 1YOM | 3.96 | 1BM6 | 3.15 | 1EUB |
| EF-136 | 4.13 | 2J5F | 5.72 | 1QKT | 5.10 | 2OVH | 3.01 | 1MD3 | 4.20 | 2BDJ | 3.21 | 1BM6 | 2.69 | 830C |
| EF-137 | 3.06 | 2ITY | 4.12 | 2Q70 | 4.98 | 1A28 | 3.42 | 1MD3 | 4.70 | 2BDJ | 3.00 | 1BM6 | 2.12 | 1EUB |
| EF-138 | 3.89 | 1M17 | 4.34 | 2Q70 | 3.85 | 2OVH | 4.10 | 1LBK | 4.21 | 2BDJ | 2.88 | 1BM6 | 2.14 | 830C |
| EF-139 | 3.60 | 1M17 | 3.78 | 1QKT | 4.86 | 2OVH | 3.61 | 1LBK | 3.56 | 2BDJ | 3.41 | 1BM6 | 2.78 | 1EUB |
| EF-140 | 3.66 | 2ITY | 3.46 | 2B1Z | 4.99 | 1A28 | 4.31 | 1LBK | 2.85 | 2BDJ | 2.62 | 1BM6 | 3.09 | 830C |
| EF-141 | 2.89 | 2ITY | 5.71 | 1G50 | 5.05 | 1A28 | 3.29 | 1MD3 | 4.50 | 2H8H | 3.44 | 1BM6 | 3.92 | 1EUB |
| EF-142 | 4.80 | 1M17 | 4.82 | 1QKT | 5.04 | 1A28 | 3.08 | 19GS | 3.36 | 2BDJ | 2.81 | 1BM6 | 2.77 | 830C |
| EF-143 | 4.08 | 1M17 | 4.86 | 1QKT | 4.32 | 1A28 | 4.16 | 19GS | 4.13 | 1O4J | 2.51 | 1BM6 | 3.62 | 830C |
| EF-144 | 4.51 | 2ITY | 4.16 | 1QKT | 4.74 | 2OVM | 3.51 | 1MD3 | 4.31 | 1O4J | 3.29 | 1BM6 | 4.29 | 1EUB |
| EF-145 | 1.58 | 2ITY | 4.31 | 1QKU | 3.78 | 1A28 | 3.84 | 1LBK | 2.38 | 1YOM | 2.99 | 1BM6 | 3.66 | 1EUB |
| EF-146 | 3.29 | 2ITY | 5.43 | 1QKT | 6.09 | 2OVH | 3.70 | 1MD3 | 3.07 | 1O4J | 3.80 | 1BM6 | 4.00 | 1EUB |
| EF-147 | 3.35 | 1M17 | 5.47 | 1QKT | 4.89 | 2OVM | 4.53 | 19GS | 3.49 | 2H8H | 4.03 | 1BM6 | 3.47 | 1EUB |
| EF-148 | 2.41 | 1M17 | 2.99 | 2Q70 | 5.58 | 1A28 | 5.07 | 19GS | 4.33 | 2BDJ | 3.45 | 1BM6 | 2.83 | 1EUB |
| EF-149 | 2.35 | 2ITY | 2.01 | 2Q70 | 5.78 | 1A28 | 3.51 | 1MD3 | 4.25 | 1O4J | 3.66 | 1BM6 | 3.95 | 830C |
| EF-150 | 2.49 | 2ITY | 3.60 | 1QKT | 5.98 | 1A28 | 3.76 | 1MD3 | 1.93 | 1YOM | 3.55 | 1BM6 | 2.31 | 830C |
| EF-151 | 5.63 | 2ITY | 1.78 | 1QKT | 7.68 | 1A28 | 3.62 | 1MD3 | 3.15 | 1YOM | 3.25 | 1BM6 | 2.88 | 830C |
| EF-152 | 4.28 | 1M17 | 3.30 | 2Q70 | 4.29 | 1A28 | 4.25 | 1LBK | 3.26 | 1YOM | 3.83 | 1BM6 | 3.15 | 1EUB |
| EF-153 | 4.05 | 1M17 | 3.89 | 1QKU | 4.40 | 1A28 | 5.16 | 1LBK | 2.93 | 2BDJ | 2.85 | 1BM6 | 2.46 | 830C |
| EF-154 | 4.01 | 2ITY | 4.70 | 1QKT | 4.36 | 1A28 | 3.44 | 1LBK | 3.56 | 2H8H | 2.66 | 1BM6 | 3.96 | 1EUB |
| EF-155 | 2.79 | 2ITY | 6.36 | 1QKT | 5.43 | 2OVM | 3.16 | 19GS | 4.24 | 1O4J | 3.39 | 1BM6 | 2.79 | 830C |
| EF-156 | 2.72 | 2ITY | 3.96 | 1QKT | 4.88 | 1A28 | 4.07 | 19GS | 3.48 | 2BDJ | 3.80 | 1BM6 | 2.09 | 830C |
| EF-157 | 2.38 | 2ITY | 2.67 | 1QKT | 4.62 | 1A28 | 2.99 | 1LBK | 1.84 | 1YOM | 2.28 | 1BM6 | 1.72 | 830C |
| EF-158 | 4.83 | 1M17 | 3.13 | 2Q70 | 4.62 | 1A28 | 3.08 | 1LBK | 4.05 | 2BDJ | 3.44 | 1BM6 | 2.84 | 1EUB |
| EF-159 | 2.60 | 1M17 | 3.97 | 2Q70 | 4.93 | 1SQN | 3.35 | 1LBK | 3.08 | 2BDJ | 3.29 | 1BM6 | 3.28 | 830C |
| EF-160 | 3.64 | 1M17 | 6.98 | 1QKT | 6.51 | 1A28 | 4.28 | 1LBK | 3.34 | 2BDJ | 2.96 | 1BM6 | 3.13 | 830C |
| EF-161 | 4.20 | 2ITY | 4.63 | 1QKT | 5.53 | 1A28 | 3.70 | 1LBK | 3.11 | 1YOM | 3.37 | 1BM6 | 3.98 | 1EUB |
| EF-162 | 2.57 | 1M17 | 4.03 | 1G50 | 4.52 | 2OVH | 3.77 | 1LBK | 4.78 | 2BDJ | 2.64 | 1BM6 | 2.55 | 1EUB |
| EF-163 | 4.27 | 2ITY | 4.39 | 1QKT | 6.57 | 1A28 | 4.32 | 1MD3 | 3.96 | 1O4J | 3.41 | 1BM6 | 2.36 | 830C |
| EF-164 | 2.20 | 2ITY | 4.05 | 1G50 | 5.39 | 1SQN | 4.11 | 1LBK | 3.11 | 2H8H | 3.62 | 1BM6 | 2.30 | 830C |
| EF-165 | 2.84 | 2ITY | 2.71 | 2Q70 | 5.13 | 1A28 | 3.53 | 19GS | 3.25 | 1O4J | 2.81 | 1BM6 | 2.53 | 830C |
| EF-166 | 3.12 | 1M17 | 1.19 | 1QKT | 5.85 | 2OVH | 3.89 | 1LBK | 3.43 | 1O4J | 3.04 | 1BM6 | 3.12 | 830C |
| EF-167 | 2.43 | 2ITY | - | 1QKU | 4.39 | 2OVH | 3.94 | 1MD3 | 3.38 | 2BDJ | 3.78 | 1BM6 | 2.79 | 830C |
| EF-168 | 3.21 | 1M17 | 3.94 | 2Q70 | 4.97 | 2OVM | 2.61 | 19GS | 3.45 | 1O4J | 2.85 | 1BM6 | 3.77 | 830C |
| EF-169 | 2.43 | 2ITY | 4.84 | 1G50 | 4.46 | 2OVH | 3.38 | 1MD3 | 3.20 | 1YOM | 3.31 | 1BM6 | 3.53 | 830C |
| EF-170 | 4.61 | 2ITY | 6.18 | 2Q70 | 5.09 | 1SQN | 3.83 | 1LBK | 5.46 | 1YOM | 4.98 | 1BM6 | 4.06 | 830C |
| EF-171 | 3.46 | 1M17 | 6.39 | 2Q70 | 6.46 | 1SQN | 3.18 | 19GS | 4.43 | 1YOM | 4.37 | 1BM6 | 3.79 | 1EUB |
| EF-172 | 6.01 | 2ITY | 6.38 | 2Q70 | 5.21 | 1SQN | 3.16 | 19GS | 4.79 | 1YOM | 4.47 | 1BM6 | 4.04 | 1EUB |
| EF-173 | 3.28 | 2ITY | 6.38 | 1QKT | 2.77 | 2OVM | 3.14 | 1MD3 | 5.30 | 2H8H | 5.56 | 1BM6 | 2.98 | 830C |
| EF-174 | 2.19 | 2ITY | - | 1QKT | 2.36 | 2OVH | 3.13 | 1LBK | 2.88 | 1O4J | 3.81 | 1BM6 | 1.47 | 830C |
| EF-175 | 4.43 | 1M17 | - | 1QKT | 1.49 | 2OVH | 3.11 | 19GS | 2.98 | 2BDJ | 2.90 | 1BM6 | 3.86 | 830C |
| EF-176 | 3.92 | 1M17 | 1.57 | 1QKT | 0.83 | 2OVH | 3.09 | 19GS | 4.74 | 1YOM | 2.79 | 1BM6 | 4.46 | 1EUB |
| EF-177 | 3.24 | 1M17 | 5.26 | 1QKU | 4.49 | 2OVM | 3.07 | 19GS | 3.53 | 2BDJ | 3.64 | 1BM6 | 5.84 | 1EUB |

| No. | PPARG | | BMP2 | | CCNA2 | | KDR | | TGFBR1 | | CASP7 | |
| --- | --- | --- | --- | --- | --- | --- | --- | --- | --- | --- | --- | --- |
|  | Score | PDB | Score | PDB | Score | PDB | Score | PDB | Score | PDB | Score | PDB |
| EF-001 | 0.64 | 2PRG | 2.45 | 1REU | 4.62 | 2BPM | 3.24 | 3CJF | 5.50 | 1RW8 | 4.97 | 1SHL |
| EF-002 | 5.97 | 2PRG | 4.50 | 3BMP | 5.31 | 2UZD | 5.27 | 2P2H | 4.41 | 1VJY | 5.59 | 1SHL |
| EF-003 | 6.70 | 2PRG | 3.40 | 3BMP | 7.89 | 2BPM | 6.58 | 3CJF | 3.31 | 1VJY | 6.15 | 1SHL |
| EF-004 | 3.04 | 2PRG | 2.78 | 3BMP | 5.16 | 1H1P | 4.27 | 2P2H | 5.53 | 1RW8 | 4.68 | 1SHL |
| EF-005 | - | 2PRG | 3.25 | 3BMP | 6.08 | 2BPM | 5.21 | 3CJF | 4.30 | 1VJY | 6.42 | 1SHL |
| EF-006 | 0.98 | 2PRG | 2.14 | 1REU | 3.82 | 2BPM | 3.51 | 3CJF | 4.85 | 1RW8 | 4.30 | 1SHL |
| EF-007 | 1.47 | 2PRG | 1.78 | 3BMP | 4.54 | 1H1P | 2.72 | 2P2H | 5.28 | 1RW8 | 3.85 | 1SHL |
| EF-008 | 0.13 | 1FM6 | 2.26 | 1REU | 4.56 | 1H1P | 2.98 | 3CJF | 1.12 | 1RW8 | 4.09 | 1SHL |
| EF-009 | 0.64 | 1ZGY | 2.71 | 1REU | 3.83 | 2BPM | 2.81 | 3CJF | 2.47 | 1VJY | 4.20 | 1SHL |
| EF-010 | 1.79 | 3IA6 | 1.98 | 1REU | 6.6 | 2BPM | 2.43 | 3CJF | 3.15 | 1RW8 | 4.99 | 1SHL |
| EF-011 | 6.85 | 3IA6 | 3.66 | 3BMP | 4.66 | 2BPM | 8.65 | 2P2H | 8.13 | 1VJY | 9.49 | 1SHL |
| EF-012 | - | 1FM6 | 1.76 | 1REU | 3.82 | 1H1P | 3.57 | 3CJF | 1.72 | 1VJY | 4.13 | 1SHL |
| EF-013 | 8.01 | 3IA6 | 5.38 | 3BMP | 7.99 | 2UZD | 8.37 | 3CJF | 4.47 | 1VJY | 8.79 | 1SHL |
| EF-014 | 7.24 | 3IA6 | 4.07 | 3BMP | 7.03 | 2UZD | 4.74 | 2P2H | - | 1VJY | 7.37 | 1SHL |
| EF-015 | 6.82 | 2PRG | 3.31 | 3BMP | 8.51 | 1VYW | 6.99 | 3CJF | 9.53 | 1VJY | 9.55 | 1SHL |
| EF-016 | 5.00 | 2PRG | 5.84 | 3BMP | 8.78 | 1VYW | 7.93 | 2P2H | 7.00 | 1VJY | 7.77 | 1SHL |
| EF-017 | 4.64 | 3IA6 | 3.09 | 3BMP | 5.54 | 1VYW | 2.94 | 2P2H | 1.48 | 1VJY | 2.86 | 1SHL |
| EF-018 | 0.19 | 2PRG | 2.58 | 3BMP | 5.22 | 2BPM | 2.87 | 3CJF | 2.36 | 1RW8 | 4.33 | 1SHL |
| EF-019 | 2.29 | 3IA6 | 2.42 | 1REU | 7.23 | 2BPM | 3.44 | 3CJF | 5.25 | 1VJY | 5.28 | 1SHL |
| EF-020 | 2.12 | 1FM6 | 1.59 | 1REU | 7.06 | 2BPM | 3.20 | 3CJF | 5.20 | 1VJY | 4.76 | 1SHL |
| EF-021 | 3.69 | 1ZGY | 3.23 | 1REU | 7.7 | 1VYW | 8.49 | 3CJF | 3.13 | 1VJY | 10.43 | 1SHL |
| EF-022 | 3.03 | 3IA6 | 2.61 | 1REU | 3.69 | 2BPM | 4.75 | 2P2H | 5.82 | 1VJY | 5.86 | 1SHL |
| EF-023 | 0.53 | 2PRG | 2.37 | 1REU | 3.12 | 2UZD | 3.01 | 2P2H | 3.86 | 1VJY | 7.14 | 1SHL |
| EF-024 | 6.78 | 2PRG | 3.81 | 3BMP | 6.27 | 2BPM | 5.55 | 2P2H | 5.35 | 1VJY | 8.13 | 1SHL |
| EF-025 | 0.64 | 3IA6 | 2.18 | 1REU | 3.41 | 2BPM | 4.21 | 3CJF | 5.83 | 1VJY | 5.98 | 1SHL |
| EF-026 | 4.37 | 3IA6 | 4.31 | 1REU | 6.65 | 2BPM | 7.56 | 2P2H | 3.15 | 1VJY | 10.38 | 1SHL |
| EF-027 | 0.41 | 1FM6 | 3.53 | 3BMP | 5.66 | 2BPM | 5.40 | 3CJF | 2.95 | 1VJY | 5.98 | 1SHL |
| EF-028 | 2.41 | 3IA6 | 2.01 | 3BMP | 5.4 | 2BPM | 3.12 | 3CJF | 3.10 | 1VJY | 3.19 | 1SHL |
| EF-029 | 4.71 | 2PRG | 4.67 | 1REU | 5.7 | 2BPM | 6.45 | 3CJF | 3.90 | 1VJY | 8.76 | 1SHL |
| EF-030 | 6.97 | 2PRG | 3.22 | 1REU | 6.7 | 2UZD | 6.33 | 2P2H | 5.29 | 1RW8 | 7.99 | 1SHL |
| EF-031 | 5.62 | 2PRG | 2.02 | 3BMP | 5.12 | 2BPM | 7.24 | 3CJF | 3.04 | 1VJY | 8.80 | 1SHL |
| EF-032 | 8.20 | 3IA6 | 4.83 | 3BMP | 6.66 | 1VYW | 9.59 | 2P2H | 5.02 | 1VJY | 8.04 | 1SHL |
| EF-033 | 0.08 | 3IA6 | 3.66 | 3BMP | 6.86 | 2UZD | 6.08 | 2P2H | 2.29 | 1VJY | 8.10 | 1SHL |
| EF-034 | 2.51 | 3IA6 | 3.01 | 1REU | 3.79 | 2BPM | 5.87 | 2P2H | 4.30 | 1RW8 | 3.97 | 1SHL |
| EF-035 | 3.94 | 3IA6 | 3.88 | 1REU | 6.27 | 2BPM | 5.67 | 2P2H | 4.44 | 1VJY | 4.80 | 1SHL |
| EF-036 | 3.15 | 2PRG | 2.35 | 1REU | 2.3 | 1VYW | 6.08 | 3CJF | - | 1VJY | 5.76 | 1SHL |
| EF-037 | 7.58 | 2PRG | 4.06 | 1REU | 9.38 | 1H1P | 7.19 | 2P2H | 4.79 | 1RW8 | 6.88 | 1SHL |
| EF-038 | 7.80 | 2PRG | 5.54 | 3BMP | 7.71 | 2BPM | 7.79 | 2P2H | 6.32 | 1RW8 | 6.29 | 1SHL |
| EF-039 | 1.30 | 3IA6 | 1.81 | 3BMP | 5.54 | 1VYW | 5.76 | 2P2H | 5.18 | 1RW8 | 7.39 | 1SHL |
| EF-040 | 3.41 | 3IA6 | 4.21 | 3BMP | 5.94 | 2BPM | 5.34 | 2P2H | 0.62 | 1VJY | 9.35 | 1SHL |
| EF-041 | 3.56 | 1FM6 | 2.37 | 3BMP | 4.08 | 1VYW | 6.05 | 3CJF | 5.05 | 1VJY | 7.81 | 1SHL |
| EF-042 | - | 1ZGY | 3.14 | 3BMP | 4.37 | 1H1P | 5.96 | 3CJF | 2.46 | 1RW8 | 7.82 | 1SHL |
| EF-043 | 3.77 | 3IA6 | 2.67 | 3BMP | 6.66 | 1VYW | 6.24 | 3CJF | 4.69 | 1VJY | 11.75 | 1SHL |
| EF-044 | 4.61 | 2PRG | 4.18 | 3BMP | 4.87 | 2BPM | 5.20 | 3CJF | 5.71 | 1VJY | 8.63 | 1SHL |
| EF-045 | - | 2PRG | 4.10 | 3BMP | 4.24 | 2BPM | 5.01 | 2P2H | 1.42 | 1VJY | 9.86 | 1SHL |
| EF-046 | 6.05 | 2PRG | 1.75 | 1REU | 9.56 | 2UZD | 9.30 | 2P2H | 6.99 | 1VJY | 11.48 | 1SHL |
| EF-047 | - | 2PRG | 2.84 | 1REU | - | 2UZD | 5.48 | 3CJF | - | 1VJY | 6.84 | 1SHL |
| EF-048 | 0.24 | 3IA6 | 2.44 | 1REU | 0.09 | 1VYW | 4.66 | 3CJF | - | 1RW8 | 7.92 | 1SHL |
| EF-049 | - | 2PRG | 2.05 | 1REU | 1.25 | 2UZD | 2.78 | 3CJF | - | 1RW8 | 8.13 | 1SHL |
| EF-050 | - | 2PRG | 3.69 | 3BMP | 1.69 | 2BPM | 3.92 | 3CJF | - | 1VJY | 8.86 | 1SHL |
| EF-051 | 4.01 | 3IA6 | 3.56 | 1REU | 4.94 | 2BPM | 4.87 | 3CJF | 2.09 | 1VJY | 4.35 | 1SHL |
| EF-052 | 2.61 | 3IA6 | 3.58 | 1REU | 4.24 | 1VYW | 4.30 | 3CJF | 3.22 | 1RW8 | 5.49 | 1SHL |
| EF-053 | 3.46 | 2PRG | 4.42 | 1REU | 4.87 | 1VYW | 3.53 | 3CJF | 3.46 | 1RW8 | 6.20 | 1SHL |
| EF-054 | 3.25 | 1ZGY | 4.64 | 1REU | 5.94 | 1H1P | 4.20 | 3CJF | 0.13 | 1VJY | 6.15 | 1SHL |
| EF-055 | 4.82 | 1ZGY | 4.13 | 3BMP | 5.18 | 1H1P | 3.45 | 2P2H | 4.37 | 1VJY | 5.75 | 1SHL |
| EF-056 | 4.21 | 2PRG | 3.22 | 1REU | 5.57 | 1H1P | 4.73 | 2P2H | 4.64 | 1VJY | 4.32 | 1SHL |
| EF-057 | 4.37 | 2PRG | 3.33 | 1REU | 5.29 | 1H1P | 4.85 | 2P2H | 4.88 | 1RW8 | 4.30 | 1SHL |
| EF-058 | 4.06 | 2PRG | 3.46 | 1REU | 4.2 | 1H1P | 4.44 | 3CJF | 5.15 | 1RW8 | 5.79 | 1SHL |
| EF-059 | 3.98 | 2PRG | 2.49 | 1REU | 5.22 | 1H1P | 3.36 | 3CJF | 3.15 | 1RW8 | 4.21 | 1SHL |
| EF-060 | 4.05 | 1FM6 | 2.61 | 1REU | 5.65 | 1H1P | 3.69 | 2P2H | 3.86 | 1VJY | 5.70 | 1SHL |
| EF-061 | 2.77 | 2PRG | 2.73 | 1REU | 5.33 | 2BPM | 4.79 | 2P2H | 1.92 | 1VJY | 5.95 | 1SHL |
| EF-062 | 3.40 | 1ZGY | 4.79 | 1REU | 5.52 | 2UZD | 4.01 | 3CJF | 1.47 | 1RW8 | 5.58 | 1SHL |
| EF-063 | 3.50 | 2PRG | 3.61 | 1REU | 5.76 | 1VYW | 3.06 | 2P2H | 1.69 | 1RW8 | 5.82 | 1SHL |
| EF-064 | 2.29 | 2PRG | 2.63 | 1REU | 5.42 | 1VYW | 4.83 | 2P2H | 3.97 | 1RW8 | 5.32 | 1SHL |
| EF-065 | 4.08 | 2PRG | 2.90 | 1REU | 3.88 | 2BPM | 3.37 | 3CJF | 2.47 | 1VJY | 3.59 | 1SHL |
| EF-066 | 5.59 | 2PRG | 2.75 | 1REU | 4.08 | 2BPM | 3.56 | 2P2H | 2.92 | 1RW8 | 3.84 | 1SHL |
| EF-067 | 5.39 | 2PRG | 2.99 | 1REU | 4.64 | 2BPM | 4.11 | 3CJF | 3.27 | 1VJY | 4.29 | 1SHL |
| EF-068 | 4.52 | 2PRG | 2.06 | 1REU | 4.55 | 1H1P | 3.55 | 2P2H | 4.75 | 1VJY | 4.32 | 1SHL |
| EF-069 | 3.83 | 2PRG | 2.73 | 1REU | 5.35 | 1H1P | 3.20 | 3CJF | 2.41 | 1RW8 | 3.89 | 1SHL |
| EF-070 | 4.22 | 3IA6 | 1.80 | 3BMP | 5.25 | 1H1P | 4.13 | 2P2H | 3.24 | 1RW8 | 4.61 | 1SHL |
| EF-071 | 4.52 | 3IA6 | 3.26 | 1REU | 4.32 | 1H1P | 2.67 | 2P2H | 5.39 | 1VJY | 4.52 | 1SHL |
| EF-072 | 5.00 | 2PRG | 2.71 | 1REU | 4.98 | 1H1P | 3.85 | 3CJF | 2.39 | 1VJY | 4.55 | 1SHL |
| EF-073 | 2.13 | 2PRG | 1.98 | 1REU | 3.09 | 1VYW | 3.85 | 2P2H | - | 1VJY | 3.66 | 1SHL |
| EF-074 | 0.70 | 3IA6 | 0.32 | 1REU | 2.03 | 2BPM | 3.79 | 3CJF | - | 1VJY | 2.49 | 1SHL |
| EF-075 | 5.23 | 2PRG | 3.14 | 1REU | 5.39 | 1H1P | 4.02 | 2P2H | 4.04 | 1VJY | 4.57 | 1SHL |
| EF-076 | 5.91 | 1ZGY | 2.63 | 1REU | 4.53 | 2BPM | 3.19 | 2P2H | 1.24 | 1RW8 | 6.03 | 1SHL |
| EF-077 | 4.27 | 2PRG | 2.74 | 1REU | 6.13 | 1H1P | 3.36 | 2P2H | 6.43 | 1VJY | 5.47 | 1SHL |
| EF-078 | - | 3IA6 | - | 1REU | 0.93 | 2BPM | 2.64 | 3CJF | - | 1RW8 | 1.26 | 1SHL |
| EF-079 | 0.86 | 2PRG | 1.72 | 1REU | 4.96 | 1H1P | 2.69 | 3CJF | 2.65 | 1RW8 | 4.76 | 1SHL |
| EF-080 | 1.69 | 2PRG | 2.06 | 3BMP | 3.61 | 1H1P | 2.23 | 3CJF | - | 1RW8 | 4.66 | 1SHL |
| EF-081 | 2.93 | 3IA6 | 1.24 | 1REU | 3.99 | 1H1P | 2.48 | 3CJF | - | 1RW8 | 3.97 | 1SHL |
| EF-082 | 3.33 | 2PRG | 2.27 | 1REU | 5.09 | 1H1P | 3.89 | 3CJF | 2.96 | 1RW8 | 4.59 | 1SHL |
| EF-083 | 3.22 | 2PRG | 2.75 | 1REU | 2.31 | 2BPM | 3.71 | 2P2H | 1.67 | 1RW8 | 5.29 | 1SHL |
| EF-084 | 2.82 | 2PRG | 2.73 | 3BMP | 4.13 | 1H1P | 3.71 | 3CJF | 4.11 | 1RW8 | 4.24 | 1SHL |
| EF-085 | 2.46 | 2PRG | 2.42 | 1REU | 5.36 | 1H1P | 3.72 | 3CJF | 2.04 | 1RW8 | 5.44 | 1SHL |
| EF-086 | 4.43 | 3IA6 | 2.82 | 1REU | 4.82 | 1H1P | 4.16 | 3CJF | 3.15 | 1RW8 | 5.02 | 1SHL |
| EF-087 | 3.67 | 2PRG | 2.32 | 1REU | 5.35 | 1H1P | 4.62 | 3CJF | 2.92 | 1RW8 | 4.64 | 1SHL |
| EF-088 | 3.14 | 3IA6 | 3.11 | 1REU | 3.89 | 2BPM | 6.08 | 3CJF | 0.07 | 1RW8 | 5.91 | 1SHL |
| EF-089 | 4.01 | 2PRG | 2.84 | 3BMP | 6.73 | 2UZD | 5.18 | 2P2H | 7.83 | 1VJY | 3.12 | 1SHL |
| EF-090 | 4.57 | 3IA6 | 3.41 | 3BMP | 6.24 | 2UZD | 4.63 | 2P2H | 7.50 | 1VJY | 4.76 | 1SHL |
| EF-091 | 3.93 | 3IA6 | 3.44 | 3BMP | 6.03 | 1H1P | 3.48 | 2P2H | 4.86 | 1VJY | 7.33 | 1SHL |
| EF-092 | 5.60 | 2PRG | 3.26 | 3BMP | 6.35 | 2BPM | 5.84 | 2P2H | 6.19 | 1VJY | 7.30 | 1SHL |
| EF-093 | 3.05 | 3IA6 | 2.98 | 3BMP | 4.5 | 1H1P | 2.69 | 2P2H | - | 1RW8 | 4.47 | 1SHL |
| EF-094 | 2.86 | 2PRG | 3.14 | 1REU | 4.26 | 1VYW | 4.45 | 3CJF | 0.28 | 1VJY | 4.72 | 1SHL |
| EF-095 | 3.55 | 2PRG | 2.64 | 1REU | 5.14 | 2BPM | 4.46 | 3CJF | 4.40 | 1RW8 | 5.06 | 1SHL |
| EF-096 | 3.71 | 3IA6 | 2.76 | 1REU | 5.13 | 1VYW | 4.34 | 3CJF | 2.87 | 1RW8 | 6.15 | 1SHL |
| EF-097 | 3.68 | 3IA6 | 2.05 | 3BMP | 5.75 | 1H1P | 2.45 | 2P2H | 2.27 | 1VJY | 4.81 | 1SHL |
| EF-098 | 3.35 | 1FM6 | 2.23 | 3BMP | 4.01 | 2BPM | 3.49 | 2P2H | 1.61 | 1VJY | 4.68 | 1SHL |
| EF-099 | 5.69 | 2PRG | 2.73 | 1REU | 5.21 | 1H1P | 5.05 | 2P2H | 5.22 | 1VJY | 4.37 | 1SHL |
| EF-100 | 3.73 | 2PRG | 2.66 | 1REU | 5.34 | 1VYW | 4.43 | 2P2H | 1.87 | 1VJY | 6.13 | 1SHL |
| EF-101 | 3.67 | 2PRG | 2.94 | 3BMP | 2.67 | 1H1P | 4.77 | 2P2H | 4.55 | 1VJY | 4.14 | 1SHL |
| EF-102 | 3.91 | 2PRG | 3.53 | 3BMP | 5.8 | 1H1P | 6.08 | 2P2H | 2.12 | 1RW8 | 4.76 | 1SHL |
| EF-103 | 4.59 | 1ZGY | 2.83 | 1REU | 4.93 | 1H1P | 4.16 | 3CJF | 3.66 | 1RW8 | 5.69 | 1SHL |
| EF-104 | 4.09 | 2PRG | 2.81 | 1REU | 5.05 | 1H1P | 4.01 | 2P2H | 3.60 | 1RW8 | 6.10 | 1SHL |
| EF-105 | 4.37 | 2PRG | 2.26 | 1REU | 6.58 | 1H1P | 3.68 | 3CJF | 1.88 | 1RW8 | 5.29 | 1SHL |
| EF-106 | 5.37 | 2PRG | 3.54 | 1REU | 5.96 | 1H1P | 3.63 | 3CJF | 2.77 | 1RW8 | 4.76 | 1SHL |
| EF-107 | 4.37 | 1FM6 | 3.26 | 3BMP | 6.19 | 1VYW | 4.30 | 3CJF | 1.99 | 1RW8 | 5.27 | 1SHL |
| EF-108 | 4.47 | 2PRG | 3.36 | 3BMP | 5.32 | 1VYW | 5.33 | 2P2H | 3.88 | 1VJY | 4.23 | 1SHL |
| EF-109 | 5.19 | 1FM6 | 3.03 | 3BMP | 4.73 | 1H1P | 3.20 | 2P2H | 3.96 | 1VJY | 4.35 | 1SHL |
| EF-110 | 3.58 | 1FM6 | 3.25 | 1REU | 6.18 | 1H1P | 5.70 | 2P2H | 3.33 | 1RW8 | 5.10 | 1SHL |
| EF-111 | 4.48 | 2PRG | 2.74 | 1REU | 5.19 | 1H1P | 6.15 | 2P2H | 3.45 | 1VJY | 4.74 | 1SHL |
| EF-112 | 4.00 | 2PRG | 2.75 | 3BMP | 6.81 | 1H1P | 3.34 | 2P2H | 3.13 | 1VJY | 4.35 | 1SHL |
| EF-113 | 3.17 | 1ZGY | 3.00 | 1REU | 5.24 | 2BPM | 2.80 | 3CJF | - | 1RW8 | 5.68 | 1SHL |
| EF-114 | 2.94 | 2PRG | 2.44 | 1REU | 6.08 | 1H1P | 2.66 | 2P2H | 2.06 | 1RW8 | 5.60 | 1SHL |
| EF-115 | 3.27 | 2PRG | 1.44 | 3BMP | 4.28 | 2BPM | 3.53 | 3CJF | - | 1RW8 | 5.47 | 1SHL |
| EF-116 | 3.87 | 2PRG | 2.28 | 1REU | 4.7 | 1H1P | 3.66 | 3CJF | 0.03 | 1RW8 | 5.34 | 1SHL |
| EF-117 | 3.40 | 1ZGY | 2.46 | 1REU | 5.47 | 2BPM | 3.18 | 3CJF | - | 1RW8 | 5.08 | 1SHL |
| EF-118 | 2.46 | 2PRG | 3.35 | 1REU | 3.4 | 2BPM | 3.37 | 3CJF | 2.05 | 1RW8 | 4.26 | 1SHL |
| EF-119 | 0.65 | 2PRG | 3.43 | 1REU | 3.67 | 1H1P | 3.50 | 2P2H | 0.86 | 1RW8 | 6.53 | 1SHL |
| EF-120 | 1.43 | 2PRG | 1.83 | 1REU | 4.06 | 1H1P | 2.76 | 2P2H | 0.39 | 1RW8 | 5.24 | 1SHL |
| EF-121 | 1.32 | 3IA6 | 2.57 | 3BMP | 4.42 | 1H1P | 3.01 | 2P2H | - | 1VJY | 3.96 | 1SHL |
| EF-122 | 3.47 | 3IA6 | 2.34 | 1REU | 4.23 | 2BPM | 2.18 | 2P2H | - | 1VJY | 4.85 | 1SHL |
| EF-123 | 0.54 | 2PRG | 2.50 | 1REU | 6.04 | 1H1P | 3.46 | 2P2H | 1.32 | 1RW8 | 3.39 | 1SHL |
| EF-124 | 2.74 | 1FM6 | 2.46 | 1REU | 7.09 | 1H1P | 4.21 | 2P2H | 0.55 | 1RW8 | 5.10 | 1SHL |
| EF-125 | 1.61 | 1FM6 | 2.42 | 3BMP | 5.44 | 1H1P | 3.05 | 3CJF | - | 1RW8 | 3.98 | 1SHL |
| EF-126 | 3.00 | 1FM6 | 1.76 | 1REU | 4.58 | 2BPM | 2.49 | 3CJF | 0.58 | 1RW8 | 6.37 | 1SHL |
| EF-127 | - | 1FM6 | 2.35 | 1REU | 4.51 | 1VYW | 3.57 | 3CJF | - | 1RW8 | 4.45 | 1SHL |
| EF-128 | 0.31 | 1FM6 | 2.35 | 1REU | 4.89 | 1H1P | 2.46 | 3CJF | - | 1RW8 | 5.11 | 1SHL |
| EF-129 | 2.28 | 2PRG | 1.70 | 1REU | 6.74 | 1H1P | 2.91 | 3CJF | - | 1VJY | 4.93 | 1SHL |
| EF-130 | 1.25 | 3IA6 | 1.96 | 1REU | 6.3 | 1H1P | 2.93 | 2P2H | 1.68 | 1RW8 | 4.95 | 1SHL |
| EF-131 | 1.07 | 2PRG | 1.39 | 1REU | 5.36 | 1H1P | 4.22 | 2P2H | 4.62 | 1RW8 | 4.44 | 1SHL |
| EF-132 | 0.72 | 1FM6 | 2.77 | 1REU | 4.12 | 1H1P | 2.72 | 3CJF | 1.04 | 1RW8 | 4.85 | 1SHL |
| EF-133 | 1.39 | 1FM6 | 2.66 | 1REU | 4.09 | 2BPM | 2.47 | 2P2H | - | 1VJY | 5.38 | 1SHL |
| EF-134 | 2.35 | 1FM6 | 2.90 | 1REU | 5.23 | 1H1P | 3.78 | 2P2H | - | 1VJY | 5.60 | 1SHL |
| EF-135 | 1.81 | 1FM6 | 2.77 | 1REU | 5.22 | 2BPM | 2.80 | 2P2H | 1.65 | 1RW8 | 5.20 | 1SHL |
| EF-136 | 3.17 | 1FM6 | 2.89 | 1REU | 4.49 | 1H1P | 3.47 | 2P2H | 0.35 | 1VJY | 5.72 | 1SHL |
| EF-137 | 2.91 | 2PRG | 2.27 | 1REU | 4.72 | 1H1P | 3.82 | 2P2H | 0.85 | 1RW8 | 5.73 | 1SHL |
| EF-138 | - | 3IA6 | 1.09 | 1REU | 5.75 | 1H1P | 2.59 | 3CJF | 0.25 | 1RW8 | 5.14 | 1SHL |
| EF-139 | 0.22 | 1FM6 | 1.50 | 1REU | 5 | 1H1P | 4.78 | 3CJF | 1.54 | 1RW8 | 4.41 | 1SHL |
| EF-140 | 0.88 | 2PRG | 2.10 | 1REU | 5.57 | 1H1P | 4.28 | 3CJF | 3.03 | 1RW8 | 4.53 | 1SHL |
| EF-141 | 3.21 | 2PRG | 2.35 | 1REU | 4.98 | 1H1P | 2.88 | 3CJF | 1.40 | 1RW8 | 6.41 | 1SHL |
| EF-142 | 4.88 | 1FM6 | 2.08 | 1REU | 5.91 | 1H1P | 3.47 | 2P2H | 4.00 | 1RW8 | 5.62 | 1SHL |
| EF-143 | 2.73 | 2PRG | 1.70 | 1REU | 7.37 | 1H1P | 3.08 | 3CJF | 0.91 | 1RW8 | 4.46 | 1SHL |
| EF-144 | 0.42 | 1FM6 | 2.14 | 1REU | 6.16 | 2BPM | 3.90 | 2P2H | - | 1RW8 | 3.44 | 1SHL |
| EF-145 | 2.60 | 2PRG | 1.95 | 1REU | 4.45 | 1H1P | 3.56 | 2P2H | 0.61 | 1RW8 | 4.03 | 1SHL |
| EF-146 | 2.18 | 1ZGY | 2.22 | 1REU | 5.78 | 1H1P | 3.73 | 2P2H | - | 1VJY | 6.52 | 1SHL |
| EF-147 | 2.71 | 1FM6 | 2.95 | 1REU | 6.17 | 1H1P | 4.05 | 2P2H | 1.11 | 1VJY | 5.57 | 1SHL |
| EF-148 | - | 1FM6 | 1.17 | 1REU | 4.3 | 2BPM | 1.74 | 3CJF | - | 1RW8 | 3.71 | 1SHL |
| EF-149 | - | 1FM6 | 2.19 | 3BMP | 2.94 | 2BPM | 2.92 | 2P2H | - | 1RW8 | 3.34 | 1SHL |
| EF-150 | 0.07 | 1FM6 | 1.57 | 1REU | 4.03 | 2BPM | 2.86 | 3CJF | 0.15 | 1RW8 | 4.83 | 1SHL |
| EF-151 | 1.63 | 2PRG | 1.94 | 1REU | 2.93 | 2BPM | 2.34 | 3CJF | - | 1RW8 | 4.22 | 1SHL |
| EF-152 | 1.44 | 1FM6 | 2.90 | 1REU | 3.81 | 2BPM | 2.82 | 2P2H | 0.99 | 1VJY | 5.36 | 1SHL |
| EF-153 | 2.46 | 1ZGY | 2.84 | 1REU | 3.28 | 2BPM | 3.65 | 3CJF | 0.50 | 1RW8 | 4.39 | 1SHL |
| EF-154 | 2.67 | 1ZGY | 2.15 | 1REU | 5.18 | 1H1P | 4.23 | 2P2H | 2.01 | 1RW8 | 4.08 | 1SHL |
| EF-155 | 2.89 | 2PRG | 2.78 | 1REU | 3.69 | 2BPM | 3.48 | 3CJF | 3.28 | 1VJY | 5.82 | 1SHL |
| EF-156 | 4.07 | 3IA6 | 2.43 | 1REU | 4.47 | 2BPM | 2.64 | 2P2H | 0.39 | 1VJY | 4.25 | 1SHL |
| EF-157 | 1.86 | 3IA6 | 1.38 | 1REU | 2.05 | 2BPM | 3.32 | 3CJF | - | 1RW8 | 3.57 | 1SHL |
| EF-158 | 0.95 | 2PRG | 2.44 | 1REU | 4.53 | 1VYW | 2.57 | 2P2H | - | 1RW8 | 4.63 | 1SHL |
| EF-159 | 4.30 | 2PRG | 2.62 | 1REU | 3.72 | 1VYW | 3.82 | 3CJF | 2.64 | 1RW8 | 4.52 | 1SHL |
| EF-160 | - | 1FM6 | 2.14 | 3BMP | 4.56 | 2BPM | 3.33 | 3CJF | 0.60 | 1RW8 | 4.80 | 1SHL |
| EF-161 | 1.54 | 2PRG | 3.14 | 1REU | 4.31 | 1H1P | 3.75 | 3CJF | 2.60 | 1VJY | 5.44 | 1SHL |
| EF-162 | 2.51 | 1FM6 | 2.64 | 1REU | 5.43 | 1H1P | 4.29 | 3CJF | 1.89 | 1RW8 | 4.15 | 1SHL |
| EF-163 | 2.18 | 1FM6 | 2.81 | 3BMP | 4.96 | 2BPM | 5.34 | 3CJF | - | 1RW8 | 5.29 | 1SHL |
| EF-164 | 4.15 | 3IA6 | 2.38 | 1REU | 3.76 | 2BPM | 3.76 | 3CJF | - | 1VJY | 5.86 | 1SHL |
| EF-165 | - | 1FM6 | 1.94 | 3BMP | 5.93 | 1H1P | 3.81 | 3CJF | 0.47 | 1VJY | 5.92 | 1SHL |
| EF-166 | - | 1FM6 | 1.89 | 1REU | 5.31 | 2BPM | 5.58 | 3CJF | - | 1RW8 | 4.82 | 1SHL |
| EF-167 | 3.31 | 3IA6 | 2.42 | 1REU | 3.44 | 2BPM | 2.92 | 2P2H | 2.31 | 1RW8 | 5.90 | 1SHL |
| EF-168 | 0.72 | 3IA6 | 2.22 | 1REU | 5.07 | 2BPM | 3.77 | 3CJF | - | 1VJY | 4.86 | 1SHL |
| EF-169 | 4.52 | 2PRG | 2.80 | 1REU | 3.02 | 1VYW | 2.82 | 3CJF | 2.82 | 1RW8 | 6.39 | 1SHL |
| EF-170 | 3.95 | 2PRG | 2.62 | 1REU | 7.7 | 1H1P | 5.68 | 3CJF | 5.35 | 1VJY | 5.19 | 1SHL |
| EF-171 | 4.96 | 1FM6 | 4.35 | 1REU | 4.53 | 1VYW | 5.98 | 2P2H | 4.11 | 1RW8 | 6.09 | 1SHL |
| EF-172 | 4.33 | 1FM6 | 2.66 | 1REU | 5.93 | 1H1P | 4.10 | 3CJF | 4.17 | 1RW8 | 4.86 | 1SHL |
| EF-173 | - | 3IA6 | 2.79 | 1REU | 4.6 | 2BPM | 3.38 | 2P2H | 3.27 | 1VJY | 5.27 | 1SHL |
| EF-174 | - | 3IA6 | 2.62 | 3BMP | 0.24 | 1H1P | 3.80 | 2P2H | - | 1VJY | 2.76 | 1SHL |
| EF-175 | - | 1FM6 | 3.44 | 3BMP | 1.13 | 2BPM | 4.41 | 2P2H | - | 1RW8 | 6.38 | 1SHL |
| EF-176 | 0.45 | 3IA6 | 1.43 | 3BMP | 4.46 | 2BPM | 2.39 | 2P2H | - | 1RW8 | 5.30 | 1SHL |
| EF-177 | 2.08 | 1ZGY | 2.55 | 1REU | 5.88 | 1H1P | 4.21 | 3CJF | 3.37 | 1RW8 | 4.73 | 1SHL |

“-” means the value less than 0.
